# Large‐scale mitogenome sequencing reveals consecutive expansions of domestic taurine cattle and supports sporadic aurochs introgression

**DOI:** 10.1111/eva.13315

**Published:** 2021-11-27

**Authors:** Vlatka Cubric‐Curik, Dinko Novosel, Vladimir Brajkovic, Omar Rota Stabelli, Stefan Krebs, Johann Sölkner, Dragica Šalamon, Strahil Ristov, Beate Berger, Stamatina Trivizaki, Iosif Bizelis, Maja Ferenčaković, Sophie Rothammer, Elisabeth Kunz, Mojca Simčič, Peter Dovč, Gojko Bunevski, Hysen Bytyqi, Božidarka Marković, Muhamed Brka, Kristaq Kume, Srđan Stojanović, Vasil Nikolov, Natalia Zinovieva, Anna Amanda Schönherz, Bernt Guldbrandtsen, Mato Čačić, Siniša Radović, Preston Miracle, Cristiano Vernesi, Ino Curik, Ivica Medugorac

**Affiliations:** ^1^ Department of Animal Science University of Zagreb Faculty of Agriculture Zagreb Croatia; ^2^ Department of Pathology Croatian Veterinary Institute Zagreb Croatia; ^3^ Department of Sustainable Agro‐Ecosystems and Bioresources, Research and Innovation Centre Fondazione Edmund Mach S. Michele all' Adige Italy; ^4^ Laboratory for Functional Genome Analysis Gene Center Ludwig Maximilians University Munich Munich Germany; ^5^ Division of Livestock Sciences Department of Sustainable Agricultural Systems BOKU‐University of Natural Resources and Life Sciences Vienna Vienna Austria; ^6^ Ruđer Bošković Institute Zagreb Croatia; ^7^ AREC Raumberg‐Gumpenstein Institute of Organic Farming and Biodiversity of Farm Animals Thalheim Austria; ^8^ Institute of Animal Genetic Resources Nea Mesimvria Thessaloniki Greece; ^9^ Faculty of Animal Science and Aquaculture Department of Animal Breeding & Husbandry Agricultural University of Athens Athens Greece; ^10^ Population Genomics Group Faculty of Veterinary Medicine Department of Veterinary Sciences LMU Munich Munich Germany; ^11^ Biotechnical Faculty Department of Animal Science University of Ljubljana Ljubljana Slovenia; ^12^ Faculty of Agricultural Sciences and Food University Ss. Cyril and Methodius Skopje Macedonia; ^13^ Faculty of Agriculture and Veterinary Department of Animal Science University of Prishtina Prishtina Kosovo; ^14^ Biotechnical Faculty Department of Livestock Science University of Montenegro Podgorica Montenegro; ^15^ Faculty of Agriculture and Food Science Institute of Animal Sciences University of Sarajevo Sarajevo Bosnia and Herzegovina; ^16^ ALBAGENE Association Tirana Albania; ^17^ Ministry of Agriculture, Forestry and Water Management Beograd Serbia; ^18^ Executive Agency for Selection and Reproduction in Animal Breeding Sofia Bulgaria; ^19^ Center of Biotechnology and Molecular Diagnostics of the L.K. Ernst Institute of Animal Husbandry Moscow Region Russia; ^20^ Department of Animal Science Aarhus University Tjele Denmark; ^21^ Department of Animal Sciences Rheinische Friedrich‐Wilhelms‐Universität Bonn Bonn Germany; ^22^ Croatian Agricultural Agency Zagreb Croatia; ^23^ Institute for Quaternary Palaeontology and Geology Croatian Academy of Sciences and Arts Zagreb Croatia; ^24^ Department of Archaeology University of Cambridge Cambridge UK

**Keywords:** aurochs introgression, cattle, diversity, domestication, mitogenome, phylogenetics

## Abstract

The contribution of domestic cattle in human societies is enormous, making cattle, along with other essential benefits, the economically most important domestic animal in the world today. To expand existing knowledge on cattle domestication and mitogenome diversity, we performed a comprehensive complete mitogenome analysis of the species (802 sequences, 114 breeds). A large sample was collected in South‐east Europe, an important agricultural gateway to Europe during Neolithization and a region rich in cattle biodiversity. We found 1725 polymorphic sites (810 singletons, 853 parsimony‐informative sites and 57 indels), 701 unique haplotypes, a haplotype diversity of 0.9995 and a nucleotide diversity of 0.0015. In addition to the dominant T_3_ and several rare haplogroups (Q, T_5_, T_4_, T_2_ and T_1_), we have identified maternal line in Austrian Murbodner cattle that possess surviving aurochs’ mitochondria haplotype P_1_ that diverged prior to the Neolithization process. This is convincing evidence for rare female‐mediated adaptive introgression of wild aurochs into domesticated cattle in Europe. We revalidated the existing haplogroup classification and provided Bayesian phylogenetic inference with a more precise estimated divergence time than previously available. Occasionally, classification based on partial mitogenomes was not reliable; for example, some individuals with haplogroups P and T_5_ were not recognized based on D‐loop information. Bayesian skyline plot estimates (median) show that the earliest population growth began before domestication in cattle with haplogroup T_2_, followed by Q (~10.0–9.5 kyBP), whereas cattle with T_3_ (~7.5 kyBP) and T_1_ (~3.0–2.5 kyBP) expanded later. Overall, our results support the existence of interactions between aurochs and cattle during domestication and dispersal of cattle in the past, contribute to the conservation of maternal cattle diversity and enable functional analyses of the surviving aurochs P_1_ mitogenome.

## 1 INTRODUCTION

The domestication of animals and plants is recognized as one of the most influential processes that has shaped the development and growth of human civilization (Larson et al., 2014; Zeder, 2006). The contribution of domestic cattle, which are divided into two major subspecies, the taurine or humpless cattle (*Bos taurus taurus*) and the indicine (Zebu) or humped cattle (*Bos taurus indicus*), to human societies has been enormous because cattle are able to convert large amounts of roughage into high‐quality food, while other benefits (draught power, transport, leather, manure as fertilizer, dry dung for fuel and use of other cattle products) are also substantial (Sherratt, 1983). All modern taurine and indicine cattle are descended from the extinct large‐bodied wild aurochs (*Bos primigenius*), which was widespread throughout much of Eurasia and northern Africa during the Pleistocene and early Holocene (Zeuner, 1963).

Taurine cattle were domesticated from *Bos primigenius primigenius* around 10.5 kiloyears before present (kyBP) in the Fertile Crescent (Helmer et al., 2005), while indicine cattle were domesticated from *Bos primigenius namadicus* about 1500 years later in the Indus Valley in present‐day Pakistan (Meadow, 1993).

In domestic animals and their close relatives, mtDNA sequences were the first source of molecular information used in the early days of ancient DNA analyses. Together with sequences obtained contemporarily, they provided seminal evidence that complemented archaeological findings and shaped our initial understanding of cattle domestication (Bailey et al., 1996; Bradley et al., 1996; Loftus et al., 1994; Mannen et al., 1998). So, the first maternal evidence from the D‐loop mtDNA showed a strong bifurcation between two geographically distinct T and I haplogroups corresponding to the two main subspecies, taurine and indicine cattle, respectively. Since the T and I haplogroups diverged long before domestication (Troy et al., 2001), this was interpreted as genetic evidence of two independent domestication events. Later analyses of complete mitogenomes in modern cattle revealed the existence of other pre‐domestication P, R, Q and C (the later present only in ancient samples from China), and significant diversity within T, so‐called ‘mega T’ haplogroup (T_1_, T_2_, T_3_, T_4_, T_5_ and T_6_), indicating the complex pattern of the domestication process (Achilli et al., 2009; Bonfiglio et al., 2010; Olivieri et al., 2015; Xia et al., 2019; Zhang et al., 2013). Interestingly, haplogroup P, found primarily in ancient bones of large European aurochs pre‐dating the Neolithic, has rarely been found in North‐east Asian cattle and never in contemporary European breeds. The absence of haplogroup P in modern European cattle has been the main argument that domesticated cattle did not interbreed with wild aurochs during Neolithization (Edwards et al., 2007). This contrasts with the pattern supported by research in pigs, sheep and horses that domestication has been gradual with continuous interbreeding between domesticated and wild forms (Frantz et al., 2020; Larson & Burger, 2013).

Turning to the Y chromosome, the presence of three different male‐specific haplogroups, the geographically distinct Y_1_ at North‐west Europe and Y_2_ at South‐east Europe, both found in taurine cattle, and the presence of the specific Y_3_ found in indicine cattle and crosses between African taurine and indicine cattle (Chen et al., 2018; Pérez‐Pardal et al., 2010, 2018), does not contrast with the male‐mediated introgression of northern and central European aurochs with haplogroup Y_1_ into domesticated cattle.

Large‐scale analyses based on high‐throughput genome‐wide nuclear markers show that modern cattle diversity is structured into three major groups representing Eurasian taurine, African taurine and Asian indicine cattle, as well as various types of crosses, all combinations and intricacies, among these three groups (Decker et al., 2014; Kim et al., 2020; McTavish et al., 2013). Chen et al. (2018) showed that indicine cattle can be further subdivided into Indian and Chinese indicines. Recently, genomic analyses of ancient bones have confirmed that interbreeding of local and geographically distinct aurochs with modern cattle has contributed to the genetic diversity of modern cattle (Park et al., 2015; Verdugo et al., 2019). Thus, all the results obtained show that the domestication of cattle is a very complex process that, although well studied, still leaves a number of questions unanswered (Larson & Burger, 2013). A good example is bovine mitogenomics, where there are still a number of open questions concerning the occurrence of rare haplogroups, their geographic and migratory origins in relation to wild ancestors and their demographic patterns.

The main objectives of this study were (i) to provide a more complete description of the classification and phylogeny of rare haplotypes, including the aurochs‐specific P haplogroup, and (ii) to reconstruct demographic events that led to the present haplogroup distributions. To achieve this, we performed a comprehensive complete mitogenome analysis of cattle (>800 mitogenomes—114 known breeds), with a large number of samples collected in South‐east Europe, an important agricultural gateway to Europe during the Neolithization and known for its biodiversity of livestock (Papachristou et al., 2020), which was under‐represented in previous complete mitogenome studies. Overall, our analyses focused (i) on estimating mitogenome diversity in modern cattle, (ii) on identifying a large number of rare haplotypes, (iii) on critically comparing the existing haplogroup classification and the classification obtained by complex Bayesian phylogenetic inference, (iv) on the construction of the Bayesian phylogenetic tree with a precise estimate of the divergence time, (v) on the evaluation of the haplogroup classification using the complete mitogenome *versus* partial mtDNA sequences and (vi) on the estimation of the historical maternal effective population size.

## 2 MATERIALS AND METHODS

### 2.1 Identity and origin of samples

In almost all analyses, our work was based on 809 complete mitogenomes, including five aurochs and seven bison (used only in the data set to calibrate the time clock), taken from GenBank. Of the 797 complete mitogenomes representing modern cattle, we directly sequenced 385 animals sampled mainly in central and South‐east Europe (GenBank accession numbers from MZ901375 to MZ901759). Through data mining, we also obtained 116 mitogenomes, while 296 mitogenomes were from GenBank. In some analyses, we also used an additional 189 D‐loop sequences (from np 16,042 to np 16,276) obtained from ancient carbon‐dated bones. In constructing the large median‐joining network (MJN) based on 247 mitogenomes, we also used 47 recently published mitogenomes that were not included in other analyses. To confirm maternal inheritance of the rare haplogroup found in Austrian Murbodner bull Hugo, we additionally sampled four cows related through the maternal lineage, while all living animals in this maternal lineage were identified from the pedigree data using MaGelLAn 1.0 software (Ristov et al., 2016). A T_3_ mitogenome obtained from 11.5 kyBP ancient bone (Lari et al., 2011), only a few mutations away from the reference sequence, was removed from the analyses because phylogenetic modelling by the algorithm BEAST was rather unstable with this sequence, indicating possible contamination. Detailed information on all mitogenome sequences used in this study, along with their corresponding accession numbers, can be found in Tables S1 and S2.

### 2.2 DNA extraction and library preparation for mitogenome sequencing

The commercially available reagent kit DNeasy Blood & Tissue Kit (Qiagen, Germany) was used to extract DNA from bovine samples (milk, hair roots and tissue) according to the manufacturer's instructions. The experiments reported in this work were in accordance with the ethical guidelines of the national legislation.

Amplification of the whole bovine mitogenome (~16,340 bp) was performed by polymerase chain reaction (PCR) in three overlapping fragments according to a modified protocol (Horsburgh et al., 2013). Primer pairs used are provided in Table S3, and PCR protocols applied are summarized in Table S4. The amplified three mitogenome fragments of each sample were transferred to a tube and purified according to the Wizard^®^ SV gel and PCR Clean‐Up System Protocol (Promega).

Purified long‐range PCR products were diluted to approximately 1 ng/μl and subsequently quantified using the Qubit dsDNA HS Assay (Invitrogen).

PCR products were diluted to yield 0.2 ng/μl per sample, and a total amount of 1 ng per sample was used for library preparation. Libraries were prepared using the Nextera^®^ XT DNA Sample Preparation Kit (Illumina) according to the manufacturer's instructions. The dsDNA was incubated with a preloaded Tn 5 transposase to generate tagged fragments and then amplified with barcoded primers to generate the final library. Amplified libraries were purified using Ampure XP beads (Beckman‐Coulter) and quantified by absorbance measurement using a NanoDrop spectrophotometer (ND‐1100; Thermo). Equal amounts of libraries were pooled, and the final pool was analysed and quantified on a Bioanalyser DNA1000 chip (Agilent). The pooled libraries were sequenced in dual‐indexed 100 bp paired‐end mode on the HiSeq 1500 System (Illumina) at Gene Center, Ludwig Maximilians University (LMU), Munich, Germany.

### 2.3 Primary data analysis

Primary data analysis was performed on the LMU system Galaxy Server using a workflow for demultiplexing, mapping and variant calling. Read pairs were demultiplexed using the dual indexes of Nextera XT Library Preparation Kit (Illumina). Reads were mapped to the mitochondrial sequence of the bovine reference mitogenome (GenBank accession number V00654) using the Burrows–Wheeler Alignment (BWA) tool (Li & Durbin, 2009), duplicates were removed, and variants were called using VarScan (Koboldt et al., 2012). Variants were replaced in the mitochondrial genome fasta file, and mapping and calling were repeated until no further variants could be called and no bases without coverage remained. The resulting mitochondrial genome fasta files were used for further phylogenetic analysis. The mean depth and width of mitogenome coverage was calculated using BBmap (Bushnell, 2014) and is shown in Table S5.

To confirm the results of the next‐generation sequencing (NGS) method described above and the presence of rare haplotypes, all four maternal relatives (Esta, Emma, Ella and Elke) of bull Hugo were additionally sequenced on 780‐bp‐long D‐loop sequences (Achilli et al., 2008) in both directions using the Sanger method on the ABI Prism^®^ 310 Platform Genetic Analyzer (Applied Biosystems).

Whole‐genome sequences were downloaded from online databases and converted to Fastq files (Fastqsanger format) using NCBI Sequence Read Archive (SRA) tools (Leinonen et al., 2010). The Fastq files were mapped to a reference mitogenome (GenBank accession number V00654) using the BWA (Li & Durbin, 2009). The following workflow was set in the Galaxy platform (Afgan et al., 2018): the mapped file obtained with BWA‐MEM was processed with ‘Filter SAM or BAM’ (Galaxy vs. 1.1.29) to keep only the reads mapped to the mitochondrial genome (Li et al., 2009), duplicate molecules were removed with ‘MarkDuplicates’ (Galaxy vs. 2.7.1.1) (Broad Institute), and reads were piled up using MPileup multi‐way pileup of variants (Galaxy vs. 2.1.3) (Li, 2011a, 2011b; Li & Durbin, 2009; Li et al., 2009) and using VarScan (Koboldt et al., 2012) for variant detection (Galaxy vs. 0.1) and LMU in‐house tool Fasta SNP Replacer (Galaxy Tool vs. 1.0.0). Mapping results were reviewed using the Integrative Genomics Viewer software (Robinson et al., 2011). Consensus sequences were subjected to phylogenetic analysis. To confirm the results of the method described above, mitogenome of one animal was optioned both from whole‐genome sequencing data and from long‐range PCR products.

All mitogenomes were aligned using the Clustal Omega program (Sievers et al., 2011) imported into MEGA 7 software (Kumar et al., 2016), whereas partial mitogenome sequences were aligned using the ClustalW program (Kumar et al., 2016). The complete mitogenome data alignment was further divided into functional regions: (i) 13 regions encoding proteins, (ii) two regions encoding ribosomal RNA, (iii) one region encoding the D‐loop and (iv) one region representing encoded 22 tRNAs. Stop codons were removed from the partitions (regions) encoding proteins.

### 2.4 Mitogenome diversity and haplotype classification

Five ancient bovine complete mitogenome sequences were excluded from the diversity data set (797 mitogenomes) because we wanted to estimate contemporary diversity (excluding ancient diversity lost over time), while analyses were performed separately for complete mitogenomes (consensus 16,230 bp) and 17 functional regions defined as described above. Mitogenome diversity was presented by percentage of polymorphic sites to total size (*S*%), number of haplotypes (nH), haplotype diversity (HD) and nucleotide diversity (*π*), all calculated by DnaSP v6 (Rozas et al., 2017) and Arlequin v. 3.5.2.2 software packages (Excoffier & Lischer, 2010). The complete mitogenomes and D‐loop sequences (239 bp; from np 16,042 to np 16,276 plus five indels) were classified using the MitoToolPy application (Peng et al., 2015) and by the Bayesian phylogenetic inference.

To visualize the relationships among P haplotypes, after excluding sites with alignment gaps, we constructed a small MJN (Bandelt et al., 1999) by PopART (Leigh & Bryant, 2015) on a small number of representative mitogenome sequences and five ‘Hugo family’ mitogenomes. We also constructed a large MJN containing 247 mitogenomes (Figure S1) to further elucidate the mutational relationship between haplotypes and haplogroups within the entire phylogenetic network.

### 2.5 Phylogenetic and demography analyses

After confirming the absence of recombination using two algorithms implemented in the HyPhy software (Kosakovsky Pond et al., 2005, 2006a, 2006b), see Table S6, we proceeded with Bayesian phylogenetic inference model testing using the software package BEAST v1.4.3 (Drummond et al., 2006).

Testing of the model and calibration of the tree root was performed on the selected set with 47 mitogenomes: 36 sequences representing modern cattle haplogroups (I, R, P, Q, T_1‐7_), four sequences representing ancient *Bison priscus*, two sequences representing outlying *Bison bison*, and five sequences representing ancient *Bos primigenius primigenius* (aurochs). The program was run with Markov chain Monte Carlo length until the values of the effective sample size (ESS) exceeded 200. Calculations were performed using various substitution models: Generalize times reversible (GTR; Lanave et al., 1984), Hasegawa–Kishino–Yano (HKY; Hasegawa et al., 1985), Jukes–Cantor 1969 (JC69; Jukes & Cantor, 1969), Kimura (K80; Kimura, 1980) and Tamura–Nei (TN93; Tamura & Nei, 1993). The strict molecular clock, relaxed lognormal and exponential molecular clocks were tested (Drummond et al., 2006), and different population size priors were applied: coalescent constant, coalescent exponential and coalescent Bayesian skyline model. The different phylogenetic models were analysed in Tracer v1.6 based on a comparison of Akaike's information criterion values obtained by Markov chain Monte Carlo (AICM, see Raftery et al., 2007). The BEAST analysis was run using priors obtained from the previous analysis for the best‐fitting model: the GTR substitution model, the strict molecular clock and the coalescent Bayesian skyline tree prior (Table S7). To obtain an accurate tree topology, a total of 802 sequences (including five aurochs with information on their estimated time) were imported into BEAUti v1.4.3 (Drummond et al., 2012) and set to a best‐fitting model. The TreeHigh prior was set to 218.9 kyBP (95% HPD 316.6–136.6 kyBP) and the normal distribution with a standard deviation of 52.0 kyBP to be compatible with the calibration tree. The given trees file was compiled in TreeAnnotator v2.4.7 with burn‐in of 20%, while the most recent common ancestor tree was constructed in FigTree v1.4.3 (Drummond et al., 2012).

To estimate the population history, the Bayesian skyline plot (BSP) was calculated in Tracer v1.7.1 software (Rambaut et al., 2018). A D‐loop data set containing modern and calibrated ancient sequences was split into four different data sets representing different haplogroups Q, T_2_, T_3_ and T_1_. Each data set representing an individual haplogroup was run in BEAST v2.5 (Bouckaert et al., 2019) using the GTR substitution model, strict molecular clock and coalescent Bayesian skyline.

## 3 RESULTS

We have conducted a comprehensive, large‐scale complete mitogenome analysis in cattle that has greatly expanded the available mitogenome information by adding 501 new sequences representing 58 additional breeds. Our mitogenome information has largely focused on Europe. In addition, we obtained a large number of samples from the South‐east Europe, as this region is the main agricultural gateway to Europe during Neolithization and has not been well covered in previous analyses (Table S1). Our results are also representative of modern cattle from Africa and the West and East Asia, as mitogenomes from these regions were well represented in our data set but do not cover well indicine cattle from Asia and taurine cattle from South‐East Asia.

### 3.1 Mitogenome diversity and MitoToolPy haplotype classification

**FIGURE 1:**
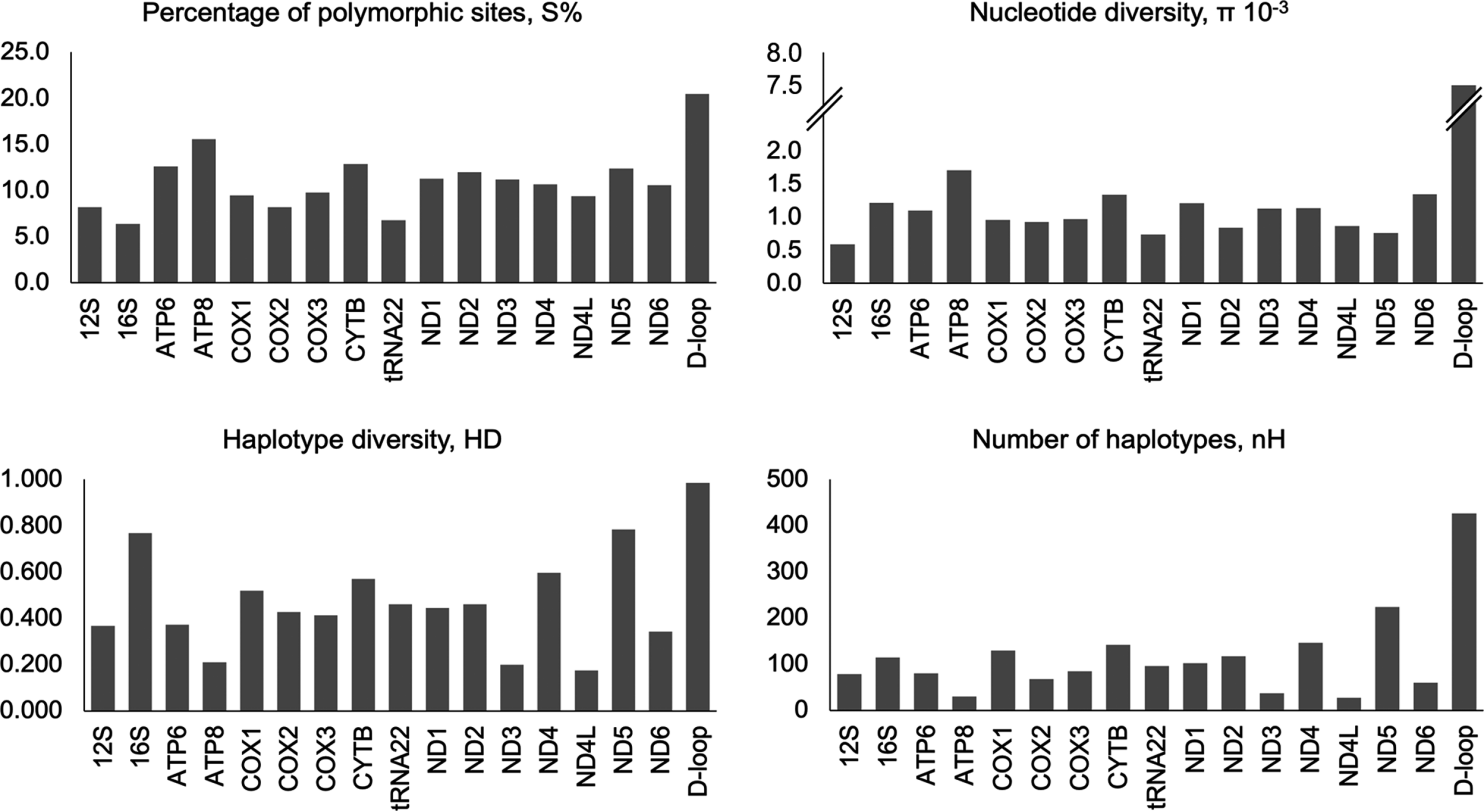
Mitogenome diversity in modern cattle (797 sequences) across different functional regions

Mitogenome diversity (S%, nH, HD and *π*) for different functional regions of 797 modern cattle representing 114 breeds is shown in Figure 1. In total, we found 1725 polymorphic sites (*S*% = 10.6%), including 810 singletons, 853 parsimony‐informative sites and 57 indels. There were also 1563 transitions, 159 transversions and 1678 substitutions, while 701 unique haplotypes were observed with a HD of 0.9995 and a *π* of 0.0015.

Mitogenome diversity varied among the different functional regions (Figure 1), with the highest diversity observed in the D‐loop region (*S*% = 20.5, nH = 426, HD = 0.986 and *π* = 0.0080), followed by ATP8 (*S*% = 15.6), ND5 (nH = 224), ND5 (HD = 0.784) and ATP8 (*π* = 0.0017). In contrast, the lowest diversity was observed in 16S (*S*% = 6.4), ND4L (nH = 27), ATP6 (HD = 0.199) and 12S (*π* = 0.0006).

**FIGURE 2:**
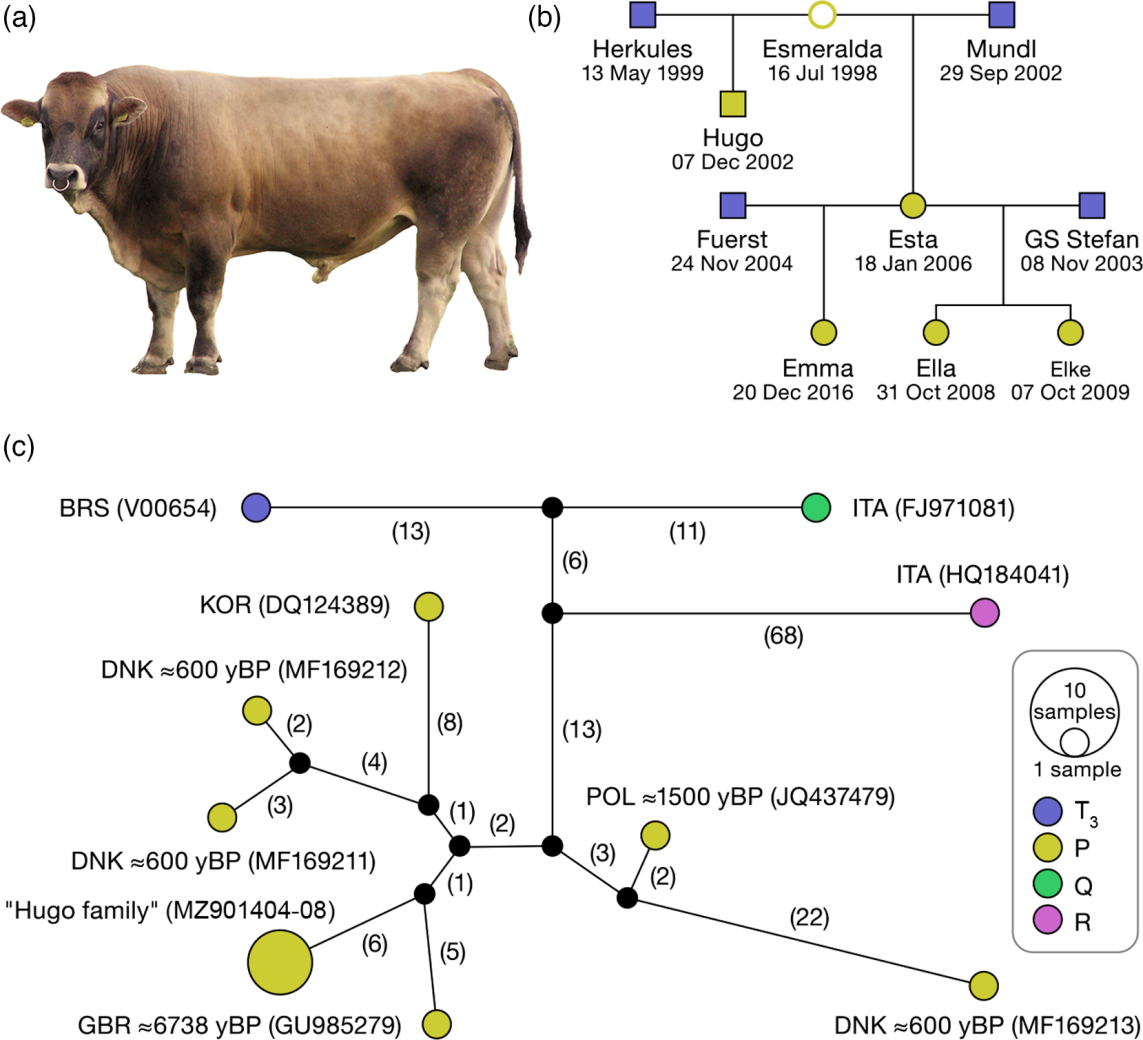
Presentation of the Murbodner cattle “Hugo family” with surviving aurochs P haplogroup mitochondria. (a) Photo of the bull named Hugo first identified with P haplotype mitogenome. (b) Reduced family tree of the members of the “Hugo family”, in which the positions of the individuals sequenced with haplotype P are marked as full yellow squares or circles, (c) Median‐joining network (mitogenome length of 16,344 bps) representing the relationship between P and few other haplogroups (R, Q and T3). Abbreviations: BRS, Bovine Reference Sequence (T3); DNK, Denmark; GBR, Great Britain; ITA, Italy; KOR, Korea; POL, Poland are associated with declared origin of sequences

Mitogenomes of modern cattle (797) were classified into I (10), R (10), P‐ (6), Q (22), T_5_ (10), T_2_ (42), T_3_ (528), T_1_ (154), T_4_ (11), T_6_ (3) and T_7_ (1) haplogroups using the MitoToolPy algorithm. In Austrian Murbodner cattle originating from the Alpine Murtal valley, we found the aurochs‐specific haplotype P in a bull named Hugo (Figure 2a). The presence of haplotype P in Murbodner cattle is the first evidence for the survival of mitochondria of ancient northern and central European aurochs, genotyped as haplogroup P in many ancient aurochs’ bones (Ajmone‐Marsan et al., 2010; McHugo et al., 2019), in modern European cattle.

This evidence, the identification of the P haplotype, was confirmed by mitogenome (NGS) and D‐loop Sanger sequencing of four cows (Esta, Ella, Emma and Elke) related to Hugo (Figure 2b). Pedigree analysis, the identification of pedigree members within the same maternal lineages, revealed that 99 animals born between 1991 and 2015 belonged to the maternal P‐lineage (Figure S2). Until recently, only several ancient or historical P haplotypes were documented in GenBank: British aurochs Pre‐Neolithic (6.7 kyBP; GU985279); historical Polish aurochs (1.5 kyBP; JQ437479); three historical ‘Danish’ aurochs (MF169211, MF169212 and MF169213; all 0.6 kyBP); and one modern cattle (Korean beef cattle sample DQ124389). After all our mitogenome analyses were completed, 14 individuals with haplotypes several mutations away from the Korean sequence DQ124389 were found in the Japanese Shorthorn (Mannen et al., 2020) and one in Chinese (Yanbian) individual (Xia et al., 2021). In the MJN (Figure 2c and Figure S1), the P haplotype found in Murbodner cattle (MZ901404‐08) was closest (11 mutations) to the reference Pre‐Neolithic aurochs (GU985279).

### 3.2 Bayesian phylogenetic inference and haplotype classification

**FIGURE 3:**
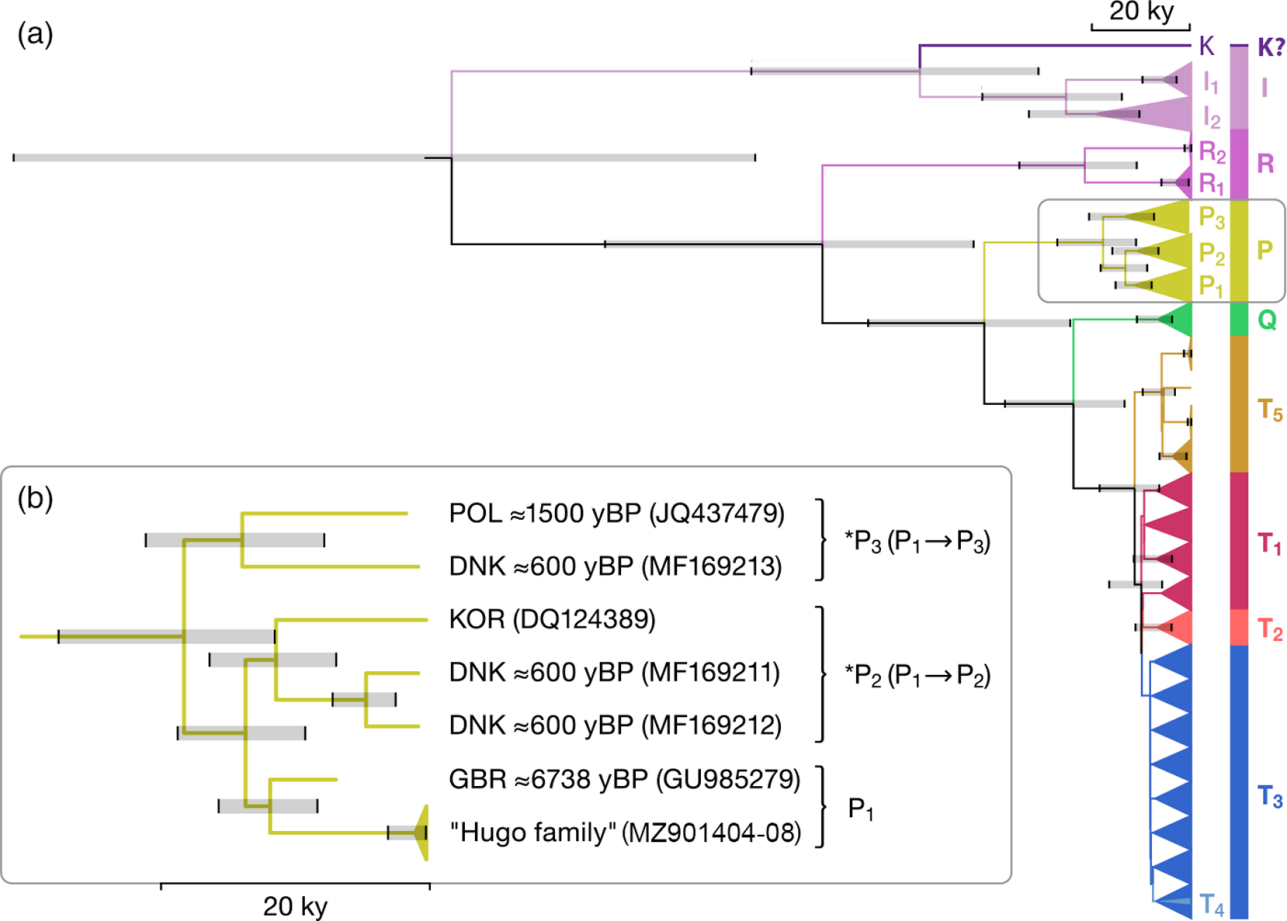
The Bayesian phylogenetic tree performed on 802 complete mitogenome cattle sequences. (a) Complete phylogenetic tree (b) Partial phylogenetic tree representing P haplotypes (DNK, Denmark; GBR, Great Britain; KOR, Korea; POL, Poland)

Bayesian phylogenetic tree inference performed on 802 mitogenomes (modern cattle with five chronologically distant aurochs) is shown in Figure 3, while the posterior probabilities with estimated divergence time of major haplogroups (12 nodes), calculated from the calibration tree (Figure S3), are provided in Table 1. According to the results, the GTR substitution model and strict molecular clock with Bayesian skyline tree prior had the lowest AICM value, while the tree root for genus Bos was 218.9 kyBP (Figure S1). Diversification between genus Bos and Bison occurred 965.3 kyBP (95% HPD 1348.0–600.4 kyBP, posterior probability = 1). A more detailed phylogenetic tree representing the phylogenetic relationship of all individual mitogenomes is presented as a zoomable user interface (Zoom Figure S4 and its digitally formatted file, Figure SD1: Data S1, readable by the FigTree graphical viewer).

**TABLE 1:** Haplogroup divergence time (DT) in kiloyears before present (kyBP) according to the Bayesian Markov chain Monte Carlo calibration tree with corresponding node numbers (Figure S3)

Haplogroup divergence time	Median	95% HPD^a^	Posterior probability
Node 1: I–[R–P–Q–T]	218.9	316.6–136.6	1.00
Node 2: I_1_–I_2_	21.2	46.2–10.3	1.00
Node 3: R–[P–Q–T]	102.7	149.9–63.7	1.00
Node 4: R_1_–R_2_	28.5	46.2–14.6	1.00
Node 5: P–[Q–T]	56.8	83.9–35.0	1.00
Node 6: P_3_–[P_1_–P_2_]	21.1	31.4–13.2	1.00
Node 7: P_1_–P_2_	15.9	23.6–10.3	0.98
Node 8: Q–T	33.5	51.0–20.4	1.00
Node 9: Q_1_–Q_2_	11.8	18.2–6.4	1.00
Node 10: T_5_–[T_1_–T_2_–T_3_–T_4_]	18.2	27.5–11.4	1.00
Node 11: T_2_–[T_1_–T_3_–T_4_]	16.3	24.0–10.2	0.95
Node 12: T_1_–T_3_	14.7	21.2–8.8	0.60

^a^ The highest posterior density pointing to 95% posterior credible intervals.

The divergence of haplogroups T_5_ and T_2_ from T_1_ to T_4_ was supported, whereas the divergence between haplogroups T_1_ and T_3_ was not supported by the derived Bayesian posterior probabilities (node 12 in Table 1). Thus, as a consequence of the low divergence, the assignment between T_3_ and T_1_ could be misguided, especially for the incomplete and uninformative sequences obtained from ancient samples. However, the geographically specific distribution of T_1_ in a large number of African cattle breeds is a solid argument to consider T_1_ as a separate haplogroup, while the process of its further diversification is unstoppable.

Divergence of haplogroup T_4_ from T_3_ was supported only on the hierarchically lower branch (not supported in the calibration analysis with 47 sequences), which diverged with a posterior probability of 0.95, and was positioned after several branches with very low posterior probabilities (Figure SD1: Data S1). At the same time, haplogroup T_4_ was clustered with a posterior probability of 1, along with individuals sampled in Korea and placed within the T_4_ cluster in MJN (Figure S1). The presence of a Middle Albanian Buša individual within the T_4_ mitogenome was therefore surprising, as T_4_ is considered an Asian‐specific haplotype that is most common in modern Japanese and Korean cattle (Figures S1 and S4). Recently, T_4_ mitogenomes were identified from ancient bones in China, which are about 3.9 kyBP old (Xia et al., 2021). In conclusion, given the geographic specificity, the frequent use in the literature and the logic followed in the case of T_1_, the recognition of T_4_ as a separate haplogroup is an acceptable decision.

On the contrary, we did not find a strong argument to support T_6_ and T_7_ as separate haplogroups, since their occurrence is extremely rare, and it is unlikely (low posterior probability) that they branch from T_3_ (Figure SD1: Data S1). Three sequences (AY676867, AY676870 and SRS629688) from Angus cattle classified as T_6_ were very similar or identical to the T_6_ mitogenome found in Xia et al. (2019) if we neglect the sites with alignment gaps (Figure S1). Interestingly, haplotypes T_6_ and T_7_ were located in a separate branch between haplogroups T_3_ and T_2_ along with seven haplotypes (JN817319, JN817306 and EU177841 from Italy; MZ901433, MZ901461 and MZ901447 from Croatia; and KT184470 from Egypt) classified as T_1_ by the MitoToolPy algorithm. For example, haplotype T_7_, previously found in Cabannina cattle in Italy (EU177840), was not reproduced by the diagnostic mutations (Achilli et al., 2008), where it was classified as T1′2′3 haplotype, nor by the BEAST algorithm, where the posterior probability was 0.4 (for more details, see Figure SD1: Data S1). Certainly, more information, sequences from ancient DNA and work are needed to clarify the exact classification of this entire cluster.

In addition to other known haplogroups, Bayesian inference revealed P_1_, P_2_ and P_3_ as separate haplogroups, with the estimated divergence time between P_1_–P_2_ and P_3_ ranging from 31.3 to 13.2 kyBP, and between P_1_ and P_2_ ranging from 23.6 to 10.3 kyBP (Table 1). Thus, Murbodner cattle together with British Pre‐Neolithic aurochs (GU985279) were assigned to P_1_, whereas Korean cattle (DQ124389) and two Scandinavian aurochs (MF169211 and MF169212) sequenced from 0.6‐kyBP‐old horns (Bro‐Jørgensen et al., 2018) were assigned to haplogroup P_2_ (Table 1 and Figure 3b). The Wild Polish aurochs (JQ437479), sequenced from 1.5‐kyBP‐old bone (Zeyland et al., 2013), and the Scandinavian aurochs (MF169213), sequenced from 0.6‐kyBP‐old horn (Bro‐Jørgensen et al., 2018), were assigned to haplogroup P_3_ (Figure 3b). Both estimated divergence times, between P_1_‐P_2_ and P_3_ and between P_1_ and P_2_, occurred before the domestication of cattle, as it is reasonable to assume that by 10.3 kyBP (lower 95% HPD limit of estimated divergence between P_1_ and P_2_) P aurochs were widespread, far from Fertile Crescent (Table 1).

A sequence (DQ124403), found in Korean Holstein cattle, diverged from haplogroup I and may be evidence of a new haplogroup ‘K’ descended from *Bos primigenius namadicus* (Figure 3a and Figure S4). Because we did not have access to sequencing quality parameters or access to their biological relatives (sequence taken from GenBank), we made no additional comments or speculations regarding this sequence.

The Bayesian phylogenetic tree obtained by the BEAST algorithms on the complete mitogenomes, even considering the ‘explanatory supported’ T_4_ or unsupported T_6_, and T_7_ haplogroups, was mostly consistent with that obtained by the MitoToolPy algorithms. However, we observed situations where the MitoToolPy classification was not correct, particularly in the classification of T_1_ and T_3_ (Table S1).

Even lower agreement was observed when classifications were based on partial mitogenome sequences, such as the D‐loop (Table S1 and Figure S5A). The most drastic example is the misclassification of the historical Scandinavian aurochs (MF169213 with haplotype P, which was incorrectly classified as T_3_ based on D‐loop information; Table S1 and Figure S3A). A very similar problem was observed for a number of T_5_ mitogenomes that could not be recognized at all using only the D‐loop information (Table S1 and Figure S5A). Thus, reliable haplogroup classification requires information on the complete mitogenome. Following this logic, haplogroup E, classified on the basis of the partial mitogenome from the 5.8‐kyBP‐old bone (known as EIL4) from the German Early Neolithic site of Eilsleben (Edwards et al., 2007), needs to be confirmed using the complete mitogenome information, which presents another challenge for future ancient DNA analyses.

### 3.3 Bayesian demographic inference

**FIGURE 4:**
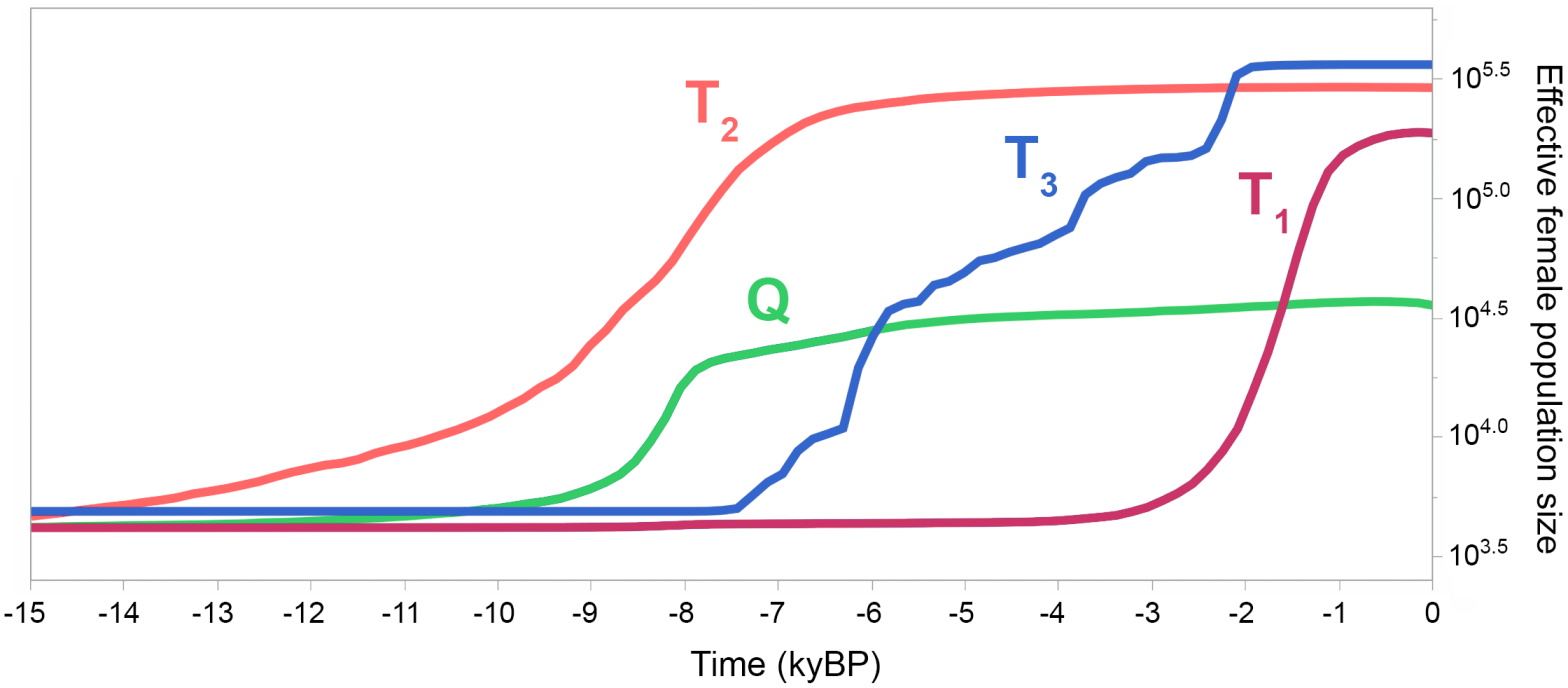
Bayesian skyline plot showing the trend in effective female population size over the last 15 thousand years for haplogroups Q, T2, T3 and T1, inferred from D‐loop sequence information (from np 16,042 to np 16,276)

A BSP was performed for each haplogroup (Q, T_2_, T_3_ and T_1_) on D‐loop, from np 16,042 to np 16,276, sequence information (Figure 4). The analysis was done on 999 sequences, our mitogenomes restricted to partial D‐loop, extended by additional 189 D‐loop sequences obtained from the carbon‐dated bones as number of high‐quality complete ancient mitogenomes is very low. The inferred trend in effective female population size (*N*_ef_) for each of the four haplogroups during a period of the last 15 thousand years is shown in Figure 4 (estimates with 95% posterior credibility intervals are shown in Figure S6). The growth of *N*_ef_ in T_2_ started before domestication and accelerates from the temperature changes in Bølling–Allerød (warm and humid interstadial period occurring in the last stages of the last ice age, from 14.7 to 12.7 kyBP). However, the steepest growth of T_2_ (10.0 to 7.5 kyBP) and the sudden growth of Q (from 10.0 to 8.0 kyBP) almost overlap with the timing of archaeological evidence pointing to the beginnings of cattle domestication (Helmer et al., 2005). For both T_2_ and Q, estimated *N*_ef_ growth was slowed to ~8.0–7.0 kyBP. Around the same time, we observed a sudden and steep *N*_ef_ growth in T_3_ that occurred around ~7.5 kyBP and then slowed down to ~2.5 kyBP. In Africa‐specific haplogroup T_1_, the increase in *N*_ef_ growth from ~3.0 to 1.0 kyBP overlaps with the timing of the migration routes of Bantu‐speaking farmers and Cushitic‐ and Nilotic‐speaking pastoralists (Crowther et al., 2018), while it slows down with the assumed arrival date of the Zebu in Africa (Kim et al., 2020).

Alternatively, the earliest undisputed archaeological evidence for zebu cattle in Africa comes from rock carvings of humpbacked cattle from the Horn of Africa, dated to about 1.5 kyBP, which would roughly correspond to the inflection point of the observed T_1_ growth function (Gifford‐Gonzalez & Hanotte, 2011). Under this assumption and the fact that all African cattle carry the T_1_ haplotype, it is also possible that T_1_ growth was promoted by zebu introgression as a result of enhanced adaptation of the paternal zebu and maternal African taurine crosses (which possess T_1_ haplotypes) to the local environment (high temperatures, prolonged droughts and vector‐borne diseases), which has been discussed in a number of studies related to modern cattle–zebu crosses (Kim et al., 2020; Mbole‐Kariuki et al., 2014; Mwai et al., 2015).

## 4 DISCUSSION

### 4.1 Mitogenome diversity

We estimated complete and partial mitogenome diversity in functionally distinct regions and have observed that the estimated complete mitogenome nucleotide diversity (0.0015) is comparable to the genome‐wide nucleotide diversity of 0.0010 to 0.0020 observed in other taurine breeds (Chen et al., 2018; Kim et al., 2017; Weldenegodguad et al., 2019). As expected, the D‐loop was the most diverse functional region, although notable polymorphism was observed in other functional regions. At the same time, the nucleotide diversity of the bovine mitogenome observed in this study is relatively low but within the range of values calculated for other mammals, where *π* ranges from 0.106 in the southern pocket gopher to 0.0004 in the grey‐browed mouse lemur (Nabholz et al., 2008).

It is worth noting that the presence of nuclear DNA sequences of historical mitochondrial origin or NUMTs (pseudogenes derived from partial mitogenome fragments historically integrated into the nuclear genome of eukaryotes by a horizontal transfer mechanism) could have an impact on the diversity estimates presented (Maude et al., 2019; Schiavo et al., 2020; Tramontin Grau et al., 2020). However, the possibility of such a bias was significantly reduced. Thus, the large number of sequences (385) was performed by the three long‐range PCRs, where the chance of narrowing down the NUMTs is almost eliminated. When controlled by other sequencing methods (Sanger sequencing), we did not detect any divergence (except for a single slip, which was corrected in further analyses). For the 116 sequences obtained by data mining, we cannot exclude the possibility of misentries in a few places caused by overlap of NUMTs and heteroplasmy calls. Such cases might have led to a slight overestimation of the number of observed mitogenomes. However, this effect and the slight overestimation of nuclear diversity could also have been caused by other sequencing errors or tissue‐specific mutations that are not inherited (very low probability). Our visual inspection of potentially suspicious ‘chains of consecutive heteroplasmy calls’ revealed no such case. Moreover, our recombination analysis found no site that would indicate the presence of recombination, a phenomenon characteristic of the nuclear genome. We provided information on the sequences obtained by data mining (Table S1) and sequence quality to inform readers of potentially suspicious sequences (Table S5).

### 4.2 Complexity of domestication from mitogenome perspective

**FIGURE 5:**
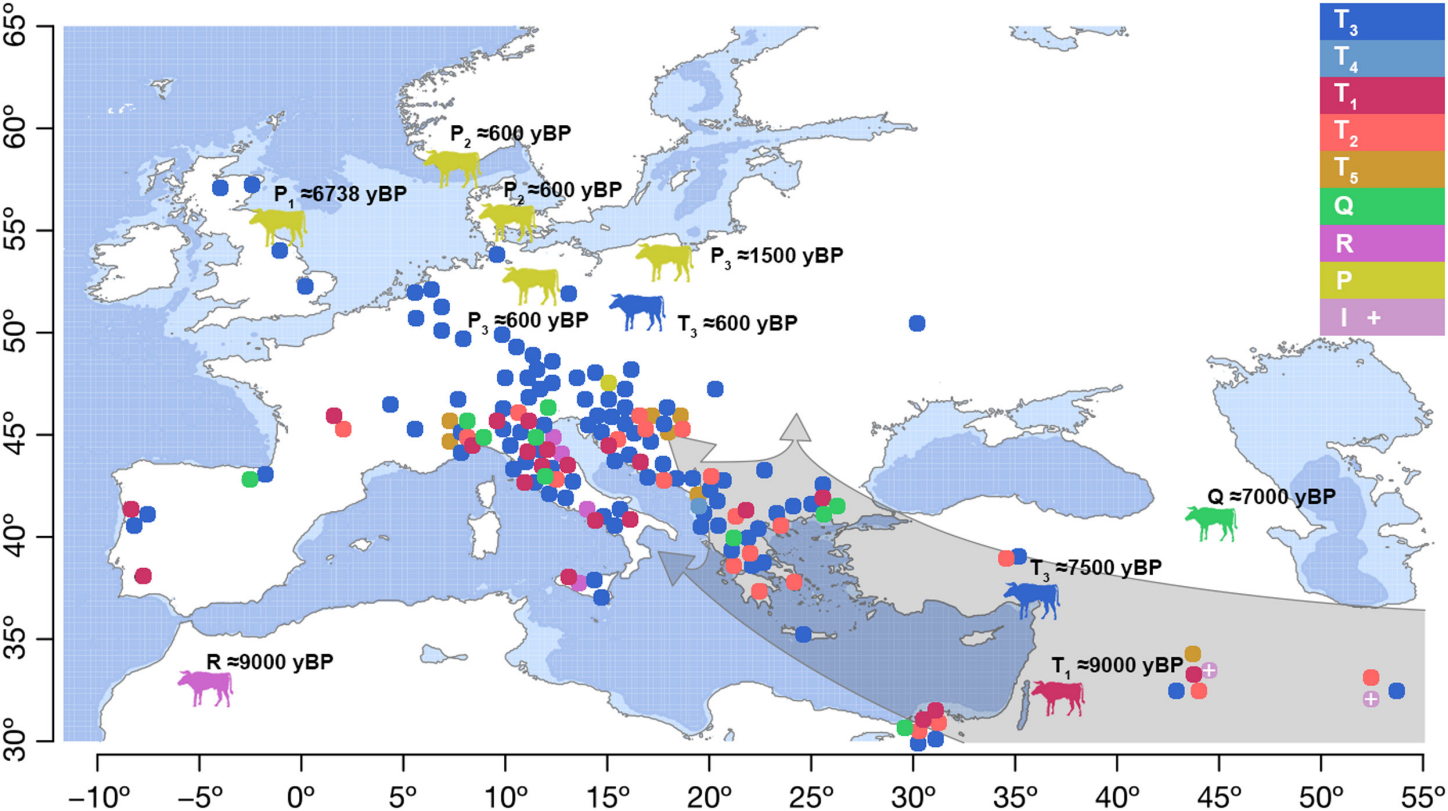
Illustration of the geographical distribution of the analysed mitogenome sequences of European modern cattle breeds with haplogroup assignment according to Bayesian phylogenetic analysis. Aurochs (silhouettes) with sequenced mitogenomes are shown with estimated time of death in years before present (kyBP). Grey shaded arrows indicate agricultural dispersal, while light blue areas show historical sections where grazing may have occurred (sea level 100 m)

With the exception of the most common T_3_, a large number of different haplogroups (Q, T_5_, T_2_, T_1_ and T_4_) have been identified in South‐east European breeds (Figure 5), which in Europe is only comparable to the variation found in neighbouring Italian breeds. The variation found in South‐east European cattle can be attributed to Neolithic migrations, as haplogroups T_3_, T_2_ and Q were already present in Serbia and Greece ~7.0 kyBP (Verdugo et al., 2019). Boskarin and Slavonian Syrmian Podolian cattle, two representatives of the two Podolian clusters (Senczuk et al., 2021), were predominantly assigned to the T_3_ haplogroup, with the exception of three T_5_ haplotypes in the latter breed. This result is consistent with the conclusions of Di Lorenzo et al. (2018), who found no mitogenome differences between Podolian cattle from Central Europe and the Balkans and Podolian cattle from Italy. Also, the autosomal diversity of Podolian and neighbouring shorthorn breeds does not support a clear clustering into breed groups (e.g. Papachristou et al., 2020), but rather agrees with the genetic similarity of geographic neighbours, that is the ‘isolation‐by‐distance’ model. However, two samples from Podolian cattle, one from the Boskarin breed and the other from the Calvana breed, were positioned within the ‘central’ T_1_‐T_3_ haplotype of the large MJN (Figure S1), along with three ancient samples derived from 7.6‐ to 8‐kyBP‐old bones (found in Greece and Turkey; see Verdugo et al., 2019), providing further evidence for their association with Neolithic migrations.

The appearance of T_1_ in South and South‐east European breeds was not surprising, as the migratory flow from African to southern European cattle has already been estimated at the nuclear level (Decker et al., 2014; Senczuk et al., 2021; Upadhyay et al., 2019) or is evident from the presence of R and T_1_ haplotypes in Italian breeds (Achilli et al., 2008, 2009; Bonfiglio et al., 2010). It is also possible that the observed T_1_ is the result of direct migrations during Bronze Age or later, as 11 (48%) T_1_ haplotypes have been found in ancient bone samples from the southern Levant (Verdugo et al., 2019).

The occurrence of six T_5_ haplotypes in cattle from Croatia and Albania was intriguing, as only four complete mitogenomes with T_5_ assigned to two Italian and two Iraqi cattle were previously reported (Achilli et al., 2008). Given the divergence time of T_5_ from T_1_–T_2_–T_3_–T_4_ (Table 1) and the current geographic distribution of T_5_ in modern cattle (east and west of the centre of domestication), it is highly likely that haplogroup T_5_ was sampled during the domestication of the Anatolian aurochs. Note that T_5_ may have been missed in previous DNA analyses, especially in ancient bone samples, as the D‐loop information is usually insufficient to identify T_5_. However, the alternative hypothesis of a local origin from the Great Adriatic Plain (on sea‐level dynamics; see Dean et al., 2020) can be tested by analysing aurochs bones found at several interesting archaeological sites in Eastern Adriatic (Lenardić Much et al., 2018; Miracle, 2007).

The appearance of the T_4_ haplotype in the Middle Albanian Buša was also surprising, and its origin requires further clarification.

While the variation of diverse haplogroups was low in Central European cattle, we found a large maternal lineage of 99 individuals in the Austrian Murbodner cattle, originating from the Alpine valley Murtal, whose mitogenome is assigned to the P haplogroup specific for the large‐boned ancient European aurochs.

Murbodner P maternal lineage was assigned to the P_1_ haplogroup, together with the mitogenome found in 7.0‐kyBP‐old ancient bones of the Pre‐Neolithic British aurochs (Edwards et al., 2010; Park et al., 2015). This is the most convincing evidence for the sporadic taming of now extinct European wild female aurochs and the embedding of their offspring into domesticated European cattle. Unfortunately, we were unable to estimate the exact time frame, location or context of interbreeding. The taming of wild female aurochs and the inclusion of their offspring in the domesticated cattle stock are complemented by archaeozoological evidence (Schibler et al., 2014), as ancient small cattle bones found in Switzerland (5.0 kyBP), also associated with the Alps, were identified as P. Current explanations for the rare occurrence of P haplotypes in modern cattle remain speculative, for example a selective disadvantage of P mitochondria in warmer environments or a mitonuclear incompatibility (Sharbrough et al., 2017; Zaidi & Makova, 2019). The simplest and most obvious explanation, however, would be that the European and Anatolian aurochs were two well‐differentiated populations before and during the Holocene. Anatolian Neolithic Farmers selected local aurochs for domestication and tried not to lose the hard‐won domestication traits during expansion in Europe. Only in emergencies is there an increased willingness to accept a much greater effort to integrate wild or semi‐wild animals into domesticated populations. A more frequent occurrence of such emergencies is conceivable in fragmented Alpine environments.

The presence of R haplotypes in modern Italian breeds (Achilli et al., 2008) is additional evidence for the interbreeding of domesticated cattle and unidentified female aurochs with R mitogenomes, recently confirmed by the identification of R haplotypes in bones of a 9.0‐kyBP‐old aurochs, probably *Bos primigenius africanus*, from Morocco (Verdugo et al., 2019). However, it is difficult to speculate on the geographic distribution of R‐haplogroup aurochs on the basis of a single sample, and more information is needed to better understand the link between modern Italian breeds and R‐haplogroup aurochs.

The occurrence of P_2_ haplotypes in modern cattle in North‐east Asia and Japan assigned to haplogroup P_2_ (DQ124389 and related sequences) also provides little evidence for the pattern of cattle domestication during Neolithization in Europe. With the exception of Japanese Shorthorn, haplogroup P_2_ (P_1a_ in Mannen et al., 2020) is extremely rare and has only been found in two ~0.6‐kyBP‐old aurochs’ samples (MF169211 and MF169212) in northern Europe (Figure S1). Interestingly, P_2_ is very similar to haplogroup T_3_ at the amino acid level (Figure S5B).

### 4.3 Bayesian demographic inference

The estimated demographic patterns of *N_ef_* obtained by the BSP for Q, T_2_, T_3_ and T_1_ are rather surprising. The observed trend patterns were ‘suspiciously’ consistent with archaeological evidence about the domestication process and with the growth of human populations. According to the observed trend pattern, the domestication of cattle overlaps with the accelerated rise of the T_2_ haplogroup and was followed by the sudden rise of the Q haplogroup between ~10.0 and ~8.0 kyBP. This is consistent with the known archaeological evidence on the location and timing of beginnings of domestication at Fertile Crescent (~10.5 kyBP) and with the geographic distribution of the T_2_ and Q haplogroups. Chronologically, this corresponds to general warming trend since the beginning of the Holocene (Isarin & Renssen, 1999). Interestingly, at the time when T_2_ and Q reached their upper growth limit, the sudden growth of T_3_ was initiated (by ~7.5 kyBP). We speculate that at the time T_3_, cattle located at South‐east Europe (see Verdugo et al., 2019), or what is colloquially referred to as the Balkans, began their growth and expanded further westwards. Chronologically, the growth of the T_3_ haplogroup is consistent with the estimated growth in effective female population size observed in Europeans with haplogroups of Near Eastern origin from the Holocene during the agricultural transition (Gignoux et al., 2011) and with the introduction of agriculture into Central Europe from the Balkans (Pinhasi et al., 2005). In addition, the observed growth of T_1_ cattle overlaps chronologically, albeit with some divergence, with human female population expansion in Africa (Gignoux et al., 2011), as well as with southward cattle herding spread in Africa (Crowther et al., 2018; Felius et al., 2014; Payne & Hodges, 1997). It is important to note that our T_1_ *N*_ef_ trend is also based on T_1_ haplotypes that were not sampled in Africa and therefore reflect growth of T_1_ cattle outside Africa, which cannot be separated. Therefore, our discussion is based on the assumption that T_1_ growth was characteristic of cattle in Africa, while non‐African samples provide information on the flat T_1_ trend that preceded the observed growth.

Overall, the Bayesian 95% posterior credibility intervals for all *N*_ef_ trends presented are wide (see Figure S6), and therefore, despite the ‘perfect’ agreement with historical demography and archaeological evidence, all our comments on the estimated *N*_ef_ trends should be considered as hypotheses that need to be confirmed with additional arguments or/and evidence.

### 4.4 The impact of aurochs on the modern cattle diversity and adaptation

The initial evidence provided on ancient partial mitogenomes was misleading and did not support the influence of the local aurochs on the evolution and diversity of modern cattle (McHugo et al., 2019), while the presence of a divergent R mitogenome found in Italian cattle (Achilli et al., 2008) was obscured by the fact that R was not observed in any ancient aurochs until recently (Verdugo et al., 2019). Encouragingly, the presence of gene flow from aurochs to modern cattle has been estimated from the whole‐genome sequence of ancient aurochs (Park et al., 2015; Verdugo et al., 2019).

Our study provides unequivocal evidence, albeit based on a ‘single locus’ (mitogenome), for interbreeding of wild female aurochs with domesticated cattle that has been lacking for several decades, complements the findings of Park et al. (2015) and Verdugo et al. (2019), and further supports the complexity of cattle domestication, where the diversity of modern cattle reflects local adaptations of ancient aurochs populations. Thus, cattle join other domesticated species such as pigs, sheep and horses, where evidence supports gradual domestication with continuous interbreeding between domesticated and wild forms (Frantz et al., 2020; Larson & Burger, 2013). Genetic introgression within the *Bos* species complex appears to be a common mode of adaptation to substantially different environments (Wu et al., 2018), as introgression has been observed between taurine cattle and yak (*Bos grunniens*) (Medugorac et al., 2017; Xia et al., 2021) and between indicine cattle and banteng (*Bos javanicus*) (Chen et al., 2018). Furthermore, Medugorac et al. (2017) link periods of intense introgression to times of crisis that forced yak herders to breed all available females to restore their herds, including otherwise unwanted females obtained through backcrossing. Long‐term survival and sustainable development depend on a diversity of variants and options that can be combined to adapt to new environments. Genetic diversity represents a unique ‘information bank’ that ultimately determines the potential for life to evolve with the abiotic component of the ecosystem in the most resilient manner possible (e.g. Steffen et al., 2015). Adaptation to a changed or new environment occurs much more rapidly when an appropriate functional solution already exists in a related sympatric population. Therefore, adaptive introgression of such functional traits is a proven evolutionary solution for sustainable adaptation and long‐term survival. Given the complex and polygenic architecture of adaptation and domestication traits (Carneiro et al., 2014; Larson & Burger, 2013; Medugorac et al., 2017), we propose adaptive introgression as an important evolutionary force during the expansion of domesticated cattle into European environments.

Domestication and subsequent expansion exposed domestic animals to many new environmental conditions, including greater temperature extremes, different pathogens and nutrients. Around the time of expansion, a presumably large aurochs population existed in Europe (Wright & Viner‐Daniels, 2015). Rare intentional or unintentional hybridization thus increased the ability of domestic cattle to survive and adapt to new environmental conditions by incorporating genetic material from local species. Adaptive introgression is more likely to occur through the birth of F_1_ hybrids in the domestic cattle population, that is male‐mediated introgression from European aurochs into domesticated cattle (Götherström et al., 2005; Pérez‐Pardal et al., 2010) or male‐mediated introgression of indicine cattle into Middle Eastern (Verdugo et al., 2019) or African taurine cattle (Kim et al., 2020). On the contrary, female‐mediated adaptive introgression is associated with the maintenance of pure wild animals and could therefore be much more difficult. We therefore suspect that the extremely rare integration of wild female calves into domestic stock occurs only in emergencies. Of course, we cannot exclude the speculation that the introgression of the aurochs was necessary to lower the degree of inbreeding of the existing domesticated cattle or was simply a consequence of the exploitation of heterosis effects. For example, crossbreeding of yak with cattle has been traditionally practised in Asia for a long time (Zhang, 2000).

The finding of surviving aurochs’ mitochondria not only provides indisputable evidence for gene flow between European wild aurochs and domesticated cattle but also enables the protection of already endangered maternal cattle diversity. No less importantly, Murbodner cattle with the P_1_ haplotype are likely to be of interest for ongoing aurochs de‐extinction programmes (Sinding & Gilbert, 2016; Stokstad, 2015) and for research on the expression of mitonuclear interacting gene complexes.

## CONFLICT OF INTEREST

None declared.

## Supporting information

Supplementary Materials

Data S1

## Data Availability

Mitochondrial sequences are deposited in GenBank (accession numbers from MZ901375 to MZ901759). Fasta files used in the analyses are available upon request.
