# Supplementary material for: Large‐scale mitogenome sequencing reveals consecutive expansions of domestic taurine cattle and supports sporadic aurochs introgression
Source: Evol Appl. 2021 Nov 27;15(4):663–78. doi: 10.1111/eva.13315 (PMC9046920; doi:10.1111/eva.13315)
Supplement: Supplementary file 1 — Supplementary Materials [file EVA-15-663-s001.pdf]

Table S1. A detailed description of the analyzed mitogenome sequences.

No.	Fasta name	Acc. no.	SP	Breed	ORG	La-Y/Lo-X	Source	MDCT	MTP M/D	Beast M/D	Author
1	ALB_BuAl_001_T3r_2	MZ901685	BT	Middle-Albanian Buša	ALB	40.93/19.83	T-S	MDC-	T3r/T3	T3/T3	T-S
2	ALB_BuAl_002_T3d_2	MZ901686	BT	Middle-Albanian Buša	ALB	40.93/19.83	T-S	MDC-	T3d/T3	T3/T3	T-S
3	ALB_BuDb_001_T3h_2	MZ901665	BT	Dibra Buša	ALB	41.60/20.25	T-S	MDC-	T3h/T3	T3/T3	T-S
4	ALB_BuDb_002_T3r_2	MZ901666	BT	Dibra Buša	ALB	41.60/20.25	T-S	MDC-	T3r/T3	T3/T3	T-S
5	ALB_BuDb_003_T3r_2	MZ901667	BT	Dibra Buša	ALB	41.60/20.25	T-S	MDC-	T3r/T3	T3/T3	T-S
6	ALB_BuDb_004_T3x_2	MZ901668	BT	Dibra Buša	ALB	41.60/20.25	T-S	MDC-	T3/T3	T3/T3	T-S
7	ALB_BuLe_001_T3r_2	MZ901695	BT	Lekbibaj Buša	ALB	42.33/19.95	T-S	MDC-	T3r/T3	T3/T3	T-S
8	ALB_BuLe_002_T3r_2	MZ901696	BT	Lekbibaj Buša	ALB	42.33/19.95	T-S	MDC-	T3r/T3	T3/T3	T-S
9	ALB_BuLe_003_T3s_2	MZ901697	BT	Lekbibaj Buša	ALB	42.33/19.95	T-S	MDC-	T3s/T3	T3/T3	T-S
10	ALB_BuLe_004_T3n_2	MZ901698	BT	Lekbibaj Buša	ALB	42.33/19.95	T-S	MDC-	T3n/T3n	T3/T3	T-S
11	ALB_BuSk_001_T5x_2	MZ901740	BT	Skodra Buša	ALB	41.90/19.40	T-S	MDC-	T5/QT	T5/Q	T-S
12	ALB_BuSk_002_T3x_2	MZ901741	BT	Skodra Buša	ALB	41.90/19.40	T-S	MDC-	T3/T3	T3/T3	T-S
13	ALB_BuSk_003_T3x_2	MZ901742	BT	Skodra Buša	ALB	41.90/19.40	T-S	MDC-	T3/T3	T3/T3	T-S
14	ALB_BuSk_004_T5x_2	MZ901743	BT	Skodra Buša	ALB	41.90/19.40	T-S	MDC-	T5/QT	T5/Q	T-S
15	ALB_Pres_001_T1b_2	MZ901716	BT	Prespa Cattle	ALB	40.85/20.95	T-S	MDC-	T1b/T1b	T3/T3	T-S
16	ALB_Pres_002_T3r_2	MZ901717	BT	Prespa Cattle	ALB	40.85/20.95	T-S	MDC-	T3r/T3	T3/T3	T-S
17	ALB_Pres_003_T3g_2	MZ901718	BT	Prespa Cattle	ALB	40.85/20.95	T-S	MDC-	T3g/T3	T3/T3	T-S
18	ALB_Pres_004_T3r_2	MZ901719	BT	Prespa Cattle	ALB	40.85/20.95	T-S	MDC-	T3r/T3	T3/T3	T-S
19	ALB_Tief_001_T3r_2	MZ901379	BT	Middle-Albanian Buša	ALB	40.88/19.55	T-S	MDC-	T3r/T3i	T3/T3	T-S
20	ALB_Tief_002_T4x_2	MZ901380	BT	Middle-Albanian Buša	ALB	40.88/19.55	T-S	MDC-	T4/T3	T4/T3	T-S
21	AUT_AtBr_001_T3x_2	MZ901410	BT	Original Austrian Brown	AUT	47.23/11.50	T-S	MDC-	T3/T3	T3/T3	T-S
22	AUT_AtBr_002_T3r_2	MZ901411	BT	Original Austrian Brown	AUT	47.23/11.50	T-S	MDC-	T3r/T3	T3/T3	T-S
23	AUT_Enns_001_T3r_2	MZ901394	BT	Ennstaler Bergschecken	AUT	48.41/14.47	T-S	MDC-	T3r/T3	T3/T3	T-S
24	AUT_Enns_002_T3o_2	MZ901395	BT	Ennstaler Bergschecken	AUT	48.41/14.47	T-S	MDC-	T3o/T3o	T3/T3	T-S

No.	Fasta name	Acc. no.	SP	Breed	ORG	La-Y/Lo-X	Source	MDCT	MTP M/D	Beast M/D	Author
25	AUT_Enns_003_T3b_2	MZ901396	BT	Ennstaler Bergschecken	AUT	48.41/14.47	T-S	MDC-	T3b/T3	T3/T3	T-S
26	AUT_Enns_004_T3b_2	MZ901397	BT	Ennstaler Bergschecken	AUT	48.41/14.47	T-S	MDC-	T3b/T3	T3/T3	T-S
27	AUT_Murb_001_T3q_2	MZ901402	BT	Murbodner	AUT	46.94/15.35	T-S	MDC-	T3q/T3q	T3/T3	T-S
28	AUT_Murb_002_T3b_2	MZ901403	BT	Murbodner	AUT	46.94/15.35	T-S	MDC-	T3b/T3	T3/T3	T-S
29	AUT_Murb_003_P1x_2	MZ901408	BT	Murbodner	AUT	46.94/15.35	T-S	MDCT	P1/P	P1/P	T-S
30	AUT_Murb_004_T3r_2	MZ901409	BT	Murbodner	AUT	46.94/15.35	T-S	MDC-	T3r/T3	T3/T3	T-S
31	AUT_Murb_005_P1x_2	MZ901404	BT	Murbodner	AUT	46.94/15.35	T-S	MDC-	P1/P	P1/P	T-S
32	AUT_Murb_006_P1x_2	MZ901405	BT	Murbodner	AUT	46.94/15.35	T-S	MDC-	P1/P	P1/P	T-S
33	AUT_Murb_007_P1x_2	MZ901406	BT	Murbodner	AUT	46.94/15.35	T-S	MDC-	P1/P	P1/P	T-S
34	AUT_Murb_008_P1x_2	MZ901407	BT	Murbodner	AUT	46.94/15.35	T-S	MDC-	P1/P	P1/P	T-S
35	AUT_Pinz_001_T3b_2	MZ901412	BT	Original Pinzgauer	AUT	47.93/13.75	T-S	MDC-	T3b/T3p	T3/T3	T-S
36	AUT_Pinz_002_T3s_2	MZ901413	BT	Original Pinzgauer	AUT	47.93/13.75	T-S	MDC-	T3s/T3k	T3/T3	T-S
37	AUT_Pinz_003_T3b_2	MZ901414	BT	Original Pinzgauer	AUT	47.93/13.75	T-S	MDC-	T3b/T3p	T3/T3	T-S
38	AUT_Pinz_004_T3k_2	MZ901415	BT	Original Pinzgauer	AUT	47.93/13.75	T-S	MDC-	T3k/T3k	T3/T3	T-S
39	AUT_Pust_001_T3x_2	MZ901416	BT	Pustertaler Sprintzen	AUT	47.56/12.53	T-S	MDC-	T3/T3	T3/T3	T-S
40	AUT_Pust_002_T3r_2	MZ901417	BT	Pustertaler Sprintzen	AUT	47.56/12.53	T-S	MDC-	T3r/T3j	T3/T3	T-S
41	AUT_Pust_003_T3p_2	MZ901418	BT	Pustertaler Sprintzen	AUT	47.56/12.53	T-S	MDC-	T3p/T3p	T3/T3	T-S
42	AUT_Pust_004_T3r_2	MZ901419	BT	Pustertaler Sprintzen	AUT	47.56/12.53	T-S	MDCT	T3r/T3	T3/T3	T-S
43	AUT_Simm_001_T3j_2	MZ901420	BT	Fleckvieh	AUT	48.26/16.53	T-S	MDC-	T3j/T3	T3/T3	T-S
44	AUT_Simm_002_T3x_2	MZ901421	BT	Fleckvieh	AUT	48.26/16.53	T-S	MDC-	T3/T3	T3/T3	T-S
45	AUT_Simm_003_T3x_2	MZ901422	BT	Fleckvieh	AUT	48.26/16.53	T-S	MDC-	T3/T3	T3/T3	T-S
46	AUT_TuxZ_001_T3r_2	MZ901423	BT	Tux-Zillertaler	AUT	47.39/11.86	T-S	MDC-	T3r/T3j	T3/T3	T-S
47	AUT_TuxZ_002_T3r_2	MZ901424	BT	Tux-Zillertaler	AUT	47.39/11.86	T-S	MDC-	T3r/T3j	T3/T3	T-S
48	AUT_TuxZ_003_T3r_2	MZ901425	BT	Tux-Zillertaler	AUT	47.39/11.86	T-S	MDC-	T3r/T3	T3/T3	T-S
49	AUT_TuxZ_004_T3q_2	MZ901426	BT	Tux-Zillertaler	AUT	47.39/11.86	T-S	MDC-	T3q/T3q	T3/T3	T-S
50	AUT_VieB_001_T3x_2	MZ901398	BT	Karntner Blondvieh	AUT	46.69/14.12	T-S	MDC-	T3/T3	T3/T3	T-S
51	AUT_VieB_002_T3x_2	MZ901399	BT	Karntner Blondvieh	AUT	46.69/14.12	T-S	MDC-	T3/T3	T3/T3	T-S
52	AUT_VieB_003_T3r_2	MZ901400	BT	Karntner Blondvieh	AUT	46.69/14.12	T-S	MDC-	T3r/T3	T3/T3	T-S
53	AUT_VieB_004_T3x_2	MZ901401	BT	Karntner Blondvieh	AUT	46.69/14.12	T-S	MDC-	T3/T3	T3/T3	T-S

No.	Fasta name	Acc. no.	SP	Breed	ORG	La-Y/Lo-X	Source	MDCT	MTP M/D	Beast M/D	Author
54	AUT_WalB_001_T3q_2	MZ901427	BT	Waldviertler Blondvieh	AUT	47.30/15.93	T-S	MDC-	T3q/T3q	T3/T3	T-S
55	AUT_WalB_002_T3x_2	MZ901428	BT	Waldviertler Blondvieh	AUT	47.30/15.93	T-S	MDC-	T3/T3	T3/T3	T-S
56	AUT_WalB_003_T3x_2	MZ901429	BT	Waldviertler Blondvieh	AUT	47.30/15.93	T-S	MDC-	T3/T3	T3/T3	T-S
57	AUT_WalB_004_T3x_2	MZ901430	BT	Waldviertler Blondvieh	AUT	47.30/15.93	T-S	MDC-	T3/T3	T3/T3	T-S
58	BEL_BlBg_001_T3x_2	MZ901387	BT	Blanc Bleu Belge	BEL	50.70/5.60	T-S	MDC-	T3/T3	T3/T3	T-S
59	BEL_BlBg_002_T3r_2	MZ901388	BT	Blanc Bleu Belge	BEL	50.70/5.60	T-S	MDC-	T3r/T3j	T3/T3	T-S
60	BGR_ShoR_001_T3o_2	MZ901722	BT	Rhodopean Shorthorn	BGR	41.68/25.36	T-S	MDC-	T3o/T3o	T3/T3	T-S
61	BGR_ShoR_002_T3x_2	MZ901723	BT	Rhodopean Shorthorn	BGR	41.68/25.36	T-S	MDC-	T3/T3	T3/T3	T-S
62	BGR_ShoR_003_T3r_2	MZ901724	BT	Rhodopean Shorthorn	BGR	41.68/25.36	T-S	MDC-	T3r/T3i	T3/T3	T-S
63	BGR_ShoR_004_T3r_2	MZ901725	BT	Rhodopean Shorthorn	BGR	41.90/25.58	T-S	MDC-	T3r/T3	T3/T3	T-S
64	BGR_ShoR_005_T3x_2	MZ901726	BT	Rhodopean Shorthorn	BGR	41.90/25.58	T-S	MDC-	T3/T3	T3/T3	T-S
65	BGR_ShoR_006_T3x_2	MZ901727	BT	Rhodopean Shorthorn	BGR	41.58/24.68	T-S	MDC-	T3/T3	T3/T3	T-S
66	BGR_ShoR_007_T3x_2	MZ901728	BT	Rhodopean Shorthorn	BGR	41.58/24.68	T-S	MDC-	T3/T3	T3/T3	T-S
67	BGR_ShoR_008_T3x_2	MZ901729	BT	Rhodopean Shorthorn	BGR	41.68/25.36	T-S	MDC-	T3/T3	T3/T3	T-S
68	BIH_BuBo_001_T3x_2	MZ901389	BT	Bosnian and Herzegovinian Buša	BIH	43.35/17.60	T-S	MDC-	T3/T3	T3/T3	T-S
69	BIH_BuBo_002_T3x_2	MZ901390	BT	Bosnian and Herzegovinian Buša	BIH	43.35/17.60	T-S	MDC-	T3/T3	T3/T3	T-S
70	BIH_BuBo_003_T3p_2	MZ901391	BT	Bosnian and Herzegovinian Buša	BIH	43.35/17.60	T-S	MDC-	T3p/T3p	T3/T3	T-S
71	BIH_BuBo_004_T3x_2	MZ901392	BT	Bosnian and Herzegovinian Buša	BIH	43.35/17.60	T-S	MDC-	T3/T3	T3/T3	T-S
72	DEU_Brau_001_T3r_2	MZ901393	BT	Braunvieh	DEU	47.88/10.25	T-S	MDC-	T3r/T3j	T3/T3	T-S
73	DEU_MuWe_001_T3p_2	MZ901711	BT	Murnau-Werdenfelser	DEU	47.66/11.22	T-S	MDC-	T3p/T3p	T3/T3	T-S
74	DEU_MuWe_002_T3r_2	MZ901712	BT	Murnau-Werdenfelser	DEU	47.66/11.22	T-S	MDC-	T3r/T3	T3/T3	T-S
75	DEU_MuWe_003_T3q_2	MZ901713	BT	Murnau-Werdenfelser	DEU	47.66/11.22	T-S	MDC-	T3q/T3q	T3/T3	T-S
76	DEU_VieB_005_T3x_2	MZ901714	BT	Original Braunvieh	DEU	47.80/10.10	T-S	MDC-	T3/T3	T3/T3	T-S
77	DEU_VieB_006_T3x_2	MZ901715	BT	Original Braunvieh	DEU	47.80/10.10	T-S	MDC-	T3/T3	T3/T3	T-S
78	DEU_VieG_001_T3x_2	MZ901673	BT	Gelbvieh (German Yellow)	DEU	49.45/10.66	T-S	MDCT	T3/T3	T3/T3	T-S
79	DEU_VieG_002_T3x_2	MZ901674	BT	Gelbvieh (German Yellow)	DEU	49.45/10.66	T-S	MDC-	T3/T3	T3/T3	T-S
80	DEU_VieG_003_T3r_2	MZ901675	BT	Gelbvieh (German Yellow)	DEU	49.45/10.66	T-S	MDC-	T3r/T3d	T3/T3	T-S
81	DEU_VieG_004_T3x_2	MZ901676	BT	Gelbvieh (German Yellow)	DEU	49.45/10.66	T-S	MDC-	T3/T3	T3/T3	T-S
82	DEU_VieG_005_T3r_2	MZ901677	BT	Gelbvieh (German Yellow)	DEU	49.45/10.66	T-S	MDC-	T3r/T3	T3/T3	T-S

No.	Fasta name	Acc. no.	SP	Breed	ORG	La-Y/Lo-X	Source	MDCT	MTP M/D	Beast M/D	Author
83	ESP_Saya_001_T3x_2	MZ901748	BT	Sayaguesa	DEU	51.43/7.02	T-S	MDC-	T3/T3	T3/T3	T-S
84	ESP_Saya_002_T1x_2	MZ901749	BT	Sayaguesa	DEU	51.43/7.02	T-S	MDC-	T1/QT	T3/Q	T-S
85	ESP_Saya_003_T3d_2	MZ901750	BT	Sayaguesa	DEU	51.43/7.02	T-S	MDC-	T3d/T3	T3/T3	T-S
86	ESP_Saya_004_T3d_2	MZ901751	BT	Sayaguesa	DEU	51.43/7.02	T-S	MDC-	T3d/T3	T3/T3	T-S
87	FRA_BlAq_001_T3x_2	MZ901381	BT	Blonde d'Aquitaine	DEU	50.33/10.00	T-S	MDC-	T3/T3	T3/T3	T-S
88	FRA_BlAq_002_T3d_2	MZ901382	BT	Blonde d'Aquitaine	DEU	50.33/10.00	T-S	MDC-	T3d/T3d	T3/T3	T-S
89	FRA_BlAq_003_T3r_2	MZ901383	BT	Blonde d'Aquitaine	DEU	50.33/10.00	T-S	MDCT	T3r/T1e	T3/T3	T-S
90	FRA_Tare_001_T3r_2	MZ901752	BT	Tarentaise	FRA	45.35/5.77	T-S	MDC-	T3r/T1e	T3/T3	T-S
91	FRA_Tare_002_T3r_2	MZ901753	BT	Tarentaise	FRA	45.35/5.77	T-S	MDC-	T3r/T3	T3/T3	T-S
92	FRA_Tare_003_T3r_2	MZ901754	BT	Tarentaise	FRA	45.35/5.77	T-S	MDC-	T3r/T1e	T3/T3	T-S
93	GBR_Gall_001_T3d_2	MZ901678	BT	Galloway	DEU	48.77/12.40	T-S	MDC-	T3d/T3	T3/T3	T-S
94	GBR_Gall_002_T3q_2	MZ901679	BT	Galloway	DEU	48.77/12.40	T-S	MDC-	T3q/T3q	T3/T3	T-S
95	GRC_Cret_001_T3x_2	MZ901655	BT	Crete	GRC	35.16/24.55	T-S	MDC-	T3/T3	T3/T3	T-S
96	GRC_Cret_002_T3x_2	MZ901656	BT	Crete	GRC	35.16/24.55	T-S	MDC-	T3/T3	T3/T3	T-S
97	GRC_Kate_001_T3o_2	MZ901636	BT	Katerini	GRC	41.53/26.32	T-S	MDC-	T3o/T3o	T3/T3	T-S
98	GRC_Kate_002_T3r_2	MZ901637	BT	Katerini	GRC	41.53/26.32	T-S	MDCT	T3r/T3	T3/T3	T-S
99	GRC_Kate_003_T3r_2	MZ901638	BT	Katerini	GRC	41.53/26.32	T-S	MDC-	T3r/T3	T3/T3	T-S
100	GRC_Kate_004_Q1x_2	MZ901639	BT	Katerini	GRC	41.53/26.32	T-S	MDC-	Q1/QT	Q/Q	T-S
101	GRC_Kate_005_T2x_2	MZ901640	BT	Katerini	GRC	41.11/24.16	T-S	MDCT	T2/T2	T2/T2	T-S
102	GRC_Kate_006_T3x_2	MZ901641	BT	Katerini	GRC	41.11/24.16	T-S	MDC-	T3/T3	T3/T3	T-S
103	GRC_Kate_007_T3x_2	MZ901642	BT	Katerini	GRC	41.11/24.16	T-S	MDC-	T3/T3	T3/T3	T-S
104	GRC_KeaR_001_T2x_2	MZ901691	BT	Kea Rind	GRC	37.69/24.13	T-S	MDC-	T2/T2	T2/T2	T-S
105	GRC_KeaR_002_T2x_2	MZ901692	BT	Kea Rind	GRC	39.11/21.99	T-S	MDC-	T2/T2	T2/T2	T-S
106	GRC_KeaR_003_T2x_2	MZ901693	BT	Kea Rind	GRC	37.25/22.45	T-S	MDC-	T2/T2	T2/T2	T-S
107	GRC_KeaR_004_T3x_2	MZ901694	BT	Kea Rind	GRC	39.11/21.99	T-S	MDC-	T3/T3	T3/T3	T-S
108	GRC_Pres_005_T3x_2	MZ901643	BT	Greece Prespa	GRC	40.78/21.16	T-S	MDC-	T3/T3	T3/T3	T-S
109	GRC_Pres_006_T3o_2	MZ901644	BT	Greece Prespa	GRC	40.78/21.16	T-S	MDC-	T3o/T3o	T3/T3	T-S
110	GRC_Pres_007_T2x_2	MZ901645	BT	Greece Prespa	GRC	40.78/21.16	T-S	MDC-	T2/T2	T2/T2	T-S
111	GRC_Pres_008_T3h_2	MZ901646	BT	Greece Prespa	GRC	40.78/21.16	T-S	MDC-	T3h/T3	T3/T3	T-S

No.	Fasta name	Acc. no.	SP	Breed	ORG	La-Y/Lo-X	Source	MDCT	MTP M/D	Beast M/D	Author
112	GRC_ShoP_001_T3x_2	MZ901647	BT	Greece Shorthorn Pindos	GRC	39.59/21.51	T-S	MDC-	T3/T3	T3/T3	T-S
113	GRC_ShoP_002_Q1x_2	MZ901648	BT	Greece Shorthorn Pindos	GRC	39.59/21.51	T-S	MDC-	Q1/QT	Q/Q	T-S
114	GRC_ShoP_003_T3x_2	MZ901649	BT	Greece Shorthorn Pindos	GRC	39.59/21.51	T-S	MDCT-	T3/T3	T3/T3	T-S
115	GRC_ShoP_004_T3x_2	MZ901650	BT	Greece Shorthorn Pindos	GRC	39.59/21.51	T-S	MDC-	T3/T3	T3/T3	T-S
116	GRC_ShoR_001_T3q_2	MZ901651	BT	Greece Shorthorn Rodopi	GRC	41.12/25.56	T-S	MDC-	T3q/T3q	T3/T3	T-S
117	GRC_ShoR_002_Q1x_2	MZ901652	BT	Greece Shorthorn Rodopi	GRC	41.12/25.56	T-S	MDC-	Q1/T1a	Q/T1	T-S
118	GRC_ShoR_003_T3r_2	MZ901653	BT	Greece Shorthorn Rodopi	GRC	41.12/25.56	T-S	MDC-	T3r/T3	T3/T3	T-S
119	GRC_ShoR_004_T1x_2	MZ901654	BT	Greece Shorthorn Rodopi	GRC	41.12/25.56	T-S	MDC-	T1/T1	T1/T1	T-S
120	HRV_Bosk_001_T3c_2	MZ901608	BT	Boskarin (Istrian Cattle)	HRV	45.24/13.94	T-S	MDC-	T3c/T3e	T3/T3	T-S
121	HRV_Bosk_002_T3x_2	MZ901609	BT	Boskarin (Istrian Cattle)	HRV	45.24/13.94	T-S	MDC-	T3/T3	T3/T3	T-S
122	HRV_Bosk_003_T3r_2	MZ901610	BT	Boskarin (Istrian Cattle)	HRV	45.24/13.94	T-S	MDC-	T3r/T1b	T3/T3	T-S
123	HRV_Bosk_004_T3x_2	MZ901611	BT	Boskarin (Istrian Cattle)	HRV	45.24/13.94	T-S	MDC-	T3/T3r	T3/T3	T-S
124	HRV_Bosk_005_T3x_2	MZ901612	BT	Boskarin (Istrian Cattle)	HRV	45.24/13.94	T-S	MDC-	T3/T3e	T3/T3	T-S
125	HRV_Bosk_006_T3x_2	MZ901613	BT	Boskarin (Istrian Cattle)	HRV	45.24/13.94	T-S	MDC-	T3/T3k	T3/T3	T-S
126	HRV_Bosk_007_T3x_2	MZ901614	BT	Boskarin (Istrian Cattle)	HRV	45.24/13.94	T-S	MDC-	T3/T3i	T3/T3	T-S
127	HRV_Bosk_008_T3r_2	MZ901615	BT	Boskarin (Istrian Cattle)	HRV	45.24/13.94	T-S	MDC-	T3r/T3	T3/T3	T-S
128	HRV_Bosk_009_T3x_2	MZ901616	BT	Boskarin (Istrian Cattle)	HRV	45.24/13.94	T-S	MDC-	T3/T3	T3/T3	T-S
129	HRV_Bosk_010_T3r_2	MZ901617	BT	Boskarin (Istrian Cattle)	HRV	45.24/13.94	T-S	MDC-	T3r/T1b	T3/T3	T-S
130	HRV_Bosk_011_T3x_2	MZ901618	BT	Boskarin (Istrian Cattle)	HRV	45.24/13.94	T-S	MDC-	T3/T3	T3/T3	T-S
131	HRV_Bosk_012_T3x_2	MZ901619	BT	Boskarin (Istrian Cattle)	HRV	45.24/13.94	T-S	MDC-	T3/T3	T3/T3	T-S
132	HRV_Bosk_013_T3x_2	MZ901580	BT	Boskarin (Istrian Cattle)	HRV	45.24/13.94	T-S	MDC-	T3/T3i	T3/T3	T-S
133	HRV_Bosk_014_T3r_2	MZ901581	BT	Boskarin (Istrian Cattle)	HRV	45.24/13.94	T-S	MDC-	T3r/T1b	T3/T3	T-S
134	HRV_Bosk_015_T3x_2	MZ901582	BT	Boskarin (Istrian Cattle)	HRV	45.24/13.94	T-S	MDC-	T3/T3	T3/T3	T-S
135	HRV_Bosk_016_T3x_2	MZ901583	BT	Boskarin (Istrian Cattle)	HRV	45.24/13.94	T-S	MDC-	T3/T3i	T3/T3	T-S
136	HRV_Bosk_017_T3x_2	MZ901584	BT	Boskarin (Istrian Cattle)	HRV	45.24/13.94	T-S	MDC-	T3/T3	T3/T3	T-S
137	HRV_Bosk_018_T3x_2	MZ901585	BT	Boskarin (Istrian Cattle)	HRV	45.24/13.94	T-S	MDC-	T3/T1e	T3/T3	T-S
138	HRV_Bosk_019_T3c_2	MZ901586	BT	Boskarin (Istrian Cattle)	HRV	45.24/13.94	T-S	MDC-	T3c/T3e	T3/T3	T-S
139	HRV_Bosk_020_T3c_2	MZ901587	BT	Boskarin (Istrian Cattle)	HRV	45.24/13.94	T-S	MDC-	T3c/T3e	T3/T3	T-S
140	HRV_Bosk_021_T3r_2	MZ901588	BT	Boskarin (Istrian Cattle)	HRV	45.24/13.94	T-S	MDC-	T3r/T3	T3/T3	T-S

No.	Fasta name	Acc. no.	SP	Breed	ORG	La-Y/Lo-X	Source	MDCT	MTP M/D	Beast M/D	Author
141	HRV_Bosk_022_T3c_2	MZ901589	BT	Boskarin (Istrian Cattle)	HRV	45.24/13.94	T-S	MDC-	T3c/T3e	T3/T3	T-S
142	HRV_Bosk_023_T3o_2	MZ901590	BT	Boskarin (Istrian Cattle)	HRV	45.24/13.94	T-S	MDC-	T3o/T3e	T3/T3	T-S
143	HRV_Bosk_024_T3r_2	MZ901591	BT	Boskarin (Istrian Cattle)	HRV	45.24/13.94	T-S	MDC-	T3r/T1b	T3/T3	T-S
144	HRV_Bosk_025_T3q_2	MZ901592	BT	Boskarin (Istrian Cattle)	HRV	45.24/13.94	T-S	MDC-	T3q/T3q	T3/T3	T-S
145	HRV_Bosk_026_T3q_2	MZ901593	BT	Boskarin (Istrian Cattle)	HRV	45.24/13.94	T-S	MDC-	T3q/T3q	T3/T3	T-S
146	HRV_Bosk_027_T3j_2	MZ901594	BT	Boskarin (Istrian Cattle)	HRV	45.24/13.94	T-S	MDC-	T3j/T3	T3/T3	T-S
147	HRV_Bosk_028_T1x_2	MZ901595	BT	Boskarin (Istrian Cattle)	HRV	45.24/13.94	T-S	MDC-	T1/QT	T3/Q	T-S
148	HRV_Bosk_029_T1x_2	MZ901596	BT	Boskarin (Istrian Cattle)	HRV	45.24/13.94	T-S	MDC-	T1/QT	T3/Q	T-S
149	HRV_Bosk_030_T3x_2	MZ901597	BT	Boskarin (Istrian Cattle)	HRV	45.24/13.94	T-S	MDC-	T3/T3k	T3/T3	T-S
150	HRV_Bosk_031_T3r_2	MZ901598	BT	Boskarin (Istrian Cattle)	HRV	45.24/13.94	T-S	MDC-	T3r/T3	T3/T3	T-S
151	HRV_Bosk_032_T3j_2	MZ901599	BT	Boskarin (Istrian Cattle)	HRV	45.24/13.94	T-S	MDC-	T3j/T3	T3/T3	T-S
152	HRV_Bosk_033_T3p_2	MZ901600	BT	Boskarin (Istrian Cattle)	HRV	45.49/16.37	T-S	MDC-	T3p/T1e	T3/T3	T-S
153	HRV_Bosk_034_T3r_2	MZ901601	BT	Boskarin (Istrian Cattle)	HRV	45.24/13.94	T-S	MDC-	T3r/T3	T3/T3	T-S
154	HRV_Bosk_035_T3x_2	MZ901602	BT	Boskarin (Istrian Cattle)	HRV	45.24/13.94	T-S	MDC-	T3/T3r	T3/T3	T-S
155	HRV_Bosk_036_T3q_2	MZ901603	BT	Boskarin (Istrian Cattle)	HRV	45.24/13.94	T-S	MDC-	T3q/T3q	T3/T3	T-S
156	HRV_Bosk_037_T3q_2	MZ901604	BT	Boskarin (Istrian Cattle)	HRV	45.24/13.94	T-S	MDC-	T3q/T3q	T3/T3	T-S
157	HRV_Bosk_038_T3x_2	MZ901605	BT	Boskarin (Istrian Cattle)	HRV	45.24/13.94	T-S	MDC-	T3/T3	T3/T3	T-S
158	HRV_Bosk_039_T3x_2	MZ901606	BT	Boskarin (Istrian Cattle)	HRV	45.24/13.94	T-S	MDC-	T3/T3k	T3/T3	T-S
159	HRV_Bosk_040_T3c_2	MZ901607	BT	Boskarin (Istrian Cattle)	HRV	45.24/13.94	T-S	MDC-	T3c/T3e	T3/T3	T-S
160	HRV_BuHr_001_T1x_2	MZ901433	BT	Croatia Buša	HRV	45.59/17.22	T-S	MDC-	T1/QT	T3/T1	T-S
161	HRV_BuHr_002_T3r_2	MZ901434	BT	Croatia Buša	HRV	42.65/18.09	T-S	MDC-	T3r/T3	T3/T3	T-S
162	HRV_BuHr_003_T3x_2	MZ901435	BT	Croatia Buša	HRV	42.65/18.09	T-S	MDC-	T3/T3	T3/T3	T-S
163	HRV_BuHr_004_T3r_2	MZ901436	BT	Croatia Buša	HRV	42.65/18.09	T-S	MDC-	T3r/T3	T3/T3	T-S
164	HRV_BuHr_005_T3x_2	MZ901437	BT	Croatia Buša	HRV	42.65/18.09	T-S	MDC-	T3/T3	T3/T3	T-S
165	HRV_BuHr_006_T3x_2	MZ901438	BT	Croatia Buša	HRV	42.65/18.09	T-S	MDCT	T3/T3	T3/T3	T-S
166	HRV_BuHr_007_T3x_2	MZ901439	BT	Croatia Buša	HRV	42.65/18.09	T-S	MDC-	T3/T3b	T3/T3	T-S
167	HRV_BuHr_008_T1a_2	MZ901461	BT	Croatia Buša	HRV	45.59/17.22	T-S	MDC-	T1a/T1b	T3/T1	T-S
168	HRV_BuHr_009_T1f_2	MZ901462	BT	Croatia Buša	HRV	44.55/15.38	T-S	MDC-	T1f/T1f	T1/T1	T-S
169	HRV_BuHr_010_T2x_2	MZ901463	BT	Croatia Buša	HRV	42.65/18.09	T-S	MDC-	T2/T2	T2/T2	T-S

No.	Fasta name	Acc. no.	SP	Breed	ORG	La-Y/Lo-X	Source	MDCT	MTP M/D	Beast M/D	Author
170	HRV_BuHr_011_T3x_2	MZ901467	BT	Croatia Buša	HRV	43.74/15.90	T-S	MDC-	T3/T3r	T3/T3	T-S
171	HRV_BuHr_012_T3r_2	MZ901468	BT	Croatia Buša	HRV	43.74/15.90	T-S	MDC-	T3r/T3	T3/T3	T-S
172	HRV_BuHr_013_T3n_2	MZ901469	BT	Croatia Buša	HRV	43.51/16.44	T-S	MDC-	T3n/T3n	T3/T3	T-S
173	HRV_BuHr_014_T3x_2	MZ901431	BT	Croatia Buša	HRV	45.59/17.22	T-S	MDC-	T3/T3	T3/T3	T-S
174	HRV_BuHr_015_T2d_2	MZ901464	BT	Croatia Buša	HRV	45.59/17.22	T-S	MDC-	T2d/T2d	T2/T2	T-S
175	HRV_BuHr_016_T3x_2	MZ901432	BT	Croatia Buša	HRV	45.59/17.22	T-S	MDC-	T3/T3	T3/T3	T-S
176	HRV_BuHr_017_T3d_2	MZ901460	BT	Croatia Buša	HRV	45.59/17.22	T-S	MDC-	T3d/T3	T3/T3	T-S
177	HRV_BuHr_018_T3r_2	MZ901465	BT	Croatia Buša	HRV	43.74/15.90	T-S	MDC-	T3r/T3e	T3/T3	T-S
178	HRV_BuHr_019_T3x_2	MZ901466	BT	Croatia Buša	HRV	43.74/15.90	T-S	MDC-	T3/T3	T3/T3	T-S
179	HRV_BuHr_020_T3x_2	MZ901470	BT	Croatia Buša	HRV	43.51/16.44	T-S	MDC-	T3/T3	T3/T3	T-S
180	HRV_BuHr_021_T3x_2	MZ901440	BT	Croatia Buša	HRV	42.65/18.09	T-S	MDC-	T3/T1e	T3/T3	T-S
181	HRV_BuHr_022_T2d_2	MZ901441	BT	Croatia Buša	HRV	42.65/18.09	T-S	MDC-	T2d/T2d	T2/T2	T-S
182	HRV_BuHr_023_T3r_2	MZ901442	BT	Croatia Buša	HRV	44.55/15.38	T-S	MDC-	T3r/T3	T3/T3	T-S
183	HRV_BuHr_024_T2a_2	MZ901443	BT	Croatia Buša	HRV	44.55/15.38	T-S	MDC-	T2a/T2	T2/T2	T-S
184	HRV_BuHr_025_T3q_2	MZ901444	BT	Croatia Buša	HRV	44.55/15.38	T-S	MDC-	T3q/T3q	T3/T3	T-S
185	HRV_BuHr_026_T3p_2	MZ901445	BT	Croatia Buša	HRV	44.55/15.38	T-S	MDC-	T3p/T3p	T3/T3	T-S
186	HRV_BuHr_027_T3p_2	MZ901446	BT	Croatia Buša	HRV	44.55/15.38	T-S	MDC-	T3p/T3p	T3/T3	T-S
187	HRV_BuHr_028_T1a_2	MZ901447	BT	Croatia Buša	HRV	45.49/16.37	T-S	MDC-	T1a/T1b	T3/T1	T-S
188	HRV_BuHr_029_T3x_2	MZ901448	BT	Croatia Buša	HRV	45.49/16.37	T-S	MDC-	T3/T3	T3/T3	T-S
189	HRV_BuHr_030_T3q_2	MZ901449	BT	Croatia Buša	HRV	43.51/16.44	T-S	MDC-	T3q/T3q	T3/T3	T-S
190	HRV_BuHr_031_T3x_2	MZ901450	BT	Croatia Buša	HRV	43.51/16.44	T-S	MDC-	T3/T3	T3/T3	T-S
191	HRV_BuHr_032_T3r_2	MZ901451	BT	Croatia Buša	HRV	43.51/16.44	T-S	MDC-	T3r/T3	T3/T3	T-S
192	HRV_BuHr_033_T3r_2	MZ901452	BT	Croatia Buša	HRV	43.51/16.44	T-S	MDC-	T3r/T3j	T3/T3	T-S
193	HRV_BuHr_034_T3r_2	MZ901453	BT	Croatia Buša	HRV	43.51/16.44	T-S	MDC-	T3r/T3	T3/T3	T-S
194	HRV_BuHr_035_T3o_2	MZ901454	BT	Croatia Buša	HRV	43.51/16.44	T-S	MDC-	T3o/T3o	T3/T3	T-S
195	HRV_BuHr_036_T3r_2	MZ901455	BT	Croatia Buša	HRV	43.51/16.44	T-S	MDC-	T3r/T3	T3/T3	T-S
196	HRV_BuHr_037_T3r_2	MZ901456	BT	Croatia Buša	HRV	43.51/16.44	T-S	MDC-	T3r/T3	T3/T3	T-S
197	HRV_BuHr_038_T3r_2	MZ901457	BT	Croatia Buša	HRV	43.51/16.44	T-S	MDC-	T3r/T3e	T3/T3	T-S
198	HRV_BuHr_039_T3x_2	MZ901458	BT	Croatia Buša	HRV	43.51/16.44	T-S	MDC-	T3/T3	T3/T3	T-S

No.	Fasta name	Acc. no.	SP	Breed	ORG	La-Y/Lo-X	Source	MDCT	MTP M/D	Beast M/D	Author
199	HRV_BuHr_040_T3x_2	MZ901459	BT	Croatia Buša	HRV	43.51/16.44	T-S	MDC-	T3/T3e	T3/T3	T-S
200	HRV_Podo_001_T3r_2	MZ901620	BT	Slavonian Syrmian Podolian	HRV	45.63/18.69	T-S	MDCT	T3r/T3r	T3/T3	T-S
201	HRV_Podo_002_T3x_2	MZ901625	BT	Slavonian Syrmian Podolian	HRV	45.68/16.11	T-S	MDC-	T3/T3	T3/T3	T-S
202	HRV_Podo_003_T3x_2	MZ901626	BT	Slavonian Syrmian Podolian	HRV	45.68/16.11	T-S	MDC-	T3/T3	T3/T3	T-S
203	HRV_Podo_004_T3r_2	MZ901627	BT	Slavonian Syrmian Podolian	HRV	45.68/16.11	T-S	MDC-	T3r/T3	T3/T3	T-S
204	HRV_Podo_005_T3x_2	MZ901628	BT	Slavonian Syrmian Podolian	HRV	45.68/16.11	T-S	MDC-	T3/T3	T3/T3	T-S
205	HRV_Podo_006_T3x_2	MZ901629	BT	Slavonian Syrmian Podolian	HRV	45.68/16.11	T-S	MDC-	T3/T3	T3/T3	T-S
206	HRV_Podo_007_T3r_2	MZ901631	BT	Slavonian Syrmian Podolian	HRV	45.49/16.37	T-S	MDC-	T3r/T3	T3/T3	T-S
207	HRV_Podo_008_T3r_2	MZ901632	BT	Slavonian Syrmian Podolian	HRV	45.41/16.74	T-S	MDC-	T3r/T3	T3/T3	T-S
208	HRV_Podo_009_T3r_2	MZ901630	BT	Slavonian Syrmian Podolian	HRV	45.49/16.37	T-S	MDC-	T3r/T3r	T3/T3	T-S
209	HRV_Podo_010_T5b_2	MZ901633	BT	Slavonian Syrmian Podolian	HRV	45.63/18.69	T-S	MDC-	T5b/QT	T5/Q	T-S
210	HRV_Podo_011_T3r_2	MZ901634	BT	Slavonian Syrmian Podolian	HRV	45.63/18.69	T-S	MDC-	T3r/T3	T3/T3	T-S
211	HRV_Podo_012_T3r_2	MZ901635	BT	Slavonian Syrmian Podolian	HRV	45.49/16.37	T-S	MDC-	T3r/T3	T3/T3	T-S
212	HRV_Podo_013_T5b_2	MZ901621	BT	Slavonian Syrmian Podolian	HRV	45.16/18.01	T-S	MDC-	T5b/QT	T5/Q	T-S
213	HRV_Podo_014_T3x_2	MZ901622	BT	Slavonian Syrmian Podolian	HRV	45.16/18.01	T-S	MDC-	T3/T3	T3/T3	T-S
214	HRV_Podo_015_T5b_2	MZ901623	BT	Slavonian Syrmian Podolian	HRV	45.63/18.69	T-S	MDC-	T5b/QT	T5/Q	T-S
215	HRV_Podo_016_T3r_2	MZ901624	BT	Slavonian Syrmian Podolian	HRV	45.63/18.69	T-S	MDC-	T3r/T3	T3/T3	T-S
216	KOS_BuDi_001_T2x_2	MZ901669	BT	Dukagjini Buša	XKX	42.55/20.50	T-S	MDC-	T2/T2	T2/T2	T-S
217	KOS_BuDi_002_T2x_2	MZ901670	BT	Dukagjini Buša	XKX	42.55/20.50	T-S	MDC-	T2/T2	T2/T2	T-S
218	KOS_BuDi_003_T2x_2	MZ901671	BT	Dukagjini Buša	XKX	42.55/20.50	T-S	MDC-	T2/T2	T2/T2	T-S
219	KOS_BuDi_004_T2x_2	MZ901672	BT	Dukagjini Buša	XKX	42.55/20.50	T-S	MDC-	T2/T2	T2/T2	T-S
220	KOS_BuRM_001_T3r_2	MZ901687	BT	Red Metohian Buša	XKX	41.96/20.70	T-S	MDC-	T3r/T1e	T3/T3	T-S
221	KOS_BuRM_002_T3x_2	MZ901688	BT	Red Metohian Buša	XKX	41.96/20.70	T-S	MDC-	T3/T3	T3/T3	T-S
222	KOS_BuRM_003_T3h_2	MZ901689	BT	Red Metohian Buša	XKX	41.96/20.70	T-S	MDCT	T3h/T3	T3/T3	T-S
223	KOS_BuRM_004_T3b_2	MZ901690	BT	Red Metohian Buša	XKX	41.96/20.70	T-S	MDC-	T3b/T3	T3/T3	T-S
224	KOS_BuSh_001_T3r_2	MZ901736	BT	Sharri Buša	XKX	42.00/20.66	T-S	MDC-	T3r/T3	T3/T3	T-S
225	KOS_BuSh_002_T3x_2	MZ901737	BT	Sharri Buša	XKX	42.00/20.66	T-S	MDC-	T3/T3	T3/T3	T-S
226	KOS_BuSh_003_T3q_2	MZ901738	BT	Sharri Buša	XKX	42.00/20.66	T-S	MDC-	T3q/T3q	T3/T3	T-S
227	KOS_BuSh_004_T3s_2	MZ901739	BT	Sharri Buša	XKX	42.00/20.66	T-S	MDC-	T3s/T3	T3/T3	T-S

No.	Fasta name	Acc. no.	SP	Breed	ORG	La-Y/Lo-X	Source	MDCT	MTP M/D	Beast M/D	Author
228	MKD_BuMd_001_T1x_2	MZ901703	BT	Macedonian Buša	MKD	41.12/21.60	T-S	MDC-	T1/T1	T1/T1	T-S
229	MKD_BuMd_002_T1x_2	MZ901704	BT	Macedonian Buša	MKD	41.12/21.60	T-S	MDC-	T1/T1	T1/T1	T-S
230	MKD_BuMd_003_T3b_2	MZ901705	BT	Macedonian Buša	MKD	41.12/21.60	T-S	MDC-	T3b/T3	T3/T3	T-S
231	MKD_BuMd_004_T3x_2	MZ901706	BT	Macedonian Buša	MKD	41.12/21.60	T-S	MDC-	T3/T3	T3/T3	T-S
232	MKD_BuMd_005_T3r_2	MZ901707	BT	Macedonian Buša	MKD	41.12/21.60	T-S	MDC-	T3r/T3	T3/T3	T-S
233	MNE_BuCG_001_T3x_2	MZ901708	BT	Montenegro Buša	MNE	41.90/19.30	T-S	MDC-	T3/T3	T3/T3	T-S
234	MNE_BuCG_002_T3x_2	MZ901709	BT	Montenegro Buša	MNE	42.70/19.30	T-S	MDC-	T3/T3	T3/T3	T-S
235	MNE_BuCG_003_T3x_2	MZ901710	BT	Montenegro Buša	MNE	42.70/19.30	T-S	MDC-	T3/T3	T3/T3	T-S
236	MNG_Tura_001_T3n_2	MZ901756	BT	Turano-Mongolian	MNG	47.85/112.50	T-S	MDC-	T3n/T3n	T3/T3	T-S
237	MNG_Tura_002_T3b_2	MZ901757	BT	Turano-Mongolian	MNG	47.85/112.50	T-S	MDC-	T3b/T3	T3/T3	T-S
238	MNG_Tura_003_T3d_2	MZ901759	BT	Turano-Mongolian	MNG	47.85/112.50	T-S	MDC-	T3d/T3	T3/T3	T-S
239	MNG_Tura_004_T2x_2	MZ901758	BT	Turano-Mongolian	MNG	47.85/112.50	T-S	MDC-	T2/T2	T2/T2	T-S
240	MNG_Tura_005_T3d_2	MZ901755	BT	Turano-Mongolian	MNG	47.85/112.50	T-S	MDC-	T3d/T3	T3/T3	T-S
241	NLD_HoFr_017_T3x_2	MZ901471	BT	Holstein	HRV	45.92/16.69	T-S	MDC-	T3/T3	T3/T3	T-S
242	NLD_HoFr_018_T3x_2	MZ901472	BT	Holstein	HRV	45.90/16.64	T-S	MDC-	T3/Q1	T3/T3	T-S
243	NLD_HoFr_019_T3r_2	MZ901473	BT	Holstein	HRV	45.92/16.65	T-S	MDC-	T3r/T3	T3/T3	T-S
244	NLD_HoFr_020_T3x_2	MZ901474	BT	Holstein	HRV	45.90/16.30	T-S	MDC-	T3/T3	T3/T3	T-S
245	NLD_HoFr_021_T3x_2	MZ901475	BT	Holstein	HRV	45.93/16.45	T-S	MDC-	T3/T3	T3/T3	T-S
246	NLD_HoFr_022_T3r_2	MZ901476	BT	Holstein	HRV	45.90/16.30	T-S	MDC-	T3r/T3j	T3/T3	T-S
247	NLD_HoFr_023_T3x_2	MZ901481	BT	Holstein	HRV	44.92/15.14	T-S	MDC-	T3/T3	T3/T3	T-S
248	NLD_HoFr_024_T3g_2	MZ901486	BT	Holstein	HRV	44.91/15.12	T-S	MDC-	T3g/T3q	T3/T3	T-S
249	NLD_HoFr_025_T3r_2	MZ901492	BT	Holstein	HRV	45.79/18.54	T-S	MDC-	T3r/T3	T3/T3	T-S
250	NLD_HoFr_026_T3r_2	MZ901497	BT	Holstein	HRV	45.59/18.45	T-S	MDC-	T3r/T3	T3/T3	T-S
251	NLD_HoFr_027_T3x_2	MZ901504	BT	Holstein	HRV	45.59/18.45	T-S	MDC-	T3/T3	T3/T3	T-S
252	NLD_HoFr_028_T3x_2	MZ901511	BT	Holstein	HRV	45.59/18.45	T-S	MDC-	T3/T3	T3/T3	T-S
253	NLD_HoFr_029_T3h_2	MZ901516	BT	Holstein	HRV	45.59/18.45	T-S	MDC-	T3h/T3	T3/T3	T-S
254	NLD_HoFr_030_T3x_2	MZ901536	BT	Holstein	HRV	45.43/18.41	T-S	MDC-	T3/T3	T3/T3	T-S
255	NLD_HoFr_031_T3x_2	MZ901537	BT	Holstein	HRV	45.43/18.41	T-S	MDC-	T3/T3	T3/T3	T-S
256	NLD_HoFr_032_T3x_2	MZ901538	BT	Holstein	HRV	45.43/18.41	T-S	MDC-	T3/T3	T3/T3	T-S

No.	Fasta name	Acc. no.	SP	Breed	ORG	La-Y/Lo-X	Source	MDCT	MTP M/D	Beast M/D	Author
257	NLD_HoFr_033_T3x_2	MZ901539	BT	Holstein	HRV	45.43/18.41	T-S	MDC-	T3/T1e	T3/T3	T-S
258	NLD_HoFr_034_T3x_2	MZ901540	BT	Holstein	HRV	45.43/18.41	T-S	MDC-	T3/T1e	T3/T3	T-S
259	NLD_HoFr_035_T3x_2	MZ901541	BT	Holstein	HRV	45.43/18.41	T-S	MDC-	T3/T3	T3/T3	T-S
260	NLD_HoFr_036_T3b_2	MZ901542	BT	Holstein	HRV	45.43/18.41	T-S	MDC-	T3b/T3	T3/T3	T-S
261	NLD_HoFr_037_T3b_2	MZ901543	BT	Holstein	HRV	44.65/15.38	T-S	MDC-	T3b/T3e	T3/T3	T-S
262	NLD_HoFr_038_T3g_2	MZ901544	BT	Holstein	HRV	45.49/18.10	T-S	MDC-	T3g/T3	T3/T3	T-S
263	NLD_HoFr_039_T3r_2	MZ901545	BT	Holstein	HRV	45.49/18.10	T-S	MDC-	T3r/T3	T3/T3	T-S
264	NLD_HoFr_040_T3r_2	MZ901546	BT	Holstein	HRV	45.49/18.10	T-S	MDC-	T3r/T1e	T3/T3	T-S
265	NLD_HoFr_041_T3x_2	MZ901547	BT	Holstein	HRV	45.49/18.10	T-S	MDC-	T3/T1e	T3/T3	T-S
266	NLD_HoFr_042_T3x_2	MZ901548	BT	Holstein	HRV	45.49/18.10	T-S	MDC-	T3/T3	T3/T3	T-S
267	NLD_HoFr_043_T3x_2	MZ901549	BT	Holstein	HRV	45.49/18.10	T-S	MDC-	T3/T3	T3/T3	T-S
268	NLD_HoFr_044_T3r_2	MZ901550	BT	Holstein	HRV	45.49/18.10	T-S	MDC-	T3r/T3j	T3/T3	T-S
269	NLD_HoFr_045_T3r_2	MZ901551	BT	Holstein	HRV	45.49/18.10	T-S	MDC-	T3r/T3	T3/T3	T-S
270	NLD_HoFr_046_T3x_2	MZ901552	BT	Holstein	HRV	45.93/16.73	T-S	MDC-	T3/T3	T3/T3	T-S
271	NLD_HoFr_047_T3x_2	MZ901553	BT	Holstein	HRV	45.93/16.73	T-S	MDC-	T3/T3	T3/T3	T-S
272	NLD_HoFr_048_T3x_2	MZ901554	BT	Holstein	HRV	45.93/16.73	T-S	MDC-	T3/T3	T3/T3	T-S
273	NLD_HoFr_049_T3x_2	MZ901555	BT	Holstein	HRV	45.93/16.73	T-S	MDC-	T3/T3	T3/T3	T-S
274	NLD_HoFr_050_T2x_2	MZ901556	BT	Holstein	HRV	45.51/18.88	T-S	MDC-	T2/T2	T2/T2	T-S
275	NLD_HoFr_051_T3b_2	MZ901557	BT	Holstein	HRV	44.18/15.19	T-S	MDC-	T3b/T3	T3/T3	T-S
276	NLD_HoFr_052_T3h_2	MZ901558	BT	Holstein	HRV	44.18/15.19	T-S	MDC-	T3h/T3	T3/T3	T-S
277	NLD_HoFr_053_T3x_2	MZ901559	BT	Holstein	HRV	44.18/15.19	T-S	MDC-	T3/T3	T3/T3	T-S
278	NLD_HoFr_054_T3o_2	MZ901560	BT	Holstein	HRV	44.18/15.19	T-S	MDC-	T3o/T3o	T3/T3	T-S
279	NLD_HoFr_055_T3r_2	MZ901561	BT	Holstein	HRV	44.18/15.19	T-S	MDC-	T3r/T3o	T3/T3	T-S
280	NLD_HoFr_056_T3r_2	MZ901562	BT	Holstein	HRV	44.18/15.19	T-S	MDC-	T3r/T3o	T3/T3	T-S
281	NLD_HoFr_057_T3x_2	MZ901563	BT	Holstein	HRV	44.18/15.19	T-S	MDC-	T3/T3	T3/T3	T-S
282	NLD_HoFr_058_T3d_2	MZ901564	BT	Holstein	HRV	44.18/15.19	T-S	MDC-	T3d/T3	T3/T3	T-S
283	NLD_HoFr_059_T3x_2	MZ901565	BT	Holstein	HRV	44.18/15.19	T-S	MDC-	T3/T3	T3/T3	T-S
284	NLD_HoFr_060_T3x_2	MZ901566	BT	Holstein	HRV	44.18/15.19	T-S	MDC-	T3/T3	T3/T3	T-S
285	NLD_HoFr_061_T3x_2	MZ901567	BT	Holstein	HRV	44.18/15.19	T-S	MDC-	T3/T3	T3/T3	T-S

No.	Fasta name	Acc. no.	SP	Breed	ORG	La-Y/Lo-X	Source	MDCT	MTP M/D	Beast M/D	Author
286	NLD_HoFr_062_T3x_2	MZ901568	BT	Holstein	HRV	44.18/15.19	T-S	MDC-	T3/T1b	T3/T3	T-S
287	NLD_HoFr_063_T1b_2	MZ901569	BT	Holstein	HRV	44.18/15.19	T-S	MDC-	T1b/T1b	T3/T3	T-S
288	NLD_HoFr_064_T3r_2	MZ901570	BT	Holstein	HRV	44.18/15.19	T-S	MDC-	T3r/T3	T3/T3	T-S
289	NLD_HoFr_065_T3x_2	MZ901571	BT	Holstein	HRV	43.96/15.47	T-S	MDC-	T3/T3	T3/T3	T-S
290	NLD_HoFr_066_T3n_2	MZ901572	BT	Holstein	HRV	43.96/15.47	T-S	MDC-	T3n/T3n	T3/T3	T-S
291	NLD_HoFr_067_T3n_2	MZ901573	BT	Holstein	HRV	43.96/15.47	T-S	MDC-	T3n/T3n	T3/T3	T-S
292	NLD_HoFr_068_T3r_2	MZ901574	BT	Holstein	HRV	43.96/15.47	T-S	MDC-	T3r/T3	T3/T3	T-S
293	NLD_HoFr_069_T3r_2	MZ901575	BT	Holstein	HRV	43.96/15.47	T-S	MDC-	T3r/T3	T3/T3	T-S
294	NLD_HoFr_070_T3p_2	MZ901576	BT	Holstein	HRV	43.96/15.47	T-S	MDC-	T3p/T3i	T3/T3	T-S
295	NLD_HoFr_071_T3x_2	MZ901577	BT	Holstein	HRV	43.96/15.47	T-S	MDC-	T3/T3	T3/T3	T-S
296	NLD_HoFr_072_T3x_2	MZ901578	BT	Holstein	HRV	43.96/15.47	T-S	MDC-	T3/T3	T3/T3	T-S
297	NLD_HoFr_073_T3x_2	MZ901579	BT	Holstein	HRV	43.96/15.47	T-S	MDC-	T3/T3	T3/T3	T-S
298	NLD_HoFr_074_T3r_2	MZ901477	BT	Holstein	HRV	45.63/18.69	T-S	MDC-	T3r/T3	T3/T3	T-S
299	NLD_HoFr_075_T3x_2	MZ901478	BT	Holstein	HRV	45.63/18.69	T-S	MDC-	T3/T3	T3/T3	T-S
300	NLD_HoFr_076_T3x_2	MZ901479	BT	Holstein	HRV	45.63/18.69	T-S	MDC-	T3/T3	T3/T3	T-S
301	NLD_HoFr_077_T3x_2	MZ901480	BT	Holstein	HRV	45.63/18.69	T-S	MDC-	T3/T3	T3/T3	T-S
302	NLD_HoFr_078_T3x_2	MZ901482	BT	Holstein	HRV	45.63/18.69	T-S	MDC-	T3/T3	T3/T3	T-S
303	NLD_HoFr_079_T3b_2	MZ901483	BT	Holstein	HRV	45.63/18.69	T-S	MDC-	T3b/T3	T3/T3	T-S
304	NLD_HoFr_080_T3x_2	MZ901484	BT	Holstein	HRV	45.63/18.69	T-S	MDC-	T3/T3	T3/T3	T-S
305	NLD_HoFr_081_T3x_2	MZ901485	BT	Holstein	HRV	45.63/18.69	T-S	MDC-	T3/T3	T3/T3	T-S
306	NLD_HoFr_082_T3x_2	MZ901487	BT	Holstein	HRV	45.33/19.00	T-S	MDC-	T3/T3	T3/T3	T-S
307	NLD_HoFr_083_T3r_2	MZ901488	BT	Holstein	HRV	45.33/19.00	T-S	MDC-	T3r/T3	T3/T3	T-S
308	NLD_HoFr_084_T3r_2	MZ901489	BT	Holstein	HRV	45.33/19.00	T-S	MDC-	T3r/T3	T3/T3	T-S
309	NLD_HoFr_085_T3r_2	MZ901490	BT	Holstein	HRV	45.33/19.00	T-S	MDC-	T3r/T3	T3/T3	T-S
310	NLD_HoFr_086_T3r_2	MZ901491	BT	Holstein	HRV	45.33/19.00	T-S	MDC-	T3r/T3	T3/T3	T-S
311	NLD_HoFr_087_T3g_2	MZ901493	BT	Holstein	HRV	45.08/18.70	T-S	MDC-	T3g/T3	T3/T3	T-S
312	NLD_HoFr_088_T3x_2	MZ901494	BT	Holstein	HRV	45.08/18.70	T-S	MDC-	T3/T3r	T3/T3	T-S
313	NLD_HoFr_089_T3r_2	MZ901495	BT	Holstein	HRV	45.08/18.70	T-S	MDC-	T3r/T3	T3/T3	T-S
314	NLD_HoFr_090_T3r_2	MZ901496	BT	Holstein	HRV	45.08/18.70	T-S	MDC-	T3r/T3	T3/T3	T-S

No.	Fasta name	Acc. no.	SP	Breed	ORG	La-Y/Lo-X	Source	MDCT	MTP M/D	Beast M/D	Author
315	NLD_HoFr_091_T3b_2	MZ901498	BT	Holstein	HRV	45.29/18.68	T-S	MDC-	T3b/T3	T3/T3	T-S
316	NLD_HoFr_092_T3x_2	MZ901499	BT	Holstein	HRV	45.29/18.68	T-S	MDC-	T3/T3b	T3/T3	T-S
317	NLD_HoFr_093_T3x_2	MZ901500	BT	Holstein	HRV	45.29/18.68	T-S	MDC-	T3/T3	T3/T3	T-S
318	NLD_HoFr_094_T3b_2	MZ901501	BT	Holstein	HRV	45.29/18.68	T-S	MDC-	T3b/T3	T3/T3	T-S
319	NLD_HoFr_095_T3r_2	MZ901502	BT	Holstein	HRV	45.29/18.68	T-S	MDC-	T3r/T3	T3/T3	T-S
320	NLD_HoFr_096_T3r_2	MZ901503	BT	Holstein	HRV	45.29/18.68	T-S	MDC-	T3r/T3	T3/T3	T-S
321	NLD_HoFr_097_T3x_2	MZ901505	BT	Holstein	HRV	45.29/18.68	T-S	MDC-	T3/T3	T3/T3	T-S
322	NLD_HoFr_098_T3r_2	MZ901506	BT	Holstein	HRV	45.29/18.68	T-S	MDC-	T3r/T3	T3/T3	T-S
323	NLD_HoFr_099_T3g_2	MZ901507	BT	Holstein	HRV	45.29/18.68	T-S	MDC-	T3g/T3	T3/T3	T-S
324	NLD_HoFr_100_T3r_2	MZ901508	BT	Holstein	HRV	45.29/18.68	T-S	MDC-	T3r/T1e	T3/T3	T-S
325	NLD_HoFr_101_T3r_2	MZ901509	BT	Holstein	HRV	45.29/18.68	T-S	MDC-	T3r/T4	T3/T3	T-S
326	NLD_HoFr_102_T3g_2	MZ901510	BT	Holstein	HRV	45.29/18.68	T-S	MDC-	T3g/T3	T3/T3	T-S
327	NLD_HoFr_103_T3r_2	MZ901512	BT	Holstein	HRV	45.29/18.68	T-S	MDC-	T3r/T3	T3/T3	T-S
328	NLD_HoFr_104_T3x_2	MZ901513	BT	Holstein	HRV	45.88/16.77	T-S	MDC-	T3/T3	T3/T3	T-S
329	NLD_HoFr_105_T3p_2	MZ901514	BT	Holstein	HRV	45.88/16.77	T-S	MDC-	T3p/T3p	T3/T3	T-S
330	NLD_HoFr_106_T3x_2	MZ901515	BT	Holstein	HRV	45.80/16.74	T-S	MDC-	T3/T3	T3/T3	T-S
331	NLD_HoFr_107_T3r_2	MZ901517	BT	Holstein	HRV	45.87/16.74	T-S	MDC-	T3r/T3	T3/T3	T-S
332	NLD_HoFr_108_T3x_2	MZ901518	BT	Holstein	HRV	45.79/17.02	T-S	MDC-	T3/T3	T3/T3	T-S
333	NLD_HoFr_109_T2x_2	MZ901519	BT	Holstein	HRV	45.99/16.81	T-S	MDC-	T2/T2	T2/T2	T-S
334	NLD_HoFr_110_T3r_2	MZ901520	BT	Holstein	HRV	45.99/16.81	T-S	MDC-	T3r/T3j	T3/T3	T-S
335	NLD_HoFr_111_T3r_2	MZ901521	BT	Holstein	HRV	45.78/16.67	T-S	MDC-	T3r/T3j	T3/T3	T-S
336	NLD_HoFr_112_T3x_2	MZ901522	BT	Holstein	HRV	46.01/16.83	T-S	MDC-	T3/T3	T3/T3	T-S
337	NLD_HoFr_113_T3n_2	MZ901523	BT	Holstein	HRV	45.80/16.70	T-S	MDC-	T3n/T3n	T3/T3	T-S
338	NLD_HoFr_114_T3r_2	MZ901524	BT	Holstein	HRV	45.83/17.04	T-S	MDC-	T3r/T1e	T3/T3	T-S
339	NLD_HoFr_115_T3r_2	MZ901525	BT	Holstein	HRV	45.88/16.77	T-S	MDC-	T3r/T3j	T3/T3	T-S
340	NLD_HoFr_116_T3r_2	MZ901526	BT	Holstein	HRV	46.02/16.75	T-S	MDC-	T3r/T3	T3/T3	T-S
341	NLD_HoFr_117_T3r_2	MZ901527	BT	Holstein	HRV	45.77/17.08	T-S	MDC-	T3r/T3	T3/T3	T-S
342	NLD_HoFr_118_T3r_2	MZ901528	BT	Holstein	HRV	45.77/17.02	T-S	MDC-	T3r/T3	T3/T3	T-S
343	NLD_HoFr_119_T3r_2	MZ901529	BT	Holstein	HRV	45.66/16.92	T-S	MDC-	T3r/T3	T3/T3	T-S

No.	Fasta name	Acc. no.	SP	Breed	ORG	La-Y/Lo-X	Source	MDCT	MTP M/D	Beast M/D	Author
344	NLD_HoFr_120_T5x_2	MZ901530	BT	Holstein	HRV	45.68/17.12	T-S	MDC-	T5/QT	T5/Q	T-S
345	NLD_HoFr_121_T3x_2	MZ901531	BT	Holstein	HRV	45.69/16.99	T-S	MDC-	T3/T3	T3/T3	T-S
346	NLD_HoFr_122_T3x_2	MZ901532	BT	Holstein	HRV	44.18/15.19	T-S	MDC-	T3/T3b	T3/T3	T-S
347	NLD_HoFr_123_T3n_2	MZ901533	BT	Holstein	HRV	44.87/13.86	T-S	MDC-	T3n/T3n	T3/T3	T-S
348	NLD_HoFr_124_T3x_2	MZ901534	BT	Holstein	HRV	44.90/13.91	T-S	MDC-	T3/T3	T3/T3	T-S
349	NLD_HoFr_125_T3x_2	MZ901535	BT	Holstein	HRV	45.38/13.62	T-S	MDC-	T3/T3	T3/T3	T-S
350	NLD_HoFr_126_T3b_2	MZ901720	BT	Red Holstein	DEU	50.28/6.90	T-S	MDC-	T3b/T3	T3/T3	T-S
351	NLD_HoFr_127_T3h_2	MZ901721	BT	Red Holstein	DEU	50.28/6.90	T-S	MDC-	T3h/T3r	T3/T3	T-S
352	PRT_Barr_001_T3x_2	MZ901384	BT	Barrosa	PRT	41.45/-8.40	T-S	MDC-	T3/T3c	T3/T3	T-S
353	PRT_Barr_002_T1a_2	MZ901385	BT	Barrosa	PRT	41.45/-8.40	T-S	MDC-	T1a/T1b	T1/T1	T-S
354	PRT_Barr_003_T1a_2	MZ901386	BT	Barrosa	PRT	41.45/-8.40	T-S	MDC-	T1a/T1	T1/T1	T-S
355	PRT_Maro_001_T3r_2	MZ901699	BT	Maronesa	PRT	41.25/-7.62	T-S	MDC-	T3r/T3	T3/T3	T-S
356	PRT_Maro_002_T3r_2	MZ901700	BT	Maronesa	PRT	41.25/-7.62	T-S	MDC-	T3r/T3	T3/T3	T-S
357	PRT_Maro_003_T3r_2	MZ901701	BT	Maronesa	PRT	41.25/-7.62	T-S	MDC-	T3r/T3	T3/T3	T-S
358	PRT_Maro_004_T3r_2	MZ901702	BT	Maronesa	PRT	41.25/-7.62	T-S	MDC-	T3r/T3	T3/T3	T-S
359	RUS_Jaku_001_T3n_2	MZ901730	BT	Yakutian	RUS	67.75/130.30	T-S	MDC-	T3n/T3n	T3/T3	T-S
360	RUS_Jaku_002_T3r_2	MZ901733	BT	Yakutian	RUS	67.75/130.30	T-S	MDC-	T3r/T3j	T3/T3	T-S
361	RUS_Jaku_003_T2x_2	MZ901734	BT	Yakutian	RUS	68.30/132.88	T-S	MDC-	T2/T2	T2/T2	T-S
362	RUS_Jaku_004_T3k_2	MZ901735	BT	Yakutian	RUS	68.15/132.67	T-S	MDC-	T3k/T3k	T3/T3	T-S
363	RUS_Jaku_005_T3n_2	MZ901731	BT	Yakutian	RUS	67.75/130.30	T-S	MDC-	T3n/T3n	T3/T3	T-S
364	RUS_Jaku_006_T3r_2	MZ901732	BT	Yakutian	RUS	67.75/130.30	T-S	MDC-	T3r/T3j	T3/T3	T-S
365	SCO_Here_001_T3x_2	MZ901680	BT	Hereford	DEU	48.77/12.40	T-S	MDCT	T3/T1e	T3/T3	T-S
366	SCO_Here_002_T3r_2	MZ901681	BT	Hereford	DEU	52.20/13.10	T-S	MDC-	T3r/T1e	T3/T3	T-S
367	SCO_Here_003_T3x_2	MZ901682	BT	Hereford	DEU	52.20/13.10	T-S	MDC-	T3/T3	T3/T3	T-S
368	SCO_ScHi_001_T3g_2	MZ901683	BT	Highland	DEU	53.95/9.70	T-S	MDC-	T3g/T3	T3/T3	T-S
369	SCO_ScHi_002_T3r_2	MZ901684	BT	Highland	DEU	53.95/9.70	T-S	MDC-	T3r/T1e	T3/T3	T-S
370	SRB_BuSr_001_T3x_2	MZ901744	BT	Serbian Buša	SRB	43.26/22.67	T-S	MDC-	T3/T3	T3/T3	T-S
371	SRB_BuSr_002_T3r_2	MZ901745	BT	Serbian Buša	SRB	43.26/22.67	T-S	MDC-	T3r/T3	T3/T3	T-S
372	SRB_BuSr_003_T3x_2	MZ901746	BT	Serbian Buša	SRB	43.26/22.67	T-S	MDC-	T3/T3	T3/T3	T-S

No.	Fasta name	Acc. no.	SP	Breed	ORG	La-Y/Lo-X	Source	MDCT	MTP M/D	Beast M/D	Author
373	SRB_BuSr_004_T3x_2	MZ901747	BT	Serbian Buša	SRB	43.26/22.67	T-S	MDC-	T3/T3	T3/T3	T-S
374	SVN_Cika_001_T3r_2	MZ901657	BT	Cika	SVN	46.01/14.72	T-S	MDC-	T3r/T3	T3/T3	T-S
375	SVN_Cika_002_T3x_2	MZ901658	BT	Cika	SVN	46.01/14.72	T-S	MDC-	T3/T3	T3/T3	T-S
376	SVN_Cika_003_T3r_2	MZ901659	BT	Cika	SVN	46.01/14.72	T-S	MDC-	T3r/T3q	T3/T3	T-S
377	SVN_Cika_004_T3x_2	MZ901660	BT	Cika	SVN	46.01/14.72	T-S	MDC-	T3/T3	T3/T3	T-S
378	SVN_Cika_005_T3r_2	MZ901661	BT	Cika	SVN	46.01/14.72	T-S	MDC-	T3r/T3q	T3/T3	T-S
379	SVN_Cika_006_T3r_2	MZ901662	BT	Cika	SVN	46.01/14.72	T-S	MDCT	T3r/T3q	T3/T3	T-S
380	SVN_Cika_007_T3x_2	MZ901663	BT	Cika	SVN	46.01/14.72	T-S	MDC-	T3/T3	T3/T3	T-S
381	SVN_Cika_008_T3x_2	MZ901664	BT	Cika	SVN	46.01/14.72	T-S	MDC-	T3/T3	T3/T3	T-S
382	TUR_AnaB_001_T3r_2	MZ901378	BT	Anatolian Black	TUR	39.00/35.00	T-S	MDC-	T3r/T3	T3/T3	T-S
383	TUR_AnaB_002_T3r_2	MZ901375	BT	Anatolian Black	TUR	39.00/35.00	T-S	MDC-	T3r/T3j	T3/T3	T-S
384	TUR_AnaB_003_T3x_2	MZ901376	BT	Anatolian Black	TUR	39.00/35.00	T-S	MDC-	T3/T3	T3/T3	T-S
385	TUR_AnaB_004_T2x_2	MZ901377	BT	Anatolian Black	TUR	39.00/35.00	T-S	MDCT	T2/T2	T2/T2	T-S
386	ESP_Betz_001_T3l_1	EU177833	BT	Betizuak	ESP	43.25/-2.33	NCBI	MDC-	T3l/T3	T3/T3	(1)
387	ESP_Betz_002_T3l_1	EU177834	BT	Betizuak	ESP	43.25/-2.33	NCBI	MDCT	T3l/T3	T3/T3	(1)
388	GRC_Gree_001_T2x_1	EU177849	BT	Greek	GRC	39.07/21.82	NCBI	MDC-	T2/T2	T2/T2	(1)
389	GRC_Gree_002_T2d_1	EU177852	BT	Greek	GRC	39.07/21.82	NCBI	MDC-	T2d/T2d	T2/T2	(1)
390	IRN_Iran_001_T3x_1	EU177838	BT	Iranian	IRN	32.43/53.69	NCBI	MDCT	T3/T3	T3/T3	(1)
391	IRN_Iran_002_T3x_1	EU177839	BT	Iranian	IRN	32.43/53.69	NCBI	MDC-	T3/T3	T3/T3	(1)
392	IRN_Iran_003_T2b_1	EU177857	BT	Iranian	IRN	32.43/53.69	NCBI	MDC-	T2b/T2b	T2/T2	(1)
393	IRN_Iran_004_T2x_1	EU177858	BT	Iranian	IRN	32.43/53.69	NCBI	MDC-	T2/T2	T2/T2	(1)
394	IRN_Iran_005_T2x_1	EU177859	BT	Iranian	IRN	32.43/53.69	NCBI	MDC-	T2/T2	T2/T2	(1)
395	IRN_Iran_006_T2c_1	EU177860	BT	Iranian	IRN	32.43/53.69	NCBI	MDC-	T2c/T2	T2/T2	(1)
396	IRN_Iran_007_I2x_1	EU177870	BT	Iranian	IRN	32.43/53.69	NCBI	MDC-	I2/I2	I/I	(1)
397	IRQ_Iraq_001_T3x_1	EU177835	BT	Iraqi	IRQ	33.22/43.68	NCBI	MDC-	T3/T3	T3/T3	(1)
398	IRQ_Iraq_002_T3s_1	EU177836	BT	Iraqi	IRQ	33.22/43.68	NCBI	MDC-	T3s/T3s	T3/T3	(1)
399	IRQ_Iraq_003_T3k_1	EU177837	BT	Iraqi	IRQ	33.22/43.68	NCBI	MDC-	T3k/T3k	T3/T3	(1)
400	IRQ_Iraq_004_T1c_1	EU177848	BT	Iraqi	IRQ	33.22/43.68	NCBI	MDC-	T1c/T1c	T1/T1	(1)
401	IRQ_Iraq_005_T2b_1	EU177856	BT	Iraqi	IRQ	33.22/43.68	NCBI	MDC-	T2b/T2b	T2/T2	(1)

No.	Fasta name	Acc. no.	SP	Breed	ORG	La-Y/Lo-X	Source	MDCT	MTP M/D	Beast M/D	Author
402	IRQ_Iraq_006_T5b_1	EU177864	BT	Iraqi	IRQ	33.22/43.68	NCBI	MDC-	T5b/QT	T5/Q	(1)
403	IRQ_Iraq_007_T5b_1	EU177865	BT	Iraqi	IRQ	33.22/43.68	NCBI	MDC-	T5b/T5b	T5/T1	(1)
404	IRQ_Iraq_008_I1x_1	EU177868	BT	Iraqi	IRQ	33.22/43.68	NCBI	MDC-	I1/I	I/I	(1)
405	IRQ_Iraq_009_I2x_1	EU177869	BT	Iraqi	IRQ	33.22/43.68	NCBI	MDC-	I2/I2	I/I	(1)
406	ITA_Caba_001_T7x_1	EU177840	BT	Cabannina	ITA	44.32/8.40	NCBI	MDCT	T7/T7	T3/Q	(1)
407	ITA_Caba_002_T2x_1	EU177850	BT	Cabannina	ITA	44.32/8.40	NCBI	MDC-	T2/T2	T2/T2	(1)
408	ITA_Caba_003_T2d_1	EU177851	BT	Cabannina	ITA	44.32/8.40	NCBI	MDC-	T2d/T2d	T2/T2	(1)
409	ITA_Caba_004_Q1x_1	EU177866	BT	Cabannina	ITA	44.32/8.40	NCBI	MDC-	Q1/Q1	Q/Q	(1)
410	ITA_Caba_005_Q1x_1	EU177867	BT	Cabannina	ITA	44.32/8.40	NCBI	MDC-	Q1/Q1	Q/Q	(1)
411	ITA_Chia_001_T3r_1	EU177818	BT	Chianina	ITA	43.40/11.73	NCBI	MDC-	T3r/T3j	T3/T3	(1)
412	ITA_Chia_002_T3r_1	EU177819	BT	Chianina	ITA	43.40/11.73	NCBI	MDC-	T3r/T3j	T3/T3	(1)
413	ITA_Chia_003_T3r_1	EU177820	BT	Chianina	ITA	43.40/11.73	NCBI	MDC-	T3r/T3r	T3/T3	(1)
414	ITA_Chia_004_T3i_1	EU177822	BT	Chianina	ITA	43.40/11.73	NCBI	MDC-	T3i/T3	T3/T3	(1)
415	ITA_Chia_005_T3p_1	EU177825	BT	Chianina	ITA	43.40/11.73	NCBI	MDC-	T3p/T3p	T3/T3	(1)
416	ITA_Chia_006_T3q_1	EU177828	BT	Chianina	ITA	43.40/11.73	NCBI	MDC-	T3q/T3q	T3/T3	(1)
417	ITA_Chia_007_T1e_1	EU177841	BT	Chianina	ITA	43.40/11.73	NCBI	MDC-	T1e/T1b	T3/T1	(1)
418	ITA_Chia_008_T1a_1	EU177845	BT	Chianina	ITA	43.40/11.73	NCBI	MDC-	T1a/T1	T1/T1	(1)
419	ITA_Chia_009_T1a_1	EU177846	BT	Chianina	ITA	43.40/11.73	NCBI	MDC-	T1a/T1a	T1/T1	(1)
420	ITA_Chia_010_T2x_1	EU177853	BT	Chianina	ITA	43.40/11.73	NCBI	MDC-	T2/T2	T2/T2	(1)
421	ITA_Chia_011_T2x_1	EU177854	BT	Chianina	ITA	43.40/11.73	NCBI	MDC-	T2/T2	T2/T2	(1)
422	ITA_Chia_012_T2x_1	EU177855	BT	Chianina	ITA	43.40/11.73	NCBI	MDC-	T2/T2b	T2/T2	(1)
423	ITA_Chia_023_T3r_1	EU177816	BT	Chianina	ITA	43.40/11.73	NCBI	MDC-	T3r/T3	T3/T3	(1)
424	ITA_Cini_001_T3i_1	EU177823	BT	Cinisara	ITA	38.16/13.11	NCBI	MDC-	T3i/T3i	T3/T3	(1)
425	ITA_Cini_002_T1b_1	EU177842	BT	Cinisara	ITA	38.16/13.11	NCBI	MDC-	T1b/T1	T1/T1	(1)
426	ITA_Marm_001_T3p_1	EU177824	BT	Maremmana	ITA	42.66/11.09	NCBI	MDCT	T3p/T3p	T3/T3	(1)
427	ITA_Marm_002_T3j_1	EU177829	BT	Maremmana	ITA	42.66/11.09	NCBI	MDC-	T3j/T3j	T3/T3	(1)
428	ITA_Marm_003_T1a_1	EU177844	BT	Maremmana	ITA	42.66/11.09	NCBI	MDC-	T1a/T1	T1/T1	(1)
429	ITA_Modi_001_T3x_1	EU177831	BT	Modicana	ITA	36.91/14.68	NCBI	MDC-	T3/T3	T3/T3	(1)
430	ITA_Pett_001_T3x_1	EU177832	BT	Pettiazza	ITA	44.88/7.33	NCBI	MDC-	T3/T3	T3/T3	(1)

No.	Fasta name	Acc. no.	SP	Breed	ORG	La-Y/Lo-X	Source	MDCT	MTP M/D	Beast M/D	Author
431	ITA_Pied_001_T3r_1	EU177815	BT	Piedmontese	ITA	45.05/7.52	NCBI	MDC-	T3r/T3	T3/T3	(1)
432	ITA_Pied_002_T5a_1	EU177863	BT	Piedmontese	ITA	45.05/7.52	NCBI	MDCT	T5a/T5a	T5/Q	(1)
433	ITA_Podo_009_T3j_1	EU177830	BT	Italian Podolian	ITA	40.64/15.97	NCBI	MDC-	T3j/T3j	T3/T3	(1)
434	ITA_Podo_010_T1a_1	EU177843	BT	Italian Podolian	ITA	40.64/15.97	NCBI	MDC-	T1a/T1	T1/T1	(1)
435	ITA_Rend_001_T2c_1	EU177861	BT	Rendena	ITA	46.12/10.75	NCBI	MDC-	T2c/T2	T2/T2	(1)
436	ITA_Vald_001_T3r_1	EU177817	BT	Valdostana	ITA	45.74/7.43	NCBI	MDC-	T3r/T3d	T3/T3	(1)
437	ITA_Vald_002_T5a_1	EU177862	BT	Valdostana	ITA	45.74/7.43	NCBI	MDC-	T5a/T5a	T5/Q	(1)
438	NLD_Frie_001_T3r_1	EU177821	BT	Friesian	ITA	45.70/9.68	NCBI	MDC-	T3r/T3	T3/T3	(1)
439	NLD_Frie_002_T3q_1	EU177826	BT	Friesian	ITA	45.70/9.68	NCBI	MDCT	T3q/T3q	T3/T3	(1)
440	NLD_Frie_003_T3q_1	EU177827	BT	Friesian	ITA	45.70/9.68	NCBI	MDC-	T3q/T3q	T3/T3	(1)
441	NLD_Frie_004_T1c_1	EU177847	BT	Friesian	ITA	45.70/9.68	NCBI	MDC-	T1c/T1c	T1/T1	(1)
442	USA_BisA_001_RPQ_1	EU177871	BB	-	USA	44.66/-111.04	NCBI	-DCT	RPQ/T1b	-/outgroup	(1)
443	ITA_Ager_001_R1a_1	FJ971084	BT	Agerolese	ITA	40.64/14.55	NCBI	MDC-	R1a/R	R/R	(2)
444	ITA_Chia_013_Q2x_1	FJ971081	BT	Chianina	ITA	43.40/11.73	NCBI	MDC-	Q2/QT	Q/Q	(2)
445	ITA_Cini_003_R1a_1	FJ971085	BT	Cinisara	ITA	38.16/13.11	NCBI	MDC-	R1a/R	R/R	(2)
446	ITA_Cini_004_R1a_1	FJ971086	BT	Cinisara	ITA	38.16/13.11	NCBI	MDC-	R1a/R	R/R	(2)
447	ITA_RedP_001_Q1a_1	FJ971082	BT	Italian Red Pied	ITA	45.74/7.43	NCBI	MDC-	Q1a/Q1a	Q/T1	(2)
448	ITA_Roma_001_Q2x_1	FJ971080	BT	Romagnola	ITA	44.22/12.04	NCBI	MDC-	Q2/QT	Q/Q	(2)
449	ITA_Roma_002_Q1a_1	FJ971083	BT	Romagnola	ITA	44.22/12.04	NCBI	MDC-	Q1a/Q1a	Q/T1	(2)
450	ITA_Roma_003_R2x_1	FJ971087	BT	Romagnola	ITA	44.22/12.04	NCBI	MDC-	R2/R2	R/R	(2)
451	MNG_Taur_002_I1x_1	FJ971088	BT	-	MNG	46.86/103.85	NCBI	MDC-	I1/I	I/I	(2)
452	GBR_BRS_003_T3r_1	V00654	BT	-	GBR	52.21/0.12	NCBI	MDCT	T3r/T3	T3/T3	(3)
453	ITA_Caba_006_Q1x_1	HQ184038	BT	Cabannina	ITA	44.32/8.40	NCBI	MDC-	Q1/Q1	Q/Q	(4)
454	ITA_Chia_014_Q2x_1	HQ184030	BT	Chianina	ITA	43.40/11.73	NCBI	MDCT	Q2/QT	Q/Q	(4)
455	ITA_Chia_015_Q2x_1	HQ184031	BT	Chianina	ITA	43.40/11.73	NCBI	MDC-	Q2/QT	Q/T1	(4)
456	ITA_Chia_016_Q2x_1	HQ184032	BT	Chianina	ITA	43.40/11.73	NCBI	MDC-	Q2/QT	Q/T1	(4)
457	ITA_Chia_017_Q1x_1	HQ184039	BT	Chianina	ITA	43.40/11.73	NCBI	MDC-	Q1/Q1	Q/Q	(4)
458	ITA_GAlp_001_Q1x_1	HQ184036	BT	Grauvieh (Grey Alpine)	ITA	46.14/12.22	NCBI	MDC-	Q1/Q1	Q/Q	(4)
459	ITA_GAlp_002_Q1x_1	HQ184037	BT	Grauvieh (Grey Alpine)	ITA	46.14/12.22	NCBI	MDC-	Q1/Q1	Q/Q	(4)

No.	Fasta name	Acc. no.	SP	Breed	ORG	La-Y/Lo-X	Source	MDCT	MTP M/D	Beast M/D	Author
460	ITA_Marc_001_R2x_1	HQ184045	BT	Marchigiana	ITA	43.51/12.99	NCBI	MDCT	R2/R2	R/R	(4)
461	ITA_Roma_004_Q2x_1	HQ184033	BT	Romagnola	ITA	44.22/12.04	NCBI	MDC-	Q2/QT	Q/T1	(4)
462	ITA_Roma_005_Q1a_1	HQ184034	BT	Romagnola	ITA	44.22/12.04	NCBI	MDC-	Q1a/Q1a	Q/Q	(4)
463	ITA_Roma_006_Q1a_1	HQ184035	BT	Romagnola	ITA	44.22/12.04	NCBI	MDCT	Q1a/Q1a	Q/Q	(4)
464	ITA_Roma_007_R1x_1	HQ184040	BT	Romagnola	ITA	44.22/12.04	NCBI	MDCT	R1/R	R/R	(4)
465	ITA_Roma_008_R1x_1	HQ184041	BT	Romagnola	ITA	44.22/12.04	NCBI	MDC-	R1/R	R/R	(4)
466	ITA_Roma_009_R1x_1	HQ184042	BT	Romagnola	ITA	44.22/12.04	NCBI	MDC-	R1/R	R/R	(4)
467	ITA_Roma_010_R1x_1	HQ184043	BT	Romagnola	ITA	44.22/12.04	NCBI	MDC-	R1/R	R/R	(4)
468	ITA_Roma_011_R1x_1	HQ184044	BT	Romagnola	ITA	44.22/12.04	NCBI	MDC-	R1/R	R/R	(4)
469	EGY_Domi_001_T1d_1	JN817321	BT	Domiaty	EGY	31.04/30.47	NCBI	MDC-	T1d/T1d	T1/T1	(5)
470	EGY_Domi_002_T1c_1	JN817322	BT	Domiaty	EGY	31.04/30.47	NCBI	MDC-	T1c/T1c	T1/T1	(5)
471	EGY_Domi_003_T1c_1	JN817323	BT	Domiaty	EGY	31.04/30.47	NCBI	MDC-	T1c/T1c	T1/T1	(5)
472	EGY_Domi_004_T1b_1	JN817324	BT	Domiaty	EGY	31.04/30.47	NCBI	MDC-	T1b/T1b	T1/T1	(5)
473	EGY_Menf_001_T1c_1	JN817325	BT	Menofi	EGY	31.38/31.04	NCBI	MDC-	T1c/T1c	T1/T1	(5)
474	EGY_Menf_002_T1c_1	JN817326	BT	Menofi	EGY	31.38/31.04	NCBI	MDC-	T1c/T1c	T1/T1	(5)
475	EGY_Menf_003_T1b_1	JN817327	BT	Menofi	EGY	31.38/31.04	NCBI	MDC-	T1b/T1b	T3/T3	(5)
476	EGY_Menf_004_T1c_1	JN817328	BT	Menofi	EGY	31.38/31.04	NCBI	MDC-	T1c/T1c	T1/T1	(5)
477	EGY_Menf_005_T1f_1	JN817329	BT	Menofi	EGY	31.38/31.04	NCBI	MDC-	T1f/T1f	T1/T1	(5)
478	ETH_Abig_001_T1d_1	JN817298	BI	Abigar	ETH	9.15/40.49	NCBI	MDC-	T1d/T1	T1/T1	(5)
479	ETH_Arsi_001_T1b_1	JN817302	BI	Arsi	ETH	9.15/40.49	NCBI	MDCT	T1b/T1b	T1/T3	(5)
480	ETH_Arsi_002_T1a_1	JN817303	BI	Arsi	ETH	9.15/40.49	NCBI	MDC-	T1a/T1a	T1/T1	(5)
481	ETH_Arsi_003_T1d_1	JN817304	BI	Arsi	ETH	9.15/40.49	NCBI	MDC-	T1d/T1	T1/T1	(5)
482	ETH_Bora_001_T1d_1	JN817299	BI	Boran	ETH	9.15/40.49	NCBI	MDC-	T1d/T1	T1/T1	(5)
483	ETH_Bora_002_T1b_1	JN817305	BI	Boran	ETH	9.15/40.49	NCBI	MDC-	T1b/T1b	T1/T1	(5)
484	ETH_Horr_001_T1d_1	JN817330	BI	Horro	ETH	9.15/40.49	NCBI	MDC-	T1d/T1	T1/T1	(5)
485	ETH_Shek_001_T1b_1	JN817348	BT	Sheko	ETH	9.15/40.49	NCBI	MDC-	T1b/T1b	T1/T1	(5)
486	ETH_Shek_002_T1b_1	JN817349	BT	Sheko	ETH	9.15/40.49	NCBI	MDC-	T1b/T1b	T1/T1	(5)
487	FRA_Limo_001_T1a_1	JN817331	BT	Limousin	FRA	45.89/1.57	NCBI	MDC-	T1a/T1a	T1/T1	(5)
488	ITA_Ager_002_T1a_1	JN817340	BT	Agerolese	ITA	40.64/14.55	NCBI	MDC-	T1a/T1a	T1/T1	(5)

No.	Fasta name	Acc. no.	SP	Breed	ORG	La-Y/Lo-X	Source	MDCT	MTP M/D	Beast M/D	Author
489	ITA_Ager_003_T1a_1	JN817341	BT	Agerolese	ITA	40.64/14.55	NCBI	MDC-	T1a/T1	T1/T1	(5)
490	ITA_Ager_004_T1a_1	JN817342	BT	Agerolese	ITA	40.64/14.55	NCBI	MDC-	T1a/T1	T1/T1	(5)
491	ITA_Brow_001_T1a_1	JN817312	BT	Italian Brown	ITA	45.72/11.36	NCBI	MDC-	T1a/T1a	T1/T1	(5)
492	ITA_Calv_001_T1e_1	JN817306	BT	Calvana	ITA	44.05/11.12	NCBI	MDC-	T1e/T1b	T3/T1	(5)
493	ITA_Chia_018_T1a_1	JN817313	BT	Chianina	ITA	43.40/11.73	NCBI	MDC-	T1a/T1	T1/T1	(5)
494	ITA_Chia_019_T1a_1	JN817314	BT	Chianina	ITA	43.40/11.73	NCBI	MDC-	T1a/T1	T1/T1	(5)
495	ITA_Chia_020_T1a_1	JN817315	BT	Chianina	ITA	43.40/11.73	NCBI	MDC-	T1a/T1	T1/T1	(5)
496	ITA_Chia_021_T1a_1	JN817316	BT	Chianina	ITA	43.40/11.73	NCBI	MDC-	T1a/T1	T1/T1	(5)
497	ITA_Chia_022_T1b_1	JN817350	BT	Chianina	ITA	43.40/11.73	NCBI	MDC-	T1b/T1	T1/T1	(5)
498	ITA_Cini_005_T1a_1	JN817317	BT	Cinisara	ITA	38.16/13.11	NCBI	MDC-	T1a/T1a	T1/T1	(5)
499	ITA_Cini_006_T1a_1	JN817318	BT	Cinisara	ITA	38.16/13.11	NCBI	MDC-	T1a/T1	T1/T1	(5)
500	ITA_Cini_007_T1c_1	JN817319	BT	Cinisara	ITA	38.16/13.11	NCBI	MDC-	T1c/T1b	T3/T1	(5)
501	ITA_Cini_008_T1b_1	JN817320	BT	Cinisara	ITA	38.16/13.11	NCBI	MDC-	T1b/T1	T1/T1	(5)
502	ITA_Marc_002_T1b_1	JN817334	BT	Marchigiana	ITA	43.51/12.99	NCBI	MDC-	T1b/T1	T1/T1	(5)
503	ITA_Marc_003_T1a_1	JN817335	BT	Marchigiana	ITA	43.51/12.99	NCBI	MDC-	T1a/T1	T1/T1	(5)
504	ITA_Marc_004_T1a_1	JN817336	BT	Marchigiana	ITA	43.51/12.99	NCBI	MDC-	T1a/T1	T1/T1	(5)
505	ITA_Marc_005_T1a_1	JN817337	BT	Marchigiana	ITA	43.51/12.99	NCBI	MDC-	T1a/T1a	T1/T1	(5)
506	ITA_Marc_006_T1a_1	JN817338	BT	Marchigiana	ITA	43.51/12.99	NCBI	MDC-	T1a/T1a	T1/T1	(5)
507	ITA_Marc_007_T1a_1	JN817339	BT	Marchigiana	ITA	43.51/12.99	NCBI	MDC-	T1a/T1a	T1/T1	(5)
508	ITA_Marc_008_T1b_1	JN817351	BT	Marchigiana	ITA	43.51/12.99	NCBI	MDC-	T1b/T1	T1/T1	(5)
509	ITA_Marm_004_T1a_1	JN817332	BT	Maremmana	ITA	42.66/11.09	NCBI	MDC-	T1a/T1	T1/T1	(5)
510	ITA_Marm_005_T1a_1	JN817333	BT	Maremmana	ITA	42.66/11.09	NCBI	MDC-	T1a/T1	T1/T1	(5)
511	ITA_Podo_011_T1f_1	JN817343	BT	Italian Podolian	ITA	40.64/15.97	NCBI	MDCT	T1f/T1f	T1/T1	(5)
512	ITA_Podo_012_T1a_1	JN817344	BT	Italian Podolian	ITA	40.64/15.97	NCBI	MDC-	T1a/T1	T1/T1	(5)
513	ITA_Roma_012_T1a_1	JN817345	BT	Romagnola	ITA	44.22/12.04	NCBI	MDC-	T1a/T1	T1/T1	(5)
514	ITA_Roma_013_T1c_1	JN817346	BT	Romagnola	ITA	44.22/12.04	NCBI	MDC-	T1c/T1c	T1/T1	(5)
515	ITA_Roma_014_T1a_1	JN817347	BT	Romagnola	ITA	44.22/12.04	NCBI	MDC-	T1a/T1	T1/T1	(5)
516	MEX_Creo_001_T1c_1	JN817307	BT	Creole	MEX	26.04/-111.67	NCBI	MDC-	T1c/T1c	T1/T1	(5)
517	MEX_Creo_002_T1a_1	JN817308	BT	Chihuahua Creole	MEX	28.63/-106.07	NCBI	MDC-	T1a/T1a	T1/T1	(5)

No.	Fasta name	Acc. no.	SP	Breed	ORG	La-Y/Lo-X	Source	MDCT	MTP M/D	Beast M/D	Author
518	PRT_Alen_001_T1c_1	JN817300	BT	Alentejana	PRT	38.02/-7.86	NCBI	MDC-	T1c/T1c	T1/T1	(5)
519	PRT_Alen_002_T1c_1	JN817301	BT	Alentejana	PRT	38.02/-7.86	NCBI	MDC-	T1c/T1c	T1/T1	(5)
520	PRY_Creo_003_T1c_1	JN817309	BT	Pampa Chaqueno Creole	URY	-33,67/-54,21	NCBI	MDC-	T1c/T1c	T1/T1	(5)
521	PRY_Creo_004_T1c_1	JN817310	BT	Pampa Chaqueno Creole	URY	-33,67/-54,21	NCBI	MDC-	T1c/T1c	T1/T1	(5)
522	PRY_Creo_005_T1c_1	JN817311	BT	Pampa Chaqueno Creole	URY	-33,67/-54,21	NCBI	MDC-	T1c/T1c	T1/T1	(5)
523	DNK_Prim_001_P1_1_600	MF169211	BP	-	DNK	55.68/12.57	NCBI	MDCT	P1/PQT	P2/P	(6)
524	DNK_Prim_002_P1_1_600	MF169212	BP	-	DNK	55.68/12.57	NCBI	MDCT	P1/PQT	P2/P	(6)
525	DNK_Prim_003_P1_1_600	MF169213	BP	-	DNK	55.68/12.57	NCBI	MDCT	P1/T3d	P3/T3	(6)
526	KOR_Kore_001_T1c_1	AY526085	BT	Korean native	KOR	35.91/127.77	NCBI	MDC-	T1c/T3a	T4/T3	(7)
527	KOR_Nati_001_T1c_1	NC_006853	BT	Korean native	KOR	35.91/127.77	NCBI	MDCT	T1c/T3a	T4/T3	(7)
528	ITA_RedP_002_T3s_1	JQ967333	BT	Italian Red Pied	ITA	45.74/7.43	NCBI	MDC-	T3s/T3s	T3/T3	(8)
529	GBR_AngM_001_T3d_1	GU947019	BT	Angus mix	GBR	57.15/-2.09	NCBI	MDC-	T3d/T3	T3/T3	(9)
530	GBR_AngM_002_T1a_1	GU947020	BT	Angus mix	GBR	57.15/-2.09	NCBI	MDC-	T1a/T1a	T1/T1	(9)
531	GBR_Long_001_T3g_1	GU947021	BT	Longhorn	USA	31.97/-99.90	NCBI	MDC-	T3g/T3g	T3/T3	(9)
532	USA_Hybr_001_T3f_1	GU947007	BT	bison-cattle	USA	-	NCBI	---T	T1b/T3f	-/outgroup	(9)
533	USA_BisA_002_RPQ_1	GU946976	BB	-	USA	47.37/-114.26	NCBI	-DCT	RPQ/T1b	-/outgroup	(9)
534	GBR_Prim_004_P1x_1_6738	GU985279	BP	-	GBR	53.08/-1.64	NCBI	MDCT	P1/P1	P1/P	(10)
535	DEU_VieF_001_T3e_1	AF492351	BT	Simmental	DEU	48.44/12.57	NCBI	MDC-	T3e/T3e	T3/T3	(11)
536	LKA_Zwer_001_I2x_1	AF492350	BI	Zwergzebu	LKA	7.87/80.77	NCBI	MDCT	I2/I2	I/I	(11)
537	AFR_Ngun_001_T1b_1	KF163061	BT	Nguni	ZAF	-33,47/18,72	NCBI	MDCT	T1b/T1	T1/T1	(12)
538	AFR_Ngun_002_T1b_1	KF163062	BT	Nguni	ZAF	-33,47/18,72	NCBI	MDC-	T1b/T1b	T1/T1	(12)
539	AFR_Ngun_003_T1b_1	KF163063	BT	Nguni	ZAF	-33,47/18,72	NCBI	MDC-	T1b/T1	T1/T1	(12)
540	AFR_Ngun_004_T1b_1	KF163064	BT	Nguni	ZAF	-33,47/18,72	NCBI	MDC-	T1b/T1b	T1/T1	(12)
541	AFR_Ngun_005_T1b_1	KF163065	BT	Nguni	ZAF	-33,47/18,72	NCBI	MDC-	T1b/T1b	T1/T1	(12)
542	AFR_Ngun_006_T1b_1	KF163066	BT	Nguni	ZAF	-33,47/18,72	NCBI	MDC-	T1b/T1b	T1/T1	(12)
543	AFR_Ngun_007_T1b_1	KF163067	BT	Nguni	ZAF	-33,47/18,72	NCBI	MDC-	T1b/T1b	T1/T1	(12)
544	AFR_Ngun_008_T1b_1	KF163068	BT	Nguni	ZAF	-33,47/18,72	NCBI	MDC-	T1b/T1b	T1/T1	(12)
545	AFR_Ngun_009_T1b_1	KF163069	BT	Nguni	ZAF	-33,47/18,72	NCBI	MDC-	T1b/T1b	T1/T1	(12)
546	AFR_Ngun_010_T1b_1	KF163070	BT	Nguni	ZAF	-33,47/18,72	NCBI	MDC-	T1b/T1b	T1/T1	(12)

No.	Fasta name	Acc. no.	SP	Breed	ORG	La-Y/Lo-X	Source	MDCT	MTP M/D	Beast M/D	Author
547	AFR_Ngun_011_T1b_1	KF163071	BT	Nguni	ZAF	-33,47/18,72	NCBI	MDC-	T1b/T1b	T1/T1	(12)
548	AFR_Ngun_012_T1c_1	KF163072	BT	Nguni	ZAF	-33,47/18,72	NCBI	MDC-	T1c/T1c	T1/T1	(12)
549	AFR_Ngun_013_T1b_1	KF163073	BT	Nguni	ZAF	-33,47/18,72	NCBI	MDC-	T1b/T1	T1/T1	(12)
550	AFR_Ngun_014_T1b_1	KF163074	BT	Nguni	ZAF	-33,47/18,72	NCBI	MDC-	T1b/T1b	T1/T1	(12)
551	AFR_Ngun_015_T1b_1	KF163075	BT	Nguni	ZAF	-33,47/18,72	NCBI	MDC-	T1b/T1b	T1/T1	(12)
552	AFR_Ngun_016_T1b_1	KF163076	BT	Nguni	ZAF	-33,47/18,72	NCBI	MDC-	T1b/T1	T1/T1	(12)
553	AFR_Ngun_017_T1b_1	KF163077	BT	Nguni	ZAF	-33,47/18,72	NCBI	MDC-	T1b/T1b	T1/T1	(12)
554	AFR_Ngun_018_T1b_1	KF163078	BT	Nguni	ZAF	-33,47/18,72	NCBI	MDC-	T1b/T1b	T1/T1	(12)
555	AFR_Ngun_019_T1b_1	KF163079	BT	Nguni	ZAF	-33,47/18,72	NCBI	MDC-	T1b/T1b	T1/T1	(12)
556	AFR_Ngun_020_T1b_1	KF163080	BT	Nguni	ZAF	-33,47/18,72	NCBI	MDC-	T1b/T1b	T1/T1	(12)
557	AFR_Ngun_021_T1d_1	KF163081	BT	Nguni	ZAF	-33,47/18,72	NCBI	MDC-	T1d/T1d	T1/T1	(12)
558	AFR_Ngun_022_T1g_1	KF163082	BT	Nguni	ZAF	-33,47/18,72	NCBI	MDC-	T1g/T1g	T1/T1	(12)
559	AFR_Ngun_023_T1b_1	KF163083	BT	Nguni	ZAF	-33,47/18,72	NCBI	MDC-	T1b/T1b	T1/T1	(12)
560	AFR_Ngun_024_T1b_1	KF163084	BT	Nguni	ZAF	-33,47/18,72	NCBI	MDC-	T1b/T1b	T1/T1	(12)
561	AFR_Ngun_025_T1b_1	KF163085	BT	Nguni	ZAF	-33,47/18,72	NCBI	MDC-	T1b/T1b	T1/T1	(12)
562	AFR_Ngun_026_T1b_1	KF163086	BT	Nguni	ZAF	-33,47/18,72	NCBI	MDC-	T1b/T1b	T1/T1	(12)
563	AFR_Ngun_027_T1b_1	KF163087	BT	Nguni	ZAF	-33,47/18,72	NCBI	MDC-	T1b/T1	T1/T1	(12)
564	AFR_Ngun_028_T1b_1	KF163088	BT	Nguni	ZAF	-33,47/18,72	NCBI	MDC-	T1b/T1b	T1/T1	(12)
565	AFR_Ngun_029_T1b_1	KF163089	BT	Nguni	ZAF	-33,47/18,72	NCBI	MDC-	T1b/T1b	T1/T1	(12)
566	AFR_Ngun_030_T1b_1	KF163090	BT	Nguni	ZAF	-33,47/18,72	NCBI	MDC-	T1b/T1b	T1/T1	(12)
567	AFR_Ngun_031_T1b_1	KF163091	BT	Nguni	ZAF	-33,47/18,72	NCBI	MDC-	T1b/T1b	T1/T1	(12)
568	AFR_Ngun_032_T1b_1	KF163092	BT	Nguni	ZAF	-33,47/18,72	NCBI	MDC-	T1b/T1	T1/T1	(12)
569	AFR_Ngun_033_T1g_1	KF163093	BT	Nguni	ZAF	-33,47/18,72	NCBI	MDC-	T1g/T1g	T1/T1	(12)
570	AFR_Ngun_034_T1b_1	KF163094	BT	Nguni	ZAF	-33,47/18,72	NCBI	MDC-	T1b/T1b	T1/T1	(12)
571	KOR_Hanw_001_T2x_1	HQ025805	BT	Hanwoo	KOR	35.91/127.77	NCBI	MDC-	T2/T2	T2/T2	(13)
572	POL_Prim_003_P1x_1_1500	JQ437479	BP	-	POL	53.63/21.81	NCBI	MDCT	P1/P1	P3/P	(14)
573	CHN_Taur_001_I1x_1	KP143771	BT	-	CHN	35.86/104.20	NCBI	MDC-	I1/I	I/I	(15)
574	SPA_Pire_001_Q1x_1	KP637147	BT	Pirenaica	ESP	42.99/-2.62	NCBI	MDC-	Q1/QT	Q/T1	(16)
575	DEU_RMou_001_T3r_1	KJ709681	BT	Red Mountain	DEU	49.63/7.91	NCBI	MDC-	T3r/T3	T3/T3	(17)

No.	Fasta name	Acc. no.	SP	Breed	ORG	La-Y/Lo-X	Source	MDCT	MTP M/D	Beast M/D	Author
576	DEU_RMou_002_T3r_1	KJ709682	BT	Red Mountain	DEU	49.63/7.91	NCBI	MDC-	T3r/T3	T3/T3	(17)
577	DEU_RMou_003_T3x_1	KJ709683	BT	Red Mountain	DEU	49.63/7.91	NCBI	MDC-	T3/T3	T3/T3	(17)
578	DEU_RMou_004_T3x_1	KJ709684	BT	Red Mountain	DEU	49.63/7.91	NCBI	MDC-	T3/T3r	T3/T3	(17)
579	DEU_RMou_005_T3r_1	KJ709685	BT	Red Mountain	DEU	49.63/7.91	NCBI	MDC-	T3r/T3	T3/T3	(17)
580	DEU_RMou_006_T3x_1	KJ709686	BT	Red Mountain	DEU	49.63/7.91	NCBI	MDC-	T3/T1e	T3/T3	(17)
581	GBR_WPar_001_T3x_1	KC153972	BT	White Park	GBR	53.96/-1.09	NCBI	MDC-	T3/T3	T3/T3	(17)
582	GBR_WPar_002_T1e_1	KC153973	BT	White Park	GBR	53.96/-1.09	NCBI	MDC-	T1e/T1e	T3/T3	(17)
583	GBR_WPar_003_T3e_1	KC153974	BT	White Park	GBR	53.96/-1.09	NCBI	MDC-	T3e/T3e	T3/T3	(17)
584	GBR_WPar_004_T1e_1	KC153975	BT	White Park	GBR	53.96/-1.09	NCBI	MDCT	T1e/T1e	T3/T3	(17)
585	GBR_WPar_005_T3x_1	KC153976	BT	White Park	GBR	53.96/-1.09	NCBI	MDCT	T3/T3	T3/T3	(17)
586	GBR_WPar_006_T3r_1	KC153977	BT	White Park	GBR	53.96/-1.09	NCBI	MDC-	T3r/T3r	T3/T3	(17)
587	JPN_JaBl_001_T4x_1	AB074962	BT	Japanese Black	JPN	36.20/138.25	NCBI	MDC-	T4/T3a	T4/T3	(18)
588	JPN_JaBl_002_T4a_1	AB074963	BT	Japanese Black	JPN	36.20/138.25	NCBI	MDCT	T4a/T3a	T4/T3	(18)
589	JPN_JaBl_003_T4x_1	AB074964	BT	Japanese Black	JPN	36.20/138.25	NCBI	MDC-	T4/T3	T4/T3	(18)
590	JPN_JaBl_004_T3k_1	AB074965	BT	Japanese Black	JPN	36.20/138.25	NCBI	MDC-	T3k/T3k	T3/T3	(18)
591	JPN_JaBl_005_T3k_1	AB074966	BT	Japanese Black	JPN	36.20/138.25	NCBI	MDC-	T3k/T3k	T3/T3	(18)
592	JPN_JaBl_006_T3n_1	AB074967	BT	Japanese Black	JPN	36.20/138.25	NCBI	MDC-	T3n/T3n	T3/T3	(18)
593	JPN_JaBl_007_T3n_1	AB074968	BT	Japanese Black	JPN	36.20/138.25	NCBI	MDC-	T3n/T3n	T3/T3	(18)
594	FRA_BisP_003_RPQ_1_15880	NC_027233	BPc	-	FRA	43.03/1.21	NCBI	-DCT	RPQ/P1	-/outgroup	(19)
595	FRA_BisP_005_RPQ_1_37700	KX898014	BPc	-	FRA	43.93/2.14	NCBI	-DCT	RPQ/P1	-/outgroup	(20)
596	RUS_BisP_006_RPQ_1_27800	KX898018	BPc	-	RUS	68.74/161.35	NCBI	-DCT	RPQ/P1	-/outgroup	(20)
597	RUS_BisP_007_RPQ_1_26650	KX898019	BPc	-	RUS	68.64/161.35	NCBI	-DCT	RPQ/P1	-/outgroup	(20)
598	IND_Nell_001_I1x_1	AY126697	BI	Nellore	IND	14.44/79.99	NCBI	MDCT	I1/I	I/I	(21)
599	IND_Nell_002_I1x_1	NC_005971	BI	Nellore	IND	14.44/79.99	NCBI	MDC-	I1/I	I/I	(21)
600	EGY_Domi_005_T3x_1	KT184452	BT	Domiaty	EGY	31.04/30.47	NCBI	MDC-	T3/T3	T3/T3	(22)
601	EGY_Domi_006_T2d_1	KT184456	BT	Domiaty	EGY	31.04/30.47	NCBI	MDC-	T2d/T2d	T2/T2	(22)
602	EGY_Domi_007_T2x_1	KT184457	BT	Domiaty	EGY	31.04/30.47	NCBI	MDC-	T2/T2	T2/T2	(22)
603	EGY_Domi_008_T2x_1	KT184460	BT	Domiaty	EGY	31.04/30.47	NCBI	MDC-	T2/T2	T2/T2	(22)
604	EGY_Domi_009_T1a_1	KT184462	BT	Domiaty	EGY	31.04/30.47	NCBI	MDC-	T1a/T1	T1/T1	(22)

No.	Fasta name	Acc. no.	SP	Breed	ORG	La-Y/Lo-X	Source	MDCT	MTP M/D	Beast M/D	Author
605	EGY_Domi_010_T1a_1	KT184463	BT	Domiaty	EGY	31.04/30.47	NCBI	MDCT	T1a/T1d	T1/T1	(22)
606	EGY_Domi_011_T1b_1	KT184469	BT	Domiaty	EGY	31.04/30.47	NCBI	MDC-	T1b/T1b	T1/T1	(22)
607	EGY_Domi_012_T1c_1	KT184470	BT	Domiaty	EGY	31.04/30.47	NCBI	MDC-	T1c/T1b	T3/T1	(22)
608	EGY_Domi_013_Q1x_1	KT184471	BT	Domiaty	EGY	31.04/30.47	NCBI	MDC-	Q1/Q	Q/T3	(22)
609	EGY_Domi_014_Q1x_1	KT184472	BT	Domiaty	EGY	31.04/30.47	NCBI	MDC-	Q1/Q	Q/T1	(22)
610	EGY_Menf_006_T3x_1	KT184451	BT	Menofi	EGY	31.38/31.04	NCBI	MDC-	T3/T3	T3/T3	(22)
611	EGY_Menf_007_T3o_1	KT184453	BT	Menofi	EGY	31.38/31.04	NCBI	MDC-	T3o/T3o	T3/T3	(22)
612	EGY_Menf_008_T3s_1	KT184454	BT	Menofi	EGY	31.38/31.04	NCBI	MDC-	T3s/T3i	T3/T3	(22)
613	EGY_Menf_009_T3x_1	KT184455	BT	Menofi	EGY	31.38/31.04	NCBI	MDC-	T3/T3r	T3/T3	(22)
614	EGY_Menf_010_T2x_1	KT184458	BT	Menofi	EGY	31.38/31.04	NCBI	MDC-	T2/T2	T2/T2	(22)
615	EGY_Menf_011_T2x_1	KT184459	BT	Menofi	EGY	31.38/31.04	NCBI	MDC-	T2/T2	T2/T2	(22)
616	EGY_Menf_012_T2x_1	KT184461	BT	Menofi	EGY	31.38/31.04	NCBI	MDC-	T2/T2	T2/T2	(22)
617	EGY_Menf_013_T1a_1	KT184464	BT	Menofi	EGY	31.38/31.04	NCBI	MDC-	T1a/T1d	T1/T1	(22)
618	EGY_Menf_014_T1a_1	KT184465	BT	Menofi	EGY	31.38/31.04	NCBI	MDC-	T1a/T1a	T1/T1	(22)
619	EGY_Menf_015_T1a_1	KT184466	BT	Menofi	EGY	31.38/31.04	NCBI	MDC-	T1a/T1	T1/T1	(22)
620	EGY_Menf_016_T1b_1	KT184467	BT	Menofi	EGY	31.38/31.04	NCBI	MDC-	T1b/T1	T1/T1	(22)
621	EGY_Menf_017_T1b_1	KT184468	BT	Menofi	EGY	31.38/31.04	NCBI	MDC-	T1b/T1	T1/T1	(22)
622	DEU_VieG_003_T3x_1	AY676860	BT	Gelbvieh (German Yellow)	DEU	48.79/11.50	NCBI	MDC-	T3/T3	T3/T3	(23)
623	FRA_Char_001_T3r_1	AY676858	BT	Charolais	FRA	46.44/4.28	NCBI	MDC-	T3r/T3r	T3/T3	(23)
624	FRA_Char_002_T3d_1	AY676861	BT	Charolais	FRA	46.44/4.28	NCBI	MDC-	T3d/T3d	T3/T3	(23)
625	FRA_Limo_002_T2x_1	AY676856	BT	Limousin	FRA	45.89/1.57	NCBI	MDC-	T2/T2	T2/T2	(23)
626	GBR_Angu_001_T3n_1	AY676857	BT	Angus	GBR	57.15/-2.09	NCBI	MDC-	T3n/T3n	T3/T3	(23)
627	GBR_Angu_002_T3x_1	AY676859	BT	Angus	GBR	57.15/-2.09	NCBI	MDC-	T3/T3	T3/T3	(23)
628	GBR_AngX_001_T3r_1	AY676862	BT	Angus X	GBR	57.15/-2.09	NCBI	MDC-	T3r/T3d	T3/T3	(23)
629	GBR_AngX_002_T3r_1	AY676863	BT	Angus X	GBR	57.15/-2.09	NCBI	MDC-	T3r/T3	T3/T3	(23)
630	GBR_AngX_003_T3g_1	AY676864	BT	Angus X	GBR	57.15/-2.09	NCBI	MDC-	T3g/T3	T3/T3	(23)
631	GBR_AngX_004_T3r_1	AY676865	BT	Angus X	GBR	57.15/-2.09	NCBI	MDC-	T3r/T3p	T3/T3	(23)
632	GBR_AngX_005_T3r_1	AY676866	BT	Angus X	GBR	57.15/-2.09	NCBI	MDC-	T3r/T3r	T3/T3	(23)
633	GBR_AngX_006_T6x_1	AY676867	BT	Angus X	GBR	57.15/-2.09	NCBI	MDCT	T6/T6	T3/T1	(23)

No.	Fasta name	Acc. no.	SP	Breed	ORG	La-Y/Lo-X	Source	MDCT	MTP M/D	Beast M/D	Author
634	GBR_AngX_007_T3r_1	AY676868	BT	Angus X	GBR	57.15/-2.09	NCBI	MDC-	T3r/T3r	T3/T3	(23)
635	GBR_AngX_008_T3r_1	AY676869	BT	Angus X	GBR	57.15/-2.09	NCBI	MDC-	T3r/T3r	T3/T3	(23)
636	GBR_AngX_009_T6x_1	AY676870	BT	Angus X	GBR	57.15/-2.09	NCBI	MDC-	T6/T6	T3/T1	(23)
637	GBR_AngX_010_T3d_1	AY676871	BT	Angus X	GBR	57.15/-2.09	NCBI	MDC-	T3d/T3	T3/T3	(23)
638	GBR_AngX_011_T3r_1	AY676872	BT	Angus X	GBR	57.15/-2.09	NCBI	MDC-	T3r/T3j	T3/T3	(23)
639	GBR_AngX_012_T3o_1	AY676873	BT	Angus X	GBR	57.15/-2.09	NCBI	MDC-	T3o/T3o	T3/T3	(23)
640	CHE_Simm_004_T3x_1	AY676855	BT	Simmental	CHE	46.63/7.85	NCBI	MDC-	T3/T3	T3/T3	(23)
641	KOR_Beef_001_T3x_1	DQ124387	BT	Beef cattle	KOR	35.91/127.77	NCBI	MDC-	T3/T3	T3/T3	(24)
642	KOR_Beef_002_T3o_1	DQ124388	BT	Beef cattle	KOR	35.91/127.77	NCBI	MDC-	T3o/T3o	T3/T3	(24)
643	KOR_Beef_003_T3h_1	DQ124390	BT	Beef cattle	KOR	35.91/127.77	NCBI	MDC-	T3h/T3	T3/T3	(24)
644	KOR_Beef_004_T3n_1	DQ124391	BT	Beef cattle	KOR	35.91/127.77	NCBI	MDC-	T3n/T3n	T3/T3	(24)
645	KOR_Beef_005_T4x_1	DQ124392	BT	Beef cattle	KOR	35.91/127.77	NCBI	MDC-	T4/T3a	T4/T3	(24)
646	KOR_Beef_006_T2a_1	DQ124393	BT	Beef cattle	KOR	35.91/127.77	NCBI	MDC-	T2a/T2	T2/T2	(24)
647	KOR_Beef_007_T3o_1	DQ124394	BT	Beef cattle	KOR	35.91/127.77	NCBI	MDC-	T3o/T3o	T3/T3	(24)
648	KOR_Beef_008_T3x_1	DQ124395	BT	Beef cattle	KOR	35.91/127.77	NCBI	MDC-	T3/T3	T3/T3	(24)
649	KOR_Beef_009_T2a_1	DQ124396	BT	Beef cattle	KOR	35.91/127.77	NCBI	MDC-	T2a/T2	T2/T2	(24)
650	KOR_Beef_010_T3f_1	DQ124397	BT	Beef cattle	KOR	35.91/127.77	NCBI	MDC-	T3f/T3n	T3/T3	(24)
651	KOR_Beef_011_T3o_1	DQ124398	BT	Beef cattle	KOR	35.91/127.77	NCBI	MDC-	T3o/T3o	T3/T3	(24)
652	KOR_Beef_012_T1b_1	DQ124399	BT	Beef cattle	KOR	35.91/127.77	NCBI	MDC-	T1b/T1b	T1/T1	(24)
653	KOR_Beef_013_T4x_1	DQ124400	BT	Beef cattle	KOR	35.91/127.77	NCBI	MDC-	T4/T3a	T4/T3	(24)
654	KOR_Beef_014_T4x_1	DQ124401	BT	Beef cattle	KOR	35.91/127.77	NCBI	MDC-	T4/T3a	T4/T3	(24)
655	KOR_Beef_015_T3o_1	DQ124402	BT	Beef cattle	KOR	35.91/127.77	NCBI	MDC-	T3o/T3o	T3/T3	(24)
656	KOR_Beef_016_P2x_1	DQ124389	BT	Beef cattle	KOR	35.91/127.77	NCBI	MDC-	P2/P2	P2/P	(24)
657	KOR_Kore_002_T3a_1	DQ124371	BT	Korean cattle	KOR	35.91/127.77	NCBI	MDC-	T3a/T3a	T3/T3	(24)
658	KOR_Kore_003_T4a_1	DQ124372	BT	Korean cattle	KOR	35.91/127.77	NCBI	MDC-	T4a/T3a	T4/T3	(24)
659	KOR_Kore_004_T3n_1	DQ124373	BT	Korean cattle	KOR	35.91/127.77	NCBI	MDC-	T3n/T3n	T3/T3	(24)
660	KOR_Kore_005_T3o_1	DQ124374	BT	Korean cattle	KOR	35.91/127.77	NCBI	MDC-	T3o/T3o	T3/T3	(24)
661	KOR_Kore_006_T4a_1	DQ124375	BT	Korean cattle	KOR	35.91/127.77	NCBI	MDC-	T4a/T4a	T4/T3	(24)
662	KOR_Kore_007_T3k_1	DQ124376	BT	Korean cattle	KOR	35.91/127.77	NCBI	MDC-	T3k/T3k	T3/T3	(24)

No.	Fasta name	Acc. no.	SP	Breed	ORG	La-Y/Lo-X	Source	MDCT	MTP M/D	Beast M/D	Author
663	KOR_Kore_008_T4x_1	DQ124377	BT	Korean cattle	KOR	35.91/127.77	NCBI	MDC-	T4/T4	T4/T3	(24)
664	KOR_Kore_009_T3n_1	DQ124378	BT	Korean cattle	KOR	35.91/127.77	NCBI	MDC-	T3n/T3n	T3/T3	(24)
665	KOR_Kore_010_T3n_1	DQ124379	BT	Korean cattle	KOR	35.91/127.77	NCBI	MDC-	T3n/T3n	T3/T3	(24)
666	KOR_Kore_011_T3o_1	DQ124380	BT	Korean cattle	KOR	35.91/127.77	NCBI	MDC-	T3o/T3o	T3/T3	(24)
667	KOR_Kore_012_T3o_1	DQ124381	BT	Korean cattle	KOR	35.91/127.77	NCBI	MDC-	T3o/T3o	T3/T3	(24)
668	KOR_Kore_013_T3o_1	DQ124382	BT	Korean cattle	KOR	35.91/127.77	NCBI	MDC-	T3o/T3o	T3/T3	(24)
669	KOR_Kore_014_T2x_1	DQ124383	BT	Korean cattle	KOR	35.91/127.77	NCBI	MDC-	T2/T2	T2/T2	(24)
670	KOR_Kore_015_T3q_1	DQ124384	BT	Korean cattle	KOR	35.91/127.77	NCBI	MDC-	T3q/T3q	T3/T3	(24)
671	KOR_Kore_016_T3b_1	DQ124385	BT	Korean cattle	KOR	35.91/127.77	NCBI	MDC-	T3b/T3b	T3/T3	(24)
672	KOR_Kore_017_T3k_1	DQ124386	BT	Korean cattle	KOR	35.91/127.77	NCBI	MDC-	T3k/T3a	T3/T3	(24)
673	NLD_HoFr_001_Ixx_1	DQ124403	BT	Holstein	NLD	52.52/4.79	NCBI	MDC-	I/T3a	I/T3	(24)
674	NLD_HoFr_002_T3x_1	DQ124404	BT	Holstein	NLD	52.52/4.79	NCBI	MDC-	T3/T3	T3/T3	(24)
675	NLD_HoFr_003_T3x_1	DQ124405	BT	Holstein	NLD	52.52/4.79	NCBI	MDC-	T3/T3	T3/T3	(24)
676	NLD_HoFr_004_T3x_1	DQ124406	BT	Holstein	NLD	52.52/4.79	NCBI	MDC-	T3/T3c	T3/T3	(24)
677	NLD_HoFr_005_T3o_1	DQ124407	BT	Holstein	NLD	52.52/4.79	NCBI	MDC-	T3o/T3	T3/T3	(24)
678	NLD_HoFr_006_T3r_1	DQ124408	BT	Holstein	NLD	52.52/4.79	NCBI	MDC-	T3r/T3	T3/T3	(24)
679	NLD_HoFr_007_T3c_1	DQ124409	BT	Holstein	NLD	52.52/4.79	NCBI	MDC-	T3c/T3	T3/T3	(24)
680	NLD_HoFr_008_T3c_1	DQ124410	BT	Holstein	NLD	52.52/4.79	NCBI	MDC-	T3c/T3c	T3/T3	(24)
681	NLD_HoFr_009_T3h_1	DQ124411	BT	Holstein	NLD	52.52/4.79	NCBI	MDC-	T3h/T3	T3/T3	(24)
682	NLD_HoFr_010_T4x_1	DQ124412	BT	Holstein	NLD	52.52/4.79	NCBI	MDC-	T4/T4	T4/T3	(24)
683	NLD_HoFr_011_T3r_1	DQ124413	BT	Holstein	NLD	52.52/4.79	NCBI	MDC-	T3r/T3r	T3/T3	(24)
684	NLD_HoFr_012_T3x_1	DQ124414	BT	Holstein	NLD	52.52/4.79	NCBI	MDC-	T3/T3	T3/T3	(24)
685	NLD_HoFr_013_T3r_1	DQ124415	BT	Holstein	NLD	52.52/4.79	NCBI	MDC-	T3r/T3	T3/T3	(24)
686	NLD_HoFr_014_T3b_1	DQ124416	BT	Holstein	NLD	52.52/4.79	NCBI	MDC-	T3b/T3	T3/T3	(24)
687	NLD_HoFr_015_T3a_1	DQ124417	BT	Holstein	NLD	52.52/4.79	NCBI	MDC-	T3a/T3a	T3/T3	(24)
688	NLD_HoFr_016_T3a_1	DQ124418	BT	Holstein	NLD	52.52/4.79	NCBI	MDC-	T3a/T3a	T4/T3	(24)
689	CHN_Diqi_001_I1x_1	GU256940	BI	Diqin	CHN	30.28/84.73	NCBI	MDC-	I1/I	I/I	(25)
690	KOR_Hanw_002_T3x_1	KF926377	BT	Hanwoo	KOR	35.91/127.77	NCBI	MDC-	T3/T3	T3/T3	(26)
691	DEU_Heck_001_T3r_1	HM045018	BT	Heck cattle	DEU	48.14/11.58	NCBI	MDC-	T3r/T3	T3/T3	(27)

No.	Fasta name	Acc. no.	SP	Breed	ORG	La-Y/Lo-X	Source	MDCT	MTP M/D	Beast M/D	Author
692	HUN_HunG_001_T3r_1	GQ129207	BT	Hungarian Grey	HUN	47.27/20.45	NCBI	MDC-	T3r/T3r	T3/T3	(27)
693	UKR_UkrG_001_T3r_1	GQ129208	BT	Ukrainian Grey	UKR	50.45/30.52	NCBI	MDC-	T3r/T3	T3/T3	(27)
694	CHN_Prim_001_10560	KF525852	BP	-	CHN	30.28/84.73	NCBI	-DC-	RPQ/T3	-/R	(28)
695	ETH_Bora_001_T1b_3	SRS1346805	BI	Boran	ETH	5.39/38.27	rWGS	MDC-	T1b/T1	T1/T1	(29)
696	ETH_Bora_002_T1b_3	SRS1429491	BI	Boran	ETH	5.39/38.27	rWGS	MDC-	T1b/T1	T1/T1	(29)
697	ETH_Bora_003_T1x_3	SRS1727978	BI	Boran	ETH	5.39/38.27	rWGS	MDC-	T1/T1	T1/T1	(29)
698	ETH_Bora_004_T1x_3	SRS1429391	BI	Boran	ETH	5.39/38.27	rWGS	MDC-	T1/T1	T1/T1	(29)
699	ETH_Bora_005_T1b_3	SRS1437860	BI	Boran	ETH	5.39/38.27	rWGS	MDC-	T1b/T1b	T1/T1	(29)
700	ETH_Ogad_001_T1b_3	SRS1512520	BI	Ogaden	ETH	6.55/41.79	rWGS	MDC-	T1b/T1	T1/T1	(29)
701	ETH_Ogad_002_T1b_3	SRS1512522	BI	Ogaden	ETH	6.55/41.79	rWGS	MDC-	T1b/T1b	T1/T1	(29)
702	ETH_Ogad_003_T1x_3	SRS1512525	BI	Ogaden	ETH	6.55/41.79	rWGS	MDC-	T1/T1	T1/T1	(29)
703	ETH_Ogad_004_T1b_3	SRS1512526	BI	Ogaden	ETH	6.55/41.79	rWGS	MDC-	T1b/T1b	T1/T1	(29)
704	SDN_Kena_001_T1x_3	SRS1439369	BI	Kenana	SDN	13.18/32.75	rWGS	MDC-	T1/T1	T1/T1	(29)
705	SDN_Kena_002_T1x_3	SRS1446916	BI	Kenana	SDN	13.18/32.75	rWGS	MDC-	T1/T1	T1/T1	(29)
706	SDN_Kena_003_T1b_3	SRS1437861	BI	Kenana	SDN	13.18/32.75	rWGS	MDC-	T1b/T1b	T1/T1	(29)
707	SDN_Kena_004_T1b_3	SRS1439366	BI	Kenana	SDN	13.18/32.75	rWGS	MDC-	T1b/T1b	T1/T1	(29)
708	SDN_Kena_005_T1b_3	SRS1439367	BI	Kenana	SDN	13.18/32.75	rWGS	MDC-	T1b/T1	T1/T1	(29)
709	SDN_Kena_006_T1x_3	SRS1439368	BI	Kenana	SDN	13.18/32.75	rWGS	MDC-	T1/T1	T1/T1	(29)
710	SDN_Kena_007_T1b_3	SRS1439001	BI	Kenana	SDN	13.18/32.75	rWGS	MDC-	T1b/T1b	T1/T1	(29)
711	SDN_Kena_008_T1c_3	SRS1439005	BI	Kenana	SDN	13.18/32.75	rWGS	MDC-	T1c/T1c	T1/T1	(29)
712	SEN_Ndam_001_T1x_3	SRS1512498	BT	N'Dama	SEN	13.21/-13.11	rWGS	MDC-	T1/T1d	T1/T1	(29)
713	SEN_Ndam_002_T1x_3	SRS1512514	BT	N'Dama	SEN	13.21/-13.11	rWGS	MDC-	T1/T1d	T1/T1	(29)
714	UGA_Anko_001_T1b_3	SRS1338704	BT	Ankole	UGA	0.27/32.29	rWGS	MDC-	T1b/T1	T1/T1	(29)
715	UGA_Anko_002_T1b_3	SRS1496254	BT	Ankole	UGA	0.27/31.18	rWGS	MDC-	T1b/T1	T1/T1	(29)
716	UGA_Anko_003_T1c_3	SRS1496257	BT	Ankole	UGA	0.27/31.18	rWGS	MDC-	T1c/T1c	T1/T1	(29)
717	UGA_Anko_004_T1b_3	SRS1496259	BT	Ankole	UGA	0.27/31.18	rWGS	MDC-	T1b/T1a	T1/T1	(29)
718	UGA_Anko_005_T1b_3	SRS1496260	BT	Ankole	UGA	0.27/31.18	rWGS	MDC-	T1b/T1a	T1/T1	(29)
719	UGA_Anko_006_T1b_3	SRS1496261	BT	Ankole	UGA	0.27/31.18	rWGS	MDC-	T1b/T1	T1/T1	(29)
720	UGA_Anko_007_T1x_3	SRS1496262	BT	Ankole	UGA	0.27/31.18	rWGS	MDC-	T1/T1b	T1/T1	(29)

No.	Fasta name	Acc. no.	SP	Breed	ORG	La-Y/Lo-X	Source	MDCT	MTP M/D	Beast M/D	Author
721	UGA_Anko_008_T1x_3	SRS1496265	BT	Ankole	UGA	0.27/31.18	rWGS	MDC-	T1/T1	T1/T1	(29)
722	DEU_VieG_001_T3x_3	SRS628363	BT	Gelbvieh (German Yellow)	CAN	52.47/-113.75	rWGS	MDC-	T3/T3	T3/T3	(30)
723	DEU_VieG_002_T3x_3	SRS629675	BT	Gelbvieh (German Yellow)	CAN	52.47/-113.75	rWGS	MDC-	T3/T3	T3/T3	(30)
724	DEU_VieG_003_T3r_3	SRS631222	BT	Gelbvieh (German Yellow)	CAN	52.47/-113.75	rWGS	MDC-	T3r/T3j	T3/T3	(30)
725	DEU_VieG_004_T3r_3	SRS629679	BT	Gelbvieh (German Yellow)	CAN	52.47/-113.75	rWGS	MDC-	T3r/T3d	T3/T3	(30)
726	DEU_VieG_005_T3x_3	SRS629674	BT	Gelbvieh (German Yellow)	CAN	52.47/-113.75	rWGS	MDC-	T3/T3r	T3/T3	(30)
727	DEU_VieG_006_T3x_3	SRS629676	BT	Gelbvieh (German Yellow)	CAN	52.47/-113.75	rWGS	MDC-	T3/T3	T3/T3	(30)
728	DEU_VieG_007_T3p_3	SRS631221	BT	Gelbvieh (German Yellow)	CAN	52.47/-113.75	rWGS	MDC-	T3p/T3c	T3/T3	(30)
729	FRA_Limo_002_T3r_3	SRS378721	BT	Limousin	CAN	52.47/-113.75	rWGS	MDC-	T3r/T3	T3/T3	(30)
730	GBR_AngR_001_T3r_3	SRS629341	BT	Red Angus	CAN	52.47/-113.75	rWGS	MDC-	T3r/T1e	T3/T3	(30)
731	GBR_Angu_008_T3x_3	SRS629677	BT	Angus	CAN	52.47/-113.75	rWGS	MDC-	T3/T1e	T3/T3	(30)
732	GBR_Angu_009_T3s_3	SRS631217	BT	Angus	CAN	52.47/-113.75	rWGS	MDC-	T3s/T3	T3/T3	(30)
733	GBR_Angu_010_T3r_3	SRS428688	BT	Angus	CAN	52.47/-113.75	rWGS	MDC-	T3r/T3d	T3/T3	(30)
734	GBR_Angu_011_T3r_3	SRS641738	BT	Angus	CAN	52.47/-113.75	rWGS	MDC-	T3r/T3	T3/T3	(30)
735	GBR_Angu_012_T3r_3	SRS641740	BT	Angus	CAN	52.47/-113.75	rWGS	MDC-	T3r/T3	T3/T3	(30)
736	GBR_Angu_013_T3x_3	SRS629088	BT	Angus	CAN	52.47/-113.75	rWGS	MDC-	T3/T3	T3/T3	(30)
737	GBR_Angu_014_T1x_3	SRS629091	BT	Angus	CAN	52.47/-113.75	rWGS	MDC-	T1/T1	T1/T1	(30)
738	GBR_Angu_018_T3n_3	SRS631214	BT	Angus	CAN	52.47/-113.75	rWGS	MDC-	T3n/T1b	T3/T3	(30)
739	GBR_Angu_019_T3d_3	SRS428686	BT	Angus	CAN	52.47/-113.75	rWGS	MDC-	T3d/T3	T3/T3	(30)
740	GBR_Angu_020_T3r_3	SRS641739	BT	Angus	CAN	52.47/-113.75	rWGS	MDC-	T3r/T3d	T3/T3	(30)
741	GBR_Angu_021_T3r_3	SRS629330	BT	Angus	CAN	52.47/-113.75	rWGS	MDC-	T3r/T3	T3/T3	(30)
742	GBR_Angu_022_T6x_3	SRS629688	BT	Angus	CAN	52.47/-113.75	rWGS	MDC-	T6/T6	T3/T1	(30)
743	GBR_Angu_023_T3n_3	SRS629690	BT	Angus	CAN	52.47/-113.75	rWGS	MDC-	T3n/T3b	T3/T3	(30)
744	GBR_Here_003_T3x_3	SRS631191	BT	Hereford	CAN	52.47/-113.75	rWGS	MDC-	T3/T3	T3/T3	(30)
745	GBR_Here_004_T3x_3	SRS631218	BT	Hereford	CAN	52.47/-113.75	rWGS	MDC-	T3/T3	T3/T3	(30)
746	NLD_HoFr_004_T3x_3	SRS629342	BT	Holstein	CAN	52.47/-113.75	rWGS	MDC-	T3/T3	T3/T3	(30)
747	CHE_Simm_008_T3r_4	T-S	BT	Fleckvieh	AUT	48.15/14.03	rWGS	MDC-	T3r/T3	T3/T3	T-S
748	CHE_Simm_006_T3x_3	T-S	BT	Fleckvieh	AUT	48.15/14.03	rWGS	MDC-	T3/T3	T3/T3	T-S
749	CHE_Simm_007_T3i_3	T-S	BT	Fleckvieh	AUT	48.15/14.03	rWGS	MDC-	T3i/T3	T3/T3	T-S

No.	Fasta name	Acc. no.	SP	Breed	ORG	La-Y/Lo-X	Source	MDCT	MTP M/D	Beast M/D	Author
750	CHE_Simm_001_T3r_4	SRS666732	BT	Simmental	CAN	52.47/-113.75	rWGS	MDC-	T3r/T3	T3/T3	(30)
751	CHE_Simm_002_T3r_4	SRS666781	BT	Simmental	CAN	52.47/-113.75	rWGS	MDC-	T3r/T3	T3/T3	(30)
752	CHE_Simm_003_T3x_4	SRS666789	BT	Simmental	CAN	52.47/-113.75	rWGS	MDC-	T3/T3c	T3/T3	(30)
753	CHE_Simm_004_T3n_4	SRS666784	BT	Simmental	CAN	52.47/-113.75	rWGS	MDC-	T3n/T3b	T3/T3	(30)
754	CHE_Simm_005_T3x_4	SRS666776	BT	Simmental	CAN	52.47/-113.75	rWGS	MDC-	T3/T3	T3/T3	(30)
755	DEU_VieG_008_T3x_4	SRS628368	BT	Gelbvieh (German Yellow)	CAN	52.47/-113.75	rWGS	MDC-	T3/T3i	T3/T3	(30)
756	DEU_VieG_009_T3r_4	SRS629684	BT	Gelbvieh (German Yellow)	CAN	52.47/-113.75	rWGS	MDC-	T3r/T3d	T3/T3	(30)
757	DEU_VieG_010_T3x_4	SRS629693	BT	Gelbvieh (German Yellow)	CAN	52.47/-113.75	rWGS	MDC-	T3/T3	T3/T3	(30)
758	DEU_VieG_011_T3r_4	SRS628364	BT	Gelbvieh (German Yellow)	CAN	52.47/-113.75	rWGS	MDC-	T3r/T3d	T3/T3	(30)
759	DEU_VieG_012_T3r_4	SRS628367	BT	Gelbvieh (German Yellow)	CAN	52.47/-113.75	rWGS	MDC-	T3r/T3j	T3/T3	(30)
760	FRA_BlAq_001_T3s_4	SRS428663	BT	Blonde d'Aquitaine	CAN	52.47/-113.75	rWGS	MDC-	T3s/T3	T3/T3	(30)
761	FRA_Char_001_T3r_4	SRS629324	BT	Charolais	CAN	52.47/-113.75	rWGS	MDC-	T3r/T1e	T3/T3	(30)
762	FRA_Char_002_T3r_4	SRS629327	BT	Charolais	CAN	52.47/-113.75	rWGS	MDC-	T3r/T3	T3/T3	(30)
763	FRA_Char_003_T3x_4	SRS428678	BT	Charolais	CAN	52.47/-113.75	rWGS	MDC-	T3/T3	T3/T3	(30)
764	FRA_Char_004_T3n_4	SRS628370	BT	Charolais	CAN	52.47/-113.75	rWGS	MDC-	T3n/T3n	T3/T3	(30)
765	FRA_Char_005_T3r_4	SRS628376	BT	Charolais	CAN	52.47/-113.75	rWGS	MDC-	T3r/T3j	T3/T3	(30)
766	FRA_Char_006_T3r_4	SRS631207	BT	Charolais	CAN	52.47/-113.75	rWGS	MDC-	T3r/T3	T3/T3	(30)
767	FRA_Char_007_T3r_4	SRS666720	BT	Charolais	CAN	52.47/-113.75	rWGS	MDC-	T3r/T3	T3/T3	(30)
768	FRA_Char_008_T3r_4	SRS666710	BT	Charolais	CAN	52.47/-113.75	rWGS	MDC-	T3r/T3	T3/T3	(30)
769	FRA_Char_009_T3r_4	SRS628372	BT	Charolais	CAN	52.47/-113.75	rWGS	MDC-	T3r/T1e	T3/T3	(30)
770	FRA_Limo_001_T3r_4	SRS631220	BT	Limousin	CAN	52.47/-113.75	rWGS	MDC-	T3r/T3	T3/T3	(30)
771	FRA_Limo_003_T3r_4	SRS631219	BT	Limousin	CAN	52.47/-113.75	rWGS	MDC-	T3r/T3	T3/T3	(30)
772	FRA_Limo_004_T3r_4	SRS428694	BT	Limousin	CAN	52.47/-113.75	rWGS	MDC-	T3r/T3	T3/T3	(30)
773	FRA_MAnj_001_T3p_4	SRS631210	BT	Maine Anjou	CAN	52.47/-113.75	rWGS	MDC-	T3p/T3p	T3/T3	(30)
774	FRA_MAnj_002_T3r_4	SRS629692	BT	Maine Anjou	CAN	52.47/-113.75	rWGS	MDC-	T3r/T3q	T3/T3	(30)
775	FRA_MAnj_003_T3r_4	SRS631215	BT	Maine Anjou	CAN	52.47/-113.75	rWGS	MDC-	T3r/T3	T3/T3	(30)
776	FRA_MAnj_004_T3p_4	SRS629671	BT	Maine Anjou	CAN	52.47/-113.75	rWGS	MDC-	T3p/T3p	T3/T3	(30)
777	GBR_AngR_002_T3x_4	SRS641741	BT	Red Angus	CAN	52.47/-113.75	rWGS	MDC-	T3/T3	T3/T3	(30)
778	GBR_AngR_003_T3r_4	SRS666722	BT	Red Angus	CAN	52.47/-113.75	rWGS	MDC-	T3r/T3	T3/T3	(30)

No.	Fasta name	Acc. no.	SP	Breed	ORG	La-Y/Lo-X	Source	MDCT	MTP M/D	Beast M/D	Author
779	GBR_AngR_004_T3r_4	SRS666690	BT	Red Angus	CAN	52.47/-113.75	rWGS	MDC-	T3r/T3j	T3/T3	(30)
780	GBR_Angu_001_T3r_4	SRS428689	BT	Angus	CAN	52.47/-113.75	rWGS	MDC-	T3r/T3	T3/T3	(30)
781	GBR_Angu_002_T3r_4	SRS428684	BT	Angus	CAN	52.47/-113.75	rWGS	MDC-	T3r/T3	T3/T3	(30)
782	GBR_Angu_003_T3r_4	SRS428690	BT	Angus	CAN	52.47/-113.75	rWGS	MDC-	T3r/T3	T3/T3	(30)
783	GBR_Angu_004_T3r_4	SRS629322	BT	Angus	CAN	52.47/-113.75	rWGS	MDC-	T3r/T3	T3/T3	(30)
784	GBR_Angu_005_T3r_4	SRS641715	BT	Angus	CAN	52.47/-113.75	rWGS	MDC-	T3r/T3r	T3/T3	(30)
785	GBR_Angu_006_T3n_4	SRS428675	BT	Angus	CAN	52.47/-113.75	rWGS	MDC-	T3n/T3b	T3/T3	(30)
786	GBR_Angu_007_T3r_4	SRS641713	BT	Angus	CAN	52.47/-113.75	rWGS	MDC-	T3r/T3	T3/T3	(30)
787	GBR_Angu_015_T3r_4	SRS428687	BT	Angus	CAN	52.47/-113.75	rWGS	MDC-	T3r/T3	T3/T3	(30)
788	GBR_Angu_016_T3r_4	SRS428672	BT	Angus	CAN	52.47/-113.75	rWGS	MDC-	T3r/T3	T3/T3	(30)
789	GBR_Angu_017_T3c_4	SRS628375	BT	Angus	CAN	52.47/-113.75	rWGS	MDC-	T3c/T3	T3/T3	(30)
790	GBR_Angu_024_T3r_4	SRS428691	BT	Angus	CAN	52.47/-113.75	rWGS	MDC-	T3r/T1e	T3/T3	(30)
791	GBR_Angu_025_T3q_4	SRS666693	BT	Angus	CAN	52.47/-113.75	rWGS	MDC-	T3q/T3d	T3/T3	(30)
792	GBR_Angu_026_T3r_4	SRS666773	BT	Angus	CAN	52.47/-113.75	rWGS	MDC-	T3r/T3	T3/T3	(30)
793	GBR_Angu_027_T3r_4	SRS666774	BT	Angus	CAN	52.47/-113.75	rWGS	MDC-	T3r/T3	T3/T3	(30)
794	GBR_Angu_028_T3r_4	SRS666771	BT	Angus	CAN	52.47/-113.75	rWGS	MDC-	T3r/T3	T3/T3	(30)
795	GBR_Angu_029_T3r_4	SRS666769	BT	Angus	CAN	52.47/-113.75	rWGS	MDC-	T3r/T3	T3/T3	(30)
796	GBR_Here_001_T3x_4	SRS631195	BT	Hereford	CAN	52.47/-113.75	rWGS	MDC-	T3/T3	T3/T3	(30)
797	GBR_Here_002_T3n_4	SRS629694	BT	Hereford	CAN	52.47/-113.75	rWGS	MDC-	T3n/T1b	T3/T3	(30)
798	ITA_Pied_001_T3r_4	SRS428665	BT	Piedmontese	CAN	52.47/-113.75	rWGS	MDC-	T3r/T3	T3/T3	(30)
799	ITA_Pied_002_T3x_4	SRS666704	BT	Piedmontese	CAN	52.47/-113.75	rWGS	MDC-	T3/T3	T3/T3	(30)
800	ITA_Pied_003_T1c_4	SRS666712	BT	Piedmontese	CAN	52.47/-113.75	rWGS	MDC-	T1c/T1c	T1/T1	(30)
801	ITA_Pied_004_T3x_4	SRS666707	BT	Piedmontese	CAN	52.47/-113.75	rWGS	MDC-	T3/T3	T3/T3	(30)
802	ITA_Pied_005_T3r_4	SRS666711	BT	Piedmontese	CAN	52.47/-113.75	rWGS	MDC-	T3r/QT	T3/Q	(30)
803	ITA_Pied_006_T3x_4	SRS666714	BT	Piedmontese	CAN	52.47/-113.75	rWGS	MDC-	T3/T3	T3/T3	(30)
804	NLD_HoFr_001_T3r_4	SRS641735	BT	Holstein	CAN	52.47/-113.75	rWGS	MDC-	T3r/T3	T3/T3	(30)
805	NLD_HoFr_002_T3x_4	SRS629104	BT	Holstein	CAN	52.47/-113.75	rWGS	MDC-	T3/T3	T3/T3	(30)
806	NLD_HoFr_003_T3x_4	SRS641722	BT	Holstein	CAN	52.47/-113.75	rWGS	MDC-	T3/T1e	T3/T3	(30)
807	NLD_HoFr_005_T3r_4	SRS641729	BT	Holstein	CAN	52.47/-113.75	rWGS	MDC-	T3r/T3	T3/T3	(30)

No.	Fasta name	Acc. no.	SP	Breed	ORG	La-Y/Lo-X	Source	MDCT	MTP M/D	Beast M/D	Author
808	NLD_HoFr_006_T3r_4	SRS631189	BT	Holstein	CAN	52.47/-113.75	rWGS	MDC-	T3r/T3	T3/T3	(30)
809	NLD_HoFr_007_T3r_4	SRS631202	BT	Holstein	CAN	52.47/-113.75	rWGS	MDC-	T3r/T3	T3/T3	(30)
810	NLD_HoFr_008_T3r_4	SRS641744	BT	Holstein	CAN	52.47/-113.75	rWGS	MDC-	T3r/T3	T3/T3	(30)
811	T_88_ESP_3780	AY847188	BT	-	ESP	42.85/-2.67	NCBI	-D--	-/T3	-/T3	(31)
812	T_89_ESP_3780	AY847189	BT	-	ESP	42.85/-2.67	NCBI	-D--	-/T3	-/T3	(31)
813	T_90_ESP_3780	AY847190	BT	-	ESP	42.85/-2.67	NCBI	-D--	-/T3	-/T3	(31)
814	T_91_ESP_3635	AY847191	BT	-	ESP	42.85/-2.67	NCBI	-D--	-/T3	-/T3	(31)
815	T_92_ESP_3780	AY847192	BT	-	ESP	42.85/-2.67	NCBI	-D--	-/T3	-/T3	(31)
816	T_94_ESP_3780	AY847194	BT	-	ESP	42.85/-2.67	NCBI	-D--	-/Q	-/T1	(31)
817	T_95_ESP_3780	AY847195	BT	-	ESP	42.85/-2.67	NCBI	-D--	-/T3	-/T3	(31)
818	T_96_ESP_3780	AY847196	BT	-	ESP	42.85/-2.67	NCBI	-D--	-/T3	-/T3	(31)
819	T_97_ESP_3740	AY847197	BT	-	ESP	42.85/-2.67	NCBI	-D--	-/P	-/P	(31)
820	T_98_ESP_3800	AY847198	BT	-	ESP	42.85/-2.67	NCBI	-D--	-/T1	-/T1	(31)
821	T_99_ESP_3780	AY847199	BT	-	ESP	42.85/-2.67	NCBI	-D--	-/T3	-/T3	(31)
822	T_00_ESP_3780	AY847200	BT	-	ESP	42.85/-2.67	NCBI	-D--	-/T3	-/T3	(31)
823	T_08_ESP_880	AY847208	BT	-	ESP	40.35/-1.11	NCBI	-D--	-/T3n	-/T3	(31)
824	T_01_IRN_3652	JQ280501	BT	-	IRN	32.08/48.40	NCBI	-D--	-/T1	-/T1	(32)
825	T_02_IRN_1800	JQ280502	BT	-	IRN	34.80/48.52	NCBI	-D--	-/T3	-/T3	(32)
826	T_03_IRN_8021	JQ280503	BT	-	IRN	29.91/29.91	NCBI	-D--	-/T1f	-/T1	(32)
827	T_04_IRN_5687	JQ280504	BT	-	IRN	30.67/51.27	NCBI	-D--	-/Q	-/Q	(32)
828	T_05_IRN_5442	JQ280505	BT	-	IRN	33.79/47.91	NCBI	-D--	-/T2	-/T2	(32)
829	T_06_IRN_8451	JQ280506	BT	-	IRN	35.70/51.39	NCBI	-D--	-/Q	-/T2	(32)
830	T_07_IRN_7364	JQ280507	BT	-	IRN	37.22/50.23	NCBI	-D--	-/T2	-/T2	(32)
831	T_08_IRN_7673	JQ280508	BT	-	IRN	37.22/50.23	NCBI	-D--	-/T3	-/T3	(32)
832	T_09_IRN_7673	JQ280509	BT	-	IRN	37.22/50.23	NCBI	-D--	-/Q	-/T1	(32)
833	T_10_IRN_7673	JQ280510	BT	-	IRN	37.22/50.23	NCBI	-D--	-/Q	-/Q	(32)
834	T_11_IRN_7673	JQ280511	BT	-	IRN	37.22/50.23	NCBI	-D--	-/T2	-/T2	(32)
835	T_12_IRN_7992	JQ280512	BT	-	IRN	37.22/50.23	NCBI	-D--	-/Q	-/Q	(32)
836	T_13_IRN_6523	JQ280513	BT	-	IRN	35.72/50.23	NCBI	-D--	-/T3	-/T3	(32)

No.	Fasta name	Acc. no.	SP	Breed	ORG	La-Y/Lo-X	Source	MDCT	MTP M/D	Beast M/D	Author
837	T_14_IRN_7673	JQ280514	BT	-	IRN	37.22/50.23	NCBI	-D--	-/T2	-/T2	(32)
838	T_15_IRN_6847	JQ280515	BT	-	IRN	33.50/48.70	NCBI	-D--	-/Q	-/T1	(32)
839	T_04_GBR_220	GU169304	BT	-	GBR	52.08/-2.65	NCBI	-D--	-/T1e	-/T3	(33)
840	T_09_GBR_220	GU169309	BT	-	GBR	52.08/-2.65	NCBI	-D--	-/T3b	-/T3	(33)
841	P_74_GBR_6174	DQ915574	BP	-	GBR	51.28/-2.74	NCBI	-D--	-/P	-/P	(34)
842	T_59_FRA_4950	DQ915559	BT	-	FRA	48.45/3.35	NCBI	-D--	-/T3	-/T3	(34)
843	P_58_GBR_3370	DQ915558	BP	-	GBR	53.72/-0.51	NCBI	-D--	-/P	-/P	(34)
844	P_54_SVN_8020	DQ915554	BP	-	SVN	45.68/13.97	NCBI	-D--	-/P	-/P	(34)
845	P_53_IRN_7000	DQ915553	BP	-	IRN	35.98/50.58	NCBI	-D--	-/Q	-/Q	(34)
846	P_38_GBR_12290	DQ915538	BP	-	GBR	54.28/-0.40	NCBI	-D--	-/P	-/P	(34)
847	P_37_GBR_5936	DQ915537	BP	-	GBR	53.08/-1.64	NCBI	-D--	-/P1	-/P	(34)
848	P_32_GBR_4662	DQ915532	BP	-	GBR	53.08/-1.64	NCBI	-D--	-/T3	-/T3	(34)
849	P_27_GBR_4831	DQ915527	BP	-	GBR	53.08/-1.64	NCBI	-D--	-/T3	-/T3	(34)
850	P_24_GBR_3905	DQ915524	BP	-	GBR	51.29/-2.73	NCBI	-D--	-/P	-/P	(34)
851	P_22_DEU_12030	DQ915522	BP	-	DEU	51.04/8.67	NCBI	-D--	-/P	-/P	(34)
852	DEU_Prim_001_5800	DQ915540	BP	-	DEU	52.15/11.22	NCBI	-D--	-/T3j	-/R	(34)
853	P_25_DEN_9830	JQ269325	BP	-	DNK	55.64/9.55	NCBI	-D--	-/P	-/P	(35)
854	P_26_DEN_3600	JQ269326	BP	-	DNK	56.15/10.11	NCBI	-D--	-/P	-/P	(35)
855	P_27_DEN_8460	JQ269327	BP	-	DNK	56.69/10.10	NCBI	-D--	-/P	-/P	(35)
856	P_28_DEN_2865	JQ269328	BP	-	DNK	55.31/10.79	NCBI	-D--	-/P	-/P	(35)
857	P_29_DEN_2945	JQ269329	BP	-	DNK	55.71/9.92	NCBI	-D--	-/P	-/P	(35)
858	P_30_DEN_9410	JQ269330	BP	-	DNK	54.92/8.99	NCBI	-D--	-/P1	-/P	(35)
859	P_31_DEN_9810	JQ269331	BP	-	DNK	54.92/8.99	NCBI	-D--	-/P	-/P	(35)
860	P_32_DEN_5855	JQ269332	BP	-	DNK	55.34/10.31	NCBI	-D--	-/P	-/P	(35)
861	P_33_DEN_5425	JQ269333	BP	-	DNK	54.93/9.25	NCBI	-D--	-/P	-/P	(35)
862	P_34_DEN_4060	JQ269334	BP	-	DNK	54.77/11.77	NCBI	-D--	-/P	-/P	(35)
863	T_1_KOR_0	AY337527	BT	Korean Youngju Yellow	KOR	37.43/128.09	DDBJ	-D--	-/P2	-/P	(36)
864	T_02_DEU_6110	AY594302	BT	-	DEU	48.49/11.93	NCBI	-D--	-/T3r	-/T3	(37)
865	T_31_FIN_985	KF233431	BT	-	FIN	61.00/24.47	NCBI	-D--	-/T3	-/T3	(38)

No.	Fasta name	Acc. no.	SP	Breed	ORG	La-Y/Lo-X	Source	MDCT	MTP M/D	Beast M/D	Author
866	T_32_FIN_324	KF233432	BT	-	FIN	59.92/20.91	NCBI	-D--	-/T3n	-/T3	(38)
867	T_33_FIN_384	KF233433	BT	-	FIN	59.92/20.91	NCBI	-D--	-/T3	-/T3	(38)
868	T_40_FIN_350	KF233440	BT	-	FIN	61.80/29.96	NCBI	-D--	-/T3	-/T3	(38)
869	T_45_FIN_606	KF233445	BT	-	FIN	60.45/22.28	NCBI	-D--	-/T2	-/T2	(38)
870	T_46_FIN_740	KF233446	BT	-	FIN	60.45/22.28	NCBI	-D--	-/T3n	-/T3	(38)
871	T_49_FIN_984	KF233449	BT	-	FIN	60.47/22.18	NCBI	-D--	-/T3	-/T3	(38)
872	T_50_FIN_567	KF233450	BT	-	FIN	61.65/27.28	NCBI	-D--	-/T3j	-/T3	(38)
873	T_51_FIN_431	KF233451	BT	-	FIN	60.47/22.03	NCBI	-D--	-/T3	-/T3	(38)
874	T_56_FIN_637	KF233456	BT	-	FIN	61.50/25.68	NCBI	-D--	-/T3	-/T3	(38)
875	T_59_FIN_238	KF233459	BT	-	FIN	61.80/29.96	NCBI	-D--	-/T1e	-/T3	(38)
876	T_1_JPN_0	LC314257	BT	Japanese Shorthorn	JPN	39.78/140.98	DDBJ	-D--	-/P2	-/P	(39)
877	T_2_JPN_0	LC314260	BT	Japanese Shorthorn	JPN	39.78/140.98	DDBJ	-D--	-/P2	-/P	(39)
878	T_3_JPN_0	LC314262	BT	Japanese Shorthorn	JPN	39.78/140.98	DDBJ	-D--	-/P2	-/P	(39)
879	T_4_JPN_0	LC314263	BT	Japanese Shorthorn	JPN	39.78/140.98	DDBJ	-D--	-/P2	-/P	(39)
880	T_5_JPN_0	LC314264	BT	Japanese Shorthorn	JPN	39.78/140.98	DDBJ	-D--	-/P2	-/P	(39)
881	T_6_JPN_0	LC314265	BT	Japanese Shorthorn	JPN	39.78/140.98	DDBJ	-D--	-/P2	-/P	(39)
882	T_7_JPN_0	LC314266	BT	Japanese Shorthorn	JPN	39.78/140.98	DDBJ	-D--	-/P2	-/P	(39)
883	T_8_JPN_0	LC314267	BT	Japanese Shorthorn	JPN	39.78/140.98	DDBJ	-D--	-/P2	-/P	(39)
884	T_9_JPN_0	LC314270	BT	Japanese Shorthorn	JPN	39.78/140.98	DDBJ	-D--	-/P2	-/P	(39)
885	P_15_HUN_6000	EF362615	BP	-	HUN	47.87/21.12	NCBI	-D--	-/P	-/P	(40)
886	P_80_FRA_3204	EF187280	BP	-	FRA	47.75/0.19	NCBI	-D--	-/P	-/P	(41)
887	T_18_JER_12350	DQ020118	BT	-	ISR	31.79/35.17	NCBI	-D--	-/T2	-/T2	(42)
888	T_09_TUR_7150	KF307209	BT	-	TUR	36.76/37.31	NCBI	-D--	-/Q	-/T1	(43)
889	T_10_TUR_7150	KF307210	BT	-	TUR	36.76/37.31	NCBI	-D--	-/Q	-/Q	(43)
890	T_11_TUR_7150	KF307211	BT	-	TUR	36.76/37.31	NCBI	-D--	-/T3p	-/T3	(43)
891	T_12_FRA_5900	KF307212	BT	-	FRA	48.83/2.39	NCBI	-D--	-/Q	-/Q	(43)
892	T_13_FRA_5900	KF307213	BT	-	FRA	48.83/2.39	NCBI	-D--	-/T3	-/T3	(43)
893	T_14_FRA_5900	KF307214	BT	-	FRA	48.83/2.39	NCBI	-D--	-/T3	-/T3	(43)
894	T_15_FRA_5900	KF307215	BT	-	FRA	48.83/2.39	NCBI	-D--	-/T3r	-/T3	(43)

No.	Fasta name	Acc. no.	SP	Breed	ORG	La-Y/Lo-X	Source	MDCT	MTP M/D	Beast M/D	Author
895	T_16_FRA_5900	KF307216	BT	-	FRA	48.83/2.39	NCBI	-D--	-/T3	-/T3	(43)
896	T_17_FRA_5900	KF307217	BT	-	FRA	48.83/2.39	NCBI	-D--	-/T3	-/T3	(43)
897	T_18_FRA_7150	KF307218	BT	-	FRA	44.34/4.46	NCBI	-D--	-/T3	-/T3	(43)
898	T_19_FRA_7150	KF307219	BT	-	FRA	44.34/4.46	NCBI	-D--	-/T3	-/T3	(43)
899	T_20_ROM_6887	KF307220	BT	-	ROU	45.11/26.97	NCBI	-D--	-/T3	-/T3	(43)
900	T_21_ROM_7000	KF307221	BT	-	ROU	45.11/26.97	NCBI	-D--	-/T3	-/T3	(43)
901	T_22_ROM_7000	KF307222	BT	-	ROU	45.11/26.97	NCBI	-D--	-/Q	-/Q	(43)
902	T_23_ROM_6917	KF307223	BT	-	ROU	45.46/25.94	NCBI	-D--	-/T3p	-/T3	(43)
903	T_24_FRA_7100	KF307224	BT	-	FRA	44.17/5.13	NCBI	-D--	-/T3	-/T3	(43)
904	T_25_BUG_7750	KF307225	BT	-	BGR	42.66/24.06	NCBI	-D--	-/T3	-/T3	(43)
905	T_26_BUG_7750	KF307226	BT	-	BGR	42.66/24.06	NCBI	-D--	-/Q	-/Q	(43)
906	T_27_BUG_7750	KF307227	BT	-	BGR	42.66/24.06	NCBI	-D--	-/Q	-/Q	(43)
907	T_28_BUG_7750	KF307228	BT	-	BGR	42.66/24.06	NCBI	-D--	-/Q	-/Q	(43)
908	T_29_BUG_7386	KF307229	BT	-	BGR	42.24/26.44	NCBI	-D--	-/Q	-/Q	(43)
909	T_30_BUG_7423	KF307230	BT	-	BGR	42.24/26.44	NCBI	-D--	-/Q	-/Q	(43)
910	T_31_BUG_7433	KF307231	BT	-	BGR	42.24/26.44	NCBI	-D--	-/T3	-/T3	(43)
911	T_32_BUG_7413	KF307232	BT	-	BGR	42.24/26.44	NCBI	-D--	-/T3	-/T3	(43)
912	T_33_BUG_7350	KF307233	BT	-	BGR	42.24/26.44	NCBI	-D--	-/Q	-/T1	(43)
913	T_34_BUG_7433	KF307234	BT	-	BGR	42.24/26.44	NCBI	-D--	-/Q	-/T1	(43)
914	T_36_BUG_7350	KF307236	BT	-	BGR	42.24/26.44	NCBI	-D--	-/T3	-/T3	(43)
915	T_37_BUG_7397	KF307237	BT	-	BGR	42.24/26.44	NCBI	-D--	-/T3	-/T3	(43)
916	T_38_BUG_6600	KF307238	BT	-	BGR	42.24/26.44	NCBI	-D--	-/Q	-/T1	(43)
917	T_39_BUG_6599	KF307239	BT	-	BGR	42.24/26.44	NCBI	-D--	-/T3	-/T3	(43)
918	T_40_BUG_6600	KF307240	BT	-	BGR	42.24/26.44	NCBI	-D--	-/T2d	-/T2	(43)
919	T_41_BUG_6600	KF307241	BT	-	BGR	42.24/26.44	NCBI	-D--	-/T3	-/T3	(43)
920	T_42_TUR_4450	KF307242	BT	-	TUR	41.73/27.48	NCBI	-D--	-/T3	-/T3	(43)
921	T_43_TUR_4450	KF307243	BT	-	TUR	41.73/27.48	NCBI	-D--	-/Q	-/Q	(43)
922	T_44_TUR_4568	KF307244	BT	-	TUR	41.73/27.48	NCBI	-D--	-/T1f	-/T1	(43)
923	T_45_TUR_4450	KF307245	BT	-	TUR	41.73/27.48	NCBI	-D--	-/T3	-/T3	(43)

No.	Fasta name	Acc. no.	SP	Breed	ORG	La-Y/Lo-X	Source	MDCT	MTP M/D	Beast M/D	Author
924	T_46_TUR_4450	KF307246	BT	-	TUR	41.73/27.48	NCBI	-D--	-/Q	-/Q	(43)
925	T_47_TUR_4450	KF307247	BT	-	TUR	41.73/27.48	NCBI	-D--	-/T3	-/T3	(43)
926	T_48_TUR_4450	KF307248	BT	-	TUR	41.73/27.48	NCBI	-D--	-/Q	-/T1	(43)
927	T_49_BUG_8000	KF307249	BT	-	BGR	43.41/25.88	NCBI	-D--	-/T3	-/T3	(43)
928	T_50_BUG_8000	KF307250	BT	-	BGR	43.41/25.88	NCBI	-D--	-/T3	-/T3	(43)
929	T_51_BUG_8000	KF307251	BT	-	BGR	43.41/25.88	NCBI	-D--	-/T3	-/T3	(43)
930	T_52_BUG_8000	KF307252	BT	-	BGR	43.41/25.88	NCBI	-D--	-/T3	-/T3	(43)
931	T_53_BUG_8000	KF307253	BT	-	BGR	43.41/25.88	NCBI	-D--	-/T3	-/T3	(43)
932	T_54_BUG_8000	KF307254	BT	-	BGR	43.41/25.88	NCBI	-D--	-/Q	-/T1	(43)
933	T_55_BUG_8000	KF307255	BT	-	BGR	43.41/25.88	NCBI	-D--	-/T3	-/T3	(43)
934	T_56_BUG_8000	KF307256	BT	-	BGR	43.41/25.88	NCBI	-D--	-/T3	-/T3	(43)
935	T_57_BUG_8000	KF307257	BT	-	BGR	43.41/25.88	NCBI	-D--	-/T3	-/T3	(43)
936	T_58_BUG_8000	KF307258	BT	-	BGR	42.24/26.07	NCBI	-D--	-/Q	-/Q	(43)
937	T_59_BUG_8094	KF307259	BT	-	BGR	42.24/26.07	NCBI	-D--	-/Q	-/T1	(43)
938	T_60_BUG_8000	KF307260	BT	-	BGR	42.24/26.07	NCBI	-D--	-/Q	-/Q	(43)
939	T_61_BUG_8000	KF307261	BT	-	BGR	42.24/26.07	NCBI	-D--	-/Q	-/Q	(43)
940	T_62_BUG_7900	KF307262	BT	-	BGR	42.24/26.07	NCBI	-D--	-/T3	-/T3	(43)
941	T_63_BUG_7900	KF307263	BT	-	BGR	42.24/26.07	NCBI	-D--	-/Q	-/Q	(43)
942	T_64_BUG_7900	KF307264	BT	-	BGR	42.24/26.07	NCBI	-D--	-/T3	-/T3	(43)
943	T_65_BUG_7900	KF307265	BT	-	BGR	42.24/26.07	NCBI	-D--	-/T3	-/T3	(43)
944	T_66_BUG_7900	KF307266	BT	-	BGR	42.24/26.07	NCBI	-D--	-/Q	-/Q	(43)
945	T_67_BUG_7900	KF307267	BT	-	BGR	42.24/26.07	NCBI	-D--	-/Q	-/T1	(43)
946	T_68_BUG_7900	KF307268	BT	-	BGR	42.24/26.07	NCBI	-D--	-/T3n	-/T3	(43)
947	T_69_BUG_7900	KF307269	BT	-	BGR	42.24/26.07	NCBI	-D--	-/Q	-/T1	(43)
948	T_70_BUG_7900	KF307270	BT	-	BGR	42.24/26.07	NCBI	-D--	-/Q	-/Q	(43)
949	T_71_BUG_7900	KF307271	BT	-	BGR	42.24/26.07	NCBI	-D--	-/T3	-/T3	(43)
950	T_73_BIH_7050	KF307273	BT	-	BIH	43.99/17.48	NCBI	-D--	-/Q	-/Q	(43)
951	T_74_BIH_7050	KF307274	BT	-	BIH	43.99/17.48	NCBI	-D--	-/T3	-/T3	(43)
952	T_75_BIH_7050	KF307275	BT	-	BIH	43.99/17.48	NCBI	-D--	-/T3	-/T3	(43)

No.	Fasta name	Acc. no.	SP	Breed	ORG	La-Y/Lo-X	Source	MDCT	MTP M/D	Beast M/D	Author
953	T_76_BUG_7600	KF307276	BT	-	BGR	43.70/27.83	NCBI	-D--	-/Q	-/T1	(43)
954	T_77_BUG_7600	KF307277	BT	-	BGR	43.70/27.83	NCBI	-D--	-/Q	-/Q	(43)
955	T_78_BUG_7600	KF307278	BT	-	BGR	43.70/27.83	NCBI	-D--	-/Q	-/Q	(43)
956	T_79_ROM_6350	KF307279	BT	-	ROU	44.06/26.12	NCBI	-D--	-/Q	-/Q	(43)
957	T_80_ROM_7100	KF307280	BT	-	ROU	46.54/23.71	NCBI	-D--	-/T3	-/T3	(43)
958	T_81_BUG_7579	KF307281	BT	-	BGR	43.14/25.61	NCBI	-D--	-/Q	-/Q	(43)
959	T_82_BUG_7400	KF307282	BT	-	BGR	43.14/25.61	NCBI	-D--	-/T3	-/T3	(43)
960	T_83_BUG_7400	KF307283	BT	-	BGR	43.14/25.61	NCBI	-D--	-/T3	-/T3	(43)
961	T_84_BUG_7500	KF307284	BT	-	BGR	43.14/25.61	NCBI	-D--	-/Q	-/Q	(43)
962	T_85_BUG_7500	KF307285	BT	-	BGR	43.14/25.61	NCBI	-D--	-/T3	-/T3	(43)
963	T_86_BUG_7851	KF307286	BT	-	BGR	43.14/25.61	NCBI	-D--	-/T2	-/T2	(43)
964	T_87_BUG_7437	KF307287	BT	-	BGR	43.14/25.61	NCBI	-D--	-/T2	-/T2	(43)
965	T_88_BUG_7400	KF307288	BT	-	BGR	43.14/25.61	NCBI	-D--	-/T2	-/T2	(43)
966	T_89_BUG_7400	KF307289	BT	-	BGR	43.14/25.61	NCBI	-D--	-/T2	-/T2	(43)
967	T_90_FRA_6875	KF307290	BT	-	FRA	48.87/2.36	NCBI	-D--	-/T3	-/T3	(43)
968	T_91_FRA_7146	KF307291	BT	-	FRA	48.87/2.36	NCBI	-D--	-/T3	-/T3	(43)
969	T_92_FRA_7200	KF307292	BT	-	FRA	48.87/2.36	NCBI	-D--	-/T3	-/T3	(43)
970	T_93_FRA_7272	KF307293	BT	-	FRA	48.87/2.36	NCBI	-D--	-/T3	-/T3	(43)
971	T_94_FRA_6802	KF307294	BT	-	FRA	48.87/2.36	NCBI	-D--	-/T3	-/T3	(43)
972	T_95_SYR_5000	KF307295	BT	-	SYR	36.67/41.06	NCBI	-D--	-/T3	-/T3	(43)
973	T_96_ITA_7750	KF307296	BT	-	ITA	40.68/16.64	NCBI	-D--	-/T3	-/T3	(43)
974	T_97_ITA_7750	KF307297	BT	-	ITA	40.68/16.64	NCBI	-D--	-/T3	-/T3	(43)
975	T_98_ITA_7750	KF307298	BT	-	ITA	40.68/16.64	NCBI	-D--	-/T3	-/T3	(43)
976	T_00_ITA_7750	KF307300	BT	-	ITA	40.68/16.64	NCBI	-D--	-/T3	-/T3	(43)
977	T_01_ROM_6750	KF307301	BT	-	ROU	45.66/20.90	NCBI	-D--	-/T3	-/T3	(43)
978	T_02_ROM_6750	KF307302	BT	-	ROU	45.66/20.90	NCBI	-D--	-/T3	-/T3	(43)
979	T_03_ROM_6750	KF307303	BT	-	ROU	45.66/20.90	NCBI	-D--	-/T2	-/T2	(43)
980	T_04_ROM_6750	KF307304	BT	-	ROU	45.66/20.90	NCBI	-D--	-/T3	-/T3	(43)
981	T_05_ROM_6750	KF307305	BT	-	ROU	45.66/20.90	NCBI	-D--	-/T2	-/T2	(43)

No.	Fasta name	Acc. no.	SP	Breed	ORG	La-Y/Lo-X	Source	MDCT	MTP M/D	Beast M/D	Author
982	T_06_ROM_6750	KF307306	BT	-	ROU	45.66/20.90	NCBI	-D--	-/T3	-/T3	(43)
983	T_07_ROM_6925	KF307307	BT	-	ROU	45.66/20.90	NCBI	-D--	-/Q	-/Q	(43)
984	T_08_ROM_6925	KF307308	BT	-	ROU	45.66/20.90	NCBI	-D--	-/Q	-/Q	(43)
985	T_09_ROM_6925	KF307309	BT	-	ROU	45.66/20.90	NCBI	-D--	-/T3	-/T3	(43)
986	T_10_TUR_7800	KF307310	BT	-	TUR	38.47/27.35	NCBI	-D--	-/T3	-/T3	(43)
987	T_11_TUR_7950	KF307311	BT	-	TUR	38.47/27.35	NCBI	-D--	-/Q	-/Q	(43)
988	T_12_TUR_8250	KF307312	BT	-	TUR	38.47/27.35	NCBI	-D--	-/T3	-/T3	(43)
989	T_14_TUR_8250	KF307314	BT	-	TUR	38.47/27.35	NCBI	-D--	-/T3	-/T3	(43)
990	T_15_TUR_8250	KF307315	BT	-	TUR	38.47/27.35	NCBI	-D--	-/T3	-/T3	(43)
991	T_17_TUR_8250	KF307317	BT	-	TUR	38.47/27.35	NCBI	-D--	-/T3	-/T3	(43)
992	T_18_DEU_7200	KF307318	BT	-	DEU	48.89/9.20	NCBI	-D--	-/T3	-/T3	(43)
993	T_19_DEU_7200	KF307319	BT	-	DEU	48.89/9.20	NCBI	-D--	-/T3	-/T3	(43)
994	T_20_DEU_7200	KF307320	BT	-	DEU	48.89/9.20	NCBI	-D--	-/T3	-/T3	(43)
995	T_21_DEU_7200	KF307321	BT	-	DEU	48.89/9.20	NCBI	-D--	-/T3	-/T3	(43)
996	T_22_FRA_6000	KF307322	BT	-	FRA	47.39/-2.94	NCBI	-D--	-/T3	-/T3	(43)
997	T_35_DEU_6000	JX861235	BT	-	DEU	52.04/8.48	NCBI	-D--	-/T3j	-/T3	(43)
998	B_93_SWI_5225	KJ101593	BS	-	CHE	47.09/7.16	NCBI	-D--	-/P	-/P	(44)
999	CHN_Qin_001_0_1	AB177775	BT	Qinghai Yellow	CHN	34.98/97.02	NCBI	-D--	-/R2	-/RPQ	(45)
1000	GBR_Prim/94_5936	FJ392894	BP	-	GBR	-	NCBI	--C-	-/-	-/-	(46)
1001	GBR_Prim/95_7000	FJ392895	BP	-	GBR	-	NCBI	--C-	-/-	-/-	(46)
1002	GBR_Prim/98_7000	FJ392898	BP	-	GBR	-	NCBI	--C-	-/-	-/-	(46)
1003	GBR_Prim/99_7000	FJ392899	BP	-	GBR	-	NCBI	--C-	-/-	-/-	(46)
1004	GBR_Prim/00_7000	FJ392900	BP	-	GBR	-	NCBI	--C-	-/-	-/-	(46)
1005	GBR_Prim/02_7000	FJ392902	BP	-	GBR	-	NCBI	--C-	-/-	-/-	(46)
1006	GBR_Prim/03_7000	FJ392903	BP	-	GBR	-	NCBI	--C-	-/-	-/-	(46)
1007	GBR_Prim/04_7000	FJ392904	BP	-	GBR	-	NCBI	--C-	-/-	-/-	(46)
1008	GBR_Prim/05_7000	FJ392905	BP	-	GBR	-	NCBI	--C-	-/-	-/-	(46)
1009	DEU_Prim/09_5830	FJ392909	BP	-	DEU	-	NCBI	--C-	-/-	-/-	(46)
1010	DEU_Prim/10_7000	FJ392910	BP	-	DEU	-	NCBI	--C-	-/-	-/-	(46)

No.	Fasta name	Acc. no.	SP	Breed	ORG	La-Y/Lo-X	Source	MDCT	MTP M/D	Beast M/D	Author
1011	SVK_Prim/12_5000	FJ392912	BP	-	SVK	-	NCBI	--C-	-/-	-/-	(46)
1012	GBR_Prim/13_3370	FJ392913	BP	-	GBR	-	NCBI	--C-	-/-	-/-	(46)

Mitogenomes – this study (T-S):

No. 1-385 Fasta name = code of country of breed origin_abrev. of breed/species_order_MTP haplogroup classification_source.

Mitogenomes – NCBI:

No. 386-694 Fasta name = code of country of breed origin_abrev. of breed/species_order_MTP haplogroup classification_source_radiocarbon time (yBP).

(Exception is CHN_Prim_001_10560 without MTP haplogroup classification).

Retrieved mitogenomes from the publicly available whole genome sequences (rWGS):

No. 695-810 Fasta name = code of country of breed origin_abrev. of breed_order_MTP haplogroup classification_source.

Ancient D-loop sequences – NCBI and DDBJ:

No. 811-999 Fasta name = species (T=taurus, P=primigenius)_order_code of country_ radiocarbon time (yBP).

(Exceptions are: a) DEU_Prim_001_5800 where fasta name is described as = code of country_ species_order_ radiocarbon time (yBP). b) CHN_Qin_001_0_1 where fasta name is described as = code of country_breed_order_ radiocarbon time (yBP)_source).

Ancient CYTB sequences – NCBI:

No. 1000-1012 Fasta name = code of country_species_order_ radiocarbon time (yBP).

Source within the Fasta name represents: 1 = NCBI, 2 = this study, 3-4 = mitogenomes retrieved from whole genome sequences.

T-S = this study.

SP: BT = Bos taurus, BP = Bos primigenius, BI = Bos indicus, BB = Bison bison, BPc = Bison priscus, BS = Bos species.

ORG = represents sampling origin using ISO Alpha-3 Country Code + Kosovo (XKX). It is connected with "La-Y/Lo-X" column.

rWGS = mitogenomes retrieved from whole genome sequences.

MDCT – represents the usage of each sequence within the different dataset: M = mitogenome data set, D = D-loop data set, C = cytochrome b data set, T = time clock calibration data set.

MTP M/D = MitoToolPy haplogroup classification for M = mitogenome and D = D-loop.

BEAST M/D = BEAST haplogroup classification for M = mitogenome and D = D-loop.

Table S2. A detailed description of Median-Joining network haplotypes.

No.	Fasta name	Acc. no.	Haplotype	SP	Breed	ORG	MTP/BEAST	Author
1	HRV_Bosk_031_T3r_2	MZ901598	1	BT	Boskarin (Istrian Cattle)	HRV	T3r/T3	T-S
2	GBR_BRS_003_T3r_1	V00654	1	BT	-	GBR	T3r/T3	Anderson et al. 1982
3	CHN_Taur_001_I1x_1	KP143771	2	BT	-	CHN	I1/I	Liu et al. 2016
4	IRQ_Iraq_008_I1x_1	EU177868	3	BT	Iraqi	IRQ	I1/I	Achilli et al. 2008
5	CHN_Brah_001_I2_1*	MN200875	4	BT	Brahman	CHN	I2/-	Xia et. al 2019
6	IRN_Iran_007_I2x_1	EU177870	5	BT	Iranian	IRN	I2/I	Achilli et al. 2008
7	IRQ_Iraq_009_I2x_1	EU177869	6	BT	Iraqi	IRQ	I2/I	Achilli et al. 2008
8	CHN_Tibe_005_1*	MT576796	7	BT	Tibetan breed	CHN	T1'2'3'6'7/-	Xia et. al 2021
9	CHN_Tibe_006_1*	MT576797	7	BT	Tibetan breed	CHN	T1'2'3'6'7/-	Xia et. al 2021
10	DNK_Prim_001_P1_1_600	MF169211	8	BP	-	DNK	P1/P	Bro-Jorgensen et al. 2018
11	DNK_Prim_002_P1_1_600	MF169212	9	BP	-	DNK	P1/P	Bro-Jorgensen et al. 2018
12	AUT_Murb_003_P1x_2	MZ901408	10	BT	Murbodner	AUT	P1/P	T-S
13	AUT_Murb_005_P1x_2	MZ901404	10	BT	Murbodner	AUT	P1/P	T-S
14	AUT_Murb_006_P1x_2	MZ901405	10	BT	Murbodner	AUT	P1/P	T-S
15	AUT_Murb_007_P1x_2	MZ901406	10	BT	Murbodner	AUT	P1/P	T-S
16	AUT_Murb_008_P1x_2	MZ901407	10	BT	Murbodner	AUT	P1/P	T-S
17	GBR_Prim_004_P1x_1_6738	GU985279	11	BP	-	GBR	P1/P	Edwards et al. 2010
18	POL_Prim_003_P1x_1_1500	JQ437479	12	BP	-	POL	P1/P	Lipinski D.
19	JPN_Jsho_001_P2_1*	LC537308	13	BT	Japanese Shorthorn	JPN	P2/-	Mannen et al. 2020
20	CHN_Yanb_002_P2_1*	MT576841	14	BT	Yanbian	CHN	P2/-	Xia et. al 2021
21	JPN_Jsho_002_P2_1*	LC537309	14	BT	Japanese Shorthorn	JPN	P2/-	Mannen et al. 2020
22	JPN_Jsho_003_P2_1*	LC537310	15	BT	Japanese Shorthorn	JPN	P2/-	Mannen et al. 2020
23	JPN_Jsho_008_P2_1*	LC537315	15	BT	Japanese Shorthorn	JPN	P2/-	Mannen et al. 2020
24	JPN_Jsho_004_P2_1*	LC537311	16	BT	Japanese Shorthorn	JPN	P2/-	Mannen et al. 2020
25	JPN_Jsho_010_P2_1*	LC537317	16	BT	Japanese Shorthorn	JPN	P2/-	Mannen et al. 2020
26	JPN_Jsho_005_P2_1*	LC537312	17	BT	Japanese Shorthorn	JPN	P2/-	Mannen et al. 2020

No.	Fasta name	Acc. no.	Haplotype	SP	Breed	ORG	MTP/BEAST	Author
27	JPN_Jsho_006_P2_1*	LC537313	18	BT	Japanese Shorthorn	JPN	P2/-	Mannen et al. 2020
28	JPN_Jsho_007_P2_1*	LC537314	19	BT	Japanese Shorthorn	JPN	P2/-	Mannen et al. 2020
29	JPN_Jsho_009_P2_1*	LC537316	20	BT	Japanese Shorthorn	JPN	P2/-	Mannen et al. 2020
30	KOR_Beef_016_P2x_1	DQ124389	21	BT	Beef cattle	KOR	P2/P	Shin et al.
31	ARM_Prim_001_Q_3_7040*	SAMEA5577362	22	BP	-	ARM	Q/-	Verdugo et al. 2019
32	ISR_Taur_007_Q1_3_2820*	SAMEA5577407	23	BT	-	ISR	Q1/-	Verdugo et al. 2019
33	TUR_Taur_005_Q1_3_7920*	SAMEA5577377	24	BT	-	TUR	Q1/-	Verdugo et al. 2019
34	ITA_Roma_002_Q1a_1	FJ971083	25	BT	Romagnola	ITA	Q1a/Q	Achilli et al. 2009
35	ITA_Roma_005_Q1a_1	HQ184034	25	BT	Romagnola	ITA	Q1a/Q	Bonfiglio et al. 2010
36	ITA_Roma_006_Q1a_1	HQ184035	25	BT	Romagnola	ITA	Q1a/Q	Bonfiglio et al. 2010
37	EGY_Domi_013_Q1x_1	KT184471	26	BT	Domiaty	EGY	Q1/Q	Olivieri et al. 2015
38	GRC_Kate_004_Q1x_2	MZ901639	27	BT	Katerini	GRC	Q1/Q	T-S
39	GRC_ShoP_002_Q1x_2	MZ901648	27	BT	Greece Shorthorn Pindos	GRC	Q1/Q	T-S
40	ITA_Caba_006_Q1x_1	HQ184038	28	BT	Cabannina	ITA	Q1/Q	Bonfiglio et al. 2010
41	ITA_Chia_017_Q1x_1	HQ184039	29	BT	Chianina	ITA	Q1/Q	Bonfiglio et al. 2010
42	SPA_Pire_001_Q1x_1	KP637147	30	BT	Pirenaica	ESP	Q1/Q	Lopez-Oceja et al. 2016
43	ITA_Chia_015_Q2x_1	HQ184031	31	BT	Chianina	ITA	Q2/Q	Bonfiglio et al. 2010
44	ITA_Roma_001_Q2x_1	FJ971080	32	BT	Romagnola	ITA	Q2/Q	Achilli et al. 2009
45	ITA_Roma_004_Q2x_1	HQ184033	32	BT	Romagnola	ITA	Q2/Q	Bonfiglio et al. 2010
46	ITA_Cini_003_R1a_1	FJ971085	33	BT	Cinisara	ITA	R1a/R	Achilli et al. 2009
47	ITA_Cini_004_R1a_1	FJ971086	33	BT	Cinisara	ITA	R1a/R	Achilli et al. 2009
48	ITA_Roma_007_R1x_1	HQ184040	34	BT	Romagnola	ITA	R1/R	Bonfiglio et al. 2010
49	ITA_Roma_008_R1x_1	HQ184041	34	BT	Romagnola	ITA	R1/R	Bonfiglio et al. 2010
50	ITA_Roma_009_R1x_1	HQ184042	34	BT	Romagnola	ITA	R1/R	Bonfiglio et al. 2010
51	ITA_Roma_010_R1x_1	HQ184043	34	BT	Romagnola	ITA	R1/R	Bonfiglio et al. 2010
52	ITA_Roma_011_R1x_1	HQ184044	34	BT	Romagnola	ITA	R1/R	Bonfiglio et al. 2010
53	MAR_Prim_001_R2_3_7993*	SAMEA5577395	35	BP	-	MAR	R2/-	Verdugo et al. 2019
54	ITA_Roma_003_R2x_1	FJ971087	36	BT	Romagnola	ITA	R2/R	Achilli et al. 2009

No.	Fasta name	Acc. no.	Haplotype	SP	Breed	ORG	MTP/BEAST	Author
55	ITA_Marc_001_R2x_1	HQ184045	36	BT	Marchigiana	ITA	R2/R	Bonfiglio et al. 2010
56	ETH_Arsi_002_T1a_1	JN817303	37	BI	Arsi	ETH	T1a/T1	Bonfiglio et al. 2012
57	HRV_BuHr_008_T1a_2	MZ901461	38	BT	Croatia Buša	HRV	T1a/T3	T-S
58	HRV_BuHr_028_T1a_2	MZ901447	38	BT	Croatia Buša	HRV	T1a/T3	T-S
59	ITA_Chia_009_T1a_1	EU177846	39	BT	Chianina	ITA	T1a/T1	Achilli et al. 2008
60	ITA_Cini_005_T1a_1	JN817317	40	BT	Cinisara	ITA	T1a/T1	Bonfiglio et al. 2012
61	ITA_Cini_006_T1a_1	JN817318	41	BT	Cinisara	ITA	T1a/T1	Bonfiglio et al. 2012
62	ITA_Marc_003_T1a_1	JN817335	42	BT	Marchigiana	ITA	T1a/T1	Bonfiglio et al. 2012
63	ITA_Marc_004_T1a_1	JN817336	43	BT	Marchigiana	ITA	T1a/T1	Bonfiglio et al. 2012
64	ITA_Marc_005_T1a_1	JN817337	44	BT	Marchigiana	ITA	T1a/T1	Bonfiglio et al. 2012
65	ITA_Marc_006_T1a_1	JN817338	45	BT	Marchigiana	ITA	T1a/T1	Bonfiglio et al. 2012
66	ITA_Marc_007_T1a_1	JN817339	46	BT	Marchigiana	ITA	T1a/T1	Bonfiglio et al. 2012
67	ITA_Marm_003_T1a_1	EU177844	47	BT	Maremmana	ITA	T1a/T1	Achilli et al. 2008
68	ITA_Marm_004_T1a_1	JN817332	48	BT	Maremmana	ITA	T1a/T1	Bonfiglio et al. 2012
69	ITA_Marm_005_T1a_1	JN817333	49	BT	Maremmana	ITA	T1a/T1	Bonfiglio et al. 2012
70	ITA_Podo_010_T1a_1	EU177843	50	BT	Italian Podolian	ITA	T1a/T1	Achilli et al. 2008
71	ITA_Podo_012_T1a_1	JN817344	51	BT	Italian Podolian	ITA	T1a/T1	Bonfiglio et al. 2012
72	ITA_Roma_012_T1a_1	JN817345	52	BT	Romagnola	ITA	T1a/T1	Bonfiglio et al. 2012
73	ITA_Roma_014_T1a_1	JN817347	53	BT	Romagnola	ITA	T1a/T1	Bonfiglio et al. 2012
74	PRT_Barr_003_T1a_2	MZ901386	54	BT	Barrosa	PRT	T1a/T1	T-S
75	AFR_Ngun_010_T1b_1	KF163070	55	BT	Nguni	ZAF	T1b/T1	Horsburgh et al. 2013
76	ALB_Pres_001_T1b_2	MZ901716	56	BT	Prespa Cattle	ALB	T1b/T3	T-S
77	EGY_Menf_003_T1b_1	JN817327	57	BT	Menofi	EGY	T1b/T3	Bonfiglio et al. 2012
78	ETH_Bora_002_T1b_3	SRS1429491	58	BI	Boran	ETH	T1b/T1	Kim et al. 2017
79	ISR_Taur_005_T1b_3_4800*	SAMEA5577402	59	BT	-	ISR	T1b/-	Verdugo et al. 2019
80	ITA_Cini_002_T1b_1	EU177842	60	BT	Cinisara	ITA	T1b/T1	Achilli et al. 2008
81	ITA_Cini_008_T1b_1	JN817320	61	BT	Cinisara	ITA	T1b/T1	Bonfiglio et al. 2012
82	ITA_Marc_002_T1b_1	JN817334	62	BT	Marchigiana	ITA	T1b/T1	Bonfiglio et al. 2012

No.	Fasta name	Acc. no.	Haplotype	SP	Breed	ORG	MTP/BEAST	Author
83	ITA_Marc_008_T1b_1	JN817351	63	BT	Marchigiana	ITA	T1b/T1	Bonfiglio et al. 2012
84	NLD_HoFr_063_T1b_2	MZ901569	64	BT	Holstein	HRV	T1b/T3	T-S
85	SDN_Kena_005_T1b_3	SRS1439367	65	BI	Kenana	SDN	T1b/T1	Kim et al. 2017
86	EGY_Domi_012_T1c_1	KT184470	66	BT	Domiaty	EGY	T1c/T3	Olivieri et al. 2015
87	EGY_Menf_004_T1c_1	JN817328	67	BT	Menofi	EGY	T1c/T1	Bonfiglio et al. 2012
88	ITA_Cini_007_T1c_1	JN817319	68	BT	Cinisara	ITA	T1c/T3	Bonfiglio et al. 2012
89	ITA_Roma_013_T1c_1	JN817346	69	BT	Romagnola	ITA	T1c/T1	Bonfiglio et al. 2012
90	KOR_Kore_001_T1c_1	AY526085	70	BT	Korean native	KOR	T1c/T4	Chung et al.
91	KOR_Nati_001_T1c_1	NC_006853	70	BT	Korean native	KOR	T1c/T4	Chung et al.
92	PRT_Alen_001_T1c_1	JN817300	71	BT	Alentejana	PRT	T1c/T1	Bonfiglio et al. 2012
93	GBR_WPar_002_T1e_1	KC153973	72	BT	White Park	GBR	T1e/T3	Ludwig et al. 2013
94	GBR_WPar_004_T1e_1	KC153975	72	BT	White Park	GBR	T1e/T3	Ludwig et al. 2013
95	HRV_Bosk_038_T3x_2	MZ901605	73	BT	Boskarin (Istrian Cattle)	HRV	T3/T3	T-S
96	ITA_Calv_001_T1e_1	JN817306	73	BT	Calvana	ITA	T1e/T3	Bonfiglio et al. 2012
97	GRC_Taur_002_T3r_3_7600*	SAMEA5577391	73	BT	-	GRC	T3r/-	Verdugo et al. 2019
98	TUR_Prim_004_T3_3_7600*	SAMEA5577358	73	BP	-	TUR	T3/-	Verdugo et al. 2019
99	TUR_Taur_003_T3_3_8050*	SAMEA5577376	73	BT	-	TUR	T3/-	Verdugo et al. 2019
100	ITA_Chia_007_T1e_1	EU177841	74	BT	Chianina	ITA	T1e/T3	Achilli et al. 2008
101	HRV_BuHr_009_T1f_2	MZ901462	75	BT	Croatia Buša	HRV	T1f/T1	T-S
102	ITA_Podo_011_T1f_1	JN817343	76	BT	Italian Podolian	ITA	T1f/T1	Bonfiglio et al. 2012
103	CHN_Yanb_001_T1f_1*	MT576711	77	BT	Yanbian	CHN	T1f/-	Xia et. al 2021
104	CHN_Yanb_003_T1f_1*	MT576842	78	BT	Yanbian	CHN	T1f/-	Xia et. al 2021
105	CHN_Yanb_004_T1f_1*	MT576843	78	BT	Yanbian	CHN	T1f/-	Xia et. al 2021
106	ESP_Saya_002_T1x_2	MZ901749	79	BT	Sayaguesa	DEU	T1/T3	T-S
107	GRC_ShoR_004_T1x_2	MZ901654	80	BT	Greece Shorthorn Rodopi	GRC	T1/T1	T-S
108	HRV_Bosk_028_T1x_2	MZ901595	81	BT	Boskarin (Istrian Cattle)	HRV	T1/T3	T-S
109	HRV_Bosk_029_T1x_2	MZ901596	81	BT	Boskarin (Istrian Cattle)	HRV	T1/T3	T-S
110	HRV_BuHr_001_T1x_2	MZ901433	82	BT	Croatia Buša	HRV	T1/T3	T-S

No.	Fasta name	Acc. no.	Haplotype	SP	Breed	ORG	MTP/BEAST	Author
111	MKD_BuMd_001_T1x_2	MZ901703	83	BT	Macedonian Buša	MKD	T1/T1	T-S
112	IRN_Taur_004_T2_3_4760*	SAMEA5577389	84	BT	-	IRN	T2/-	Verdugo et al. 2019
113	IRN_Taur_005_T2_3_4470*	SAMEA5577364	85	BT	-	IRN	T2/-	Verdugo et al. 2019
114	IRQ_Taur_001_T2_3_2800*	SAMEA5577351	86	BT	-	IRQ	T2/-	Verdugo et al. 2019
115	SRB_Taur_003_T2_3_7000*	SAMEA5577383	87	BT	-	SRB	T2/-	Verdugo et al. 2019
116	GRC_KeaR_003_T2x_2	MZ901693	88	BT	Kea Rind	GRC	T2/T2	T-S
117	TUR_Bspe_002_T2_3_8024*	SAMEA5577393	88	Bspecies	-	TUR	T2/-	Verdugo et al. 2019
118	GRC_Pres_007_T2x_2	MZ901645	89	BT	Greece Prespa	GRC	T2/T2	T-S
119	HRV_BuHr_010_T2x_2	MZ901463	90	BT	Croatia Buša	HRV	T2/T2	T-S
120	KOS_BuDi_003_T2x_2	MZ901671	91	BT	Dukagjini Buša	XKX	T2/T2	T-S
121	MNG_Tura_004_T2x_2	MZ901758	92	BT	Turano-Mongolian	MNG	T2/T2	T-S
122	SRB_Taur_012_T3_3_7025*	SAMEA5577382	93	BT	-	SRB	T3/-	Verdugo et al. 2019
123	NLD_HoFr_016_T3a_1	DQ124418	94	BT	Holstein	NLD	T3a/T4	Shin et al.
124	HRV_Bosk_001_T3c_2	MZ901608	95	BT	Boskarin (Istrian Cattle)	HRV	T3c/T3	T-S
125	HRV_Bosk_019_T3c_2	MZ901586	95	BT	Boskarin (Istrian Cattle)	HRV	T3c/T3	T-S
126	HRV_Bosk_020_T3c_2	MZ901587	95	BT	Boskarin (Istrian Cattle)	HRV	T3c/T3	T-S
127	HRV_Bosk_022_T3c_2	MZ901589	95	BT	Boskarin (Istrian Cattle)	HRV	T3c/T3	T-S
128	HRV_Bosk_040_T3c_2	MZ901607	95	BT	Boskarin (Istrian Cattle)	HRV	T3c/T3	T-S
129	ESP_Saya_003_T3d_2	MZ901750	96	BT	Sayaguesa	DEU	T3d/T3	T-S
130	ITA_Chia_004_T3i_1	EU177822	97	BT	Chianina	ITA	T3i/T3	Achilli et al. 2008
131	ITA_Cini_001_T3i_1	EU177823	98	BT	Cinisara	ITA	T3i/T3	Achilli et al. 2008
132	AUT_Simm_001_T3j_2	MZ901420	99	BT	Fleckvieh	AUT	T3j/T3	T-S
133	HRV_Bosk_027_T3j_2	MZ901594	100	BT	Boskarin (Istrian Cattle)	HRV	T3j/T3	T-S
134	HRV_Bosk_032_T3j_2	MZ901599	100	BT	Boskarin (Istrian Cattle)	HRV	T3j/T3	T-S
135	ITA_Marm_002_T3j_1	EU177829	101	BT	Maremmana	ITA	T3j/T3	Achilli et al. 2008
136	ITA_Podo_009_T3j_1	EU177830	102	BT	Italian Podolian	ITA	T3j/T3	Achilli et al. 2008
137	IRQ_Iraq_003_T3k_1	EU177837	103	BT	Iraqi	IRQ	T3k/T3	Achilli et al. 2008
138	CHN_Anxi_002_T3k_1*	MT576715	104	BT	Anxi breed	CHN	T3k/-	Xia et. al 2021

No.	Fasta name	Acc. no.	Haplotype	SP	Breed	ORG	MTP/BEAST	Author
139	CHN_Tibe_003_T3k_1*	MT576748	105	BT	Tibetan breed	CHN	T3k/-	Xia et. al 2021
140	ESP_Betz_001_T3l_1	EU177833	106	BT	Betizuak	ESP	T3l/T3	Achilli et al. 2008
141	CHN_Tibe_001_T3n_1*	MT576730	107	BT	Tibetan breed	CHN	T3n/-	Xia et. al 2021
142	CHN_Tibe_002_T3n_1*	MT576746	108	BT	Tibetan breed	CHN	T3n/-	Xia et. al 2021
143	CHN_Tibe_004_T3n_1*	MT576755	109	BT	Tibetan breed	CHN	T3n/-	Xia et. al 2021
144	GRC_Pres_006_T3o_2	MZ901644	110	BT	Greece Prespa	GRC	T3o/T3	T-S
145	HRV_Bosk_023_T3o_2	MZ901590	111	BT	Boskarin (Istrian Cattle)	HRV	T3o/T3	T-S
146	IRQ_Taur_002_T3o_3_629*	SAMEA5577352	112	BT	-	IRQ	T3o/-	Verdugo et al. 2019
147	HRV_Bosk_033_T3p_2	MZ901600	113	BT	Boskarin (Istrian Cattle)	HRV	T3p/T3	T-S
148	ITA_Marm_001_T3p_1	EU177824	114	BT	Maremmana	ITA	T3p/T3	Achilli et al. 2008
149	DEU_MuWe_003_T3q_2	MZ901713	115	BT	Murnau-Werdenfelser	DEU	T3q/T3	T-S
150	HRV_Bosk_025_T3q_2	MZ901592	116	BT	Boskarin (Istrian Cattle)	HRV	T3q/T3	T-S
151	HRV_Bosk_026_T3q_2	MZ901593	116	BT	Boskarin (Istrian Cattle)	HRV	T3q/T3	T-S
152	HRV_Bosk_036_T3q_2	MZ901603	116	BT	Boskarin (Istrian Cattle)	HRV	T3q/T3	T-S
153	HRV_Bosk_037_T3q_2	MZ901604	116	BT	Boskarin (Istrian Cattle)	HRV	T3q/T3	T-S
154	ITA_Chia_006_T3q_1	EU177828	117	BT	Chianina	ITA	T3q/T3	Achilli et al. 2008
155	AUT_AtBr_002_T3r_2	MZ901411	118	BT	Original Austrian Brown	AUT	T3r/T3	T-S
156	AUT_Enns_001_T3r_2	MZ901394	119	BT	Ennstaler Bergschecken	AUT	T3r/T3	T-S
157	AUT_TuxZ_001_T3r_2	MZ901423	120	BT	Tux-Zillertaler	AUT	T3r/T3	T-S
158	BGR_ShoR_003_T3r_2	MZ901724	121	BT	Rhodopean Shorthorn	BGR	T3r/T3	T-S
159	DEU_Heck_001_T3r_1	HM045018	122	BT	Heck cattle	DEU	T3r/T3	Zeyland et al.
160	FRA_Char_001_T3r_1	AY676858	123	BT	Charolais	FRA	T3r/T3	Shahid et al.
161	GEO_Taur_003_T3r_3_1000*	SAMEA5577367	124	BT	-	GEO	T3r/-	Verdugo et al. 2019
162	GRC_Kate_003_T3r_2	MZ901638	125	BT	Katerini	GRC	T3r/T3	T-S
163	HRV_Bosk_003_T3r_2	MZ901610	126	BT	Boskarin (Istrian Cattle)	HRV	T3r/T3	T-S
164	HRV_Bosk_010_T3r_2	MZ901617	126	BT	Boskarin (Istrian Cattle)	HRV	T3r/T3	T-S
165	HRV_Bosk_024_T3r_2	MZ901591	126	BT	Boskarin (Istrian Cattle)	HRV	T3r/T3	T-S
166	HRV_Bosk_008_T3r_2	MZ901615	127	BT	Boskarin (Istrian Cattle)	HRV	T3r/T3	T-S

No.	Fasta name	Acc. no.	Haplotype	SP	Breed	ORG	MTP/BEAST	Author
167	HRV_Bosk_014_T3r_2	MZ901581	128	BT	Boskarin (Istrian Cattle)	HRV	T3r/T3	T-S
168	HRV_Bosk_021_T3r_2	MZ901588	129	BT	Boskarin (Istrian Cattle)	HRV	T3r/T3	T-S
169	HRV_Bosk_034_T3r_2	MZ901601	130	BT	Boskarin (Istrian Cattle)	HRV	T3r/T3	T-S
170	HRV_Podo_001_T3r_2	MZ901620	131	BT	Slavonian Syrmian Podolian	HRV	T3r/T3	T-S
171	HRV_Podo_009_T3r_2	MZ901630	131	BT	Slavonian Syrmian Podolian	HRV	T3r/T3	T-S
172	HUN_HunG_001_T3r_1	GQ129207	131	BT	Hungarian Grey	HUN	T3r/T3	Zeyland et al.
173	HRV_Podo_004_T3r_2	MZ901627	132	BT	Slavonian Syrmian Podolian	HRV	T3r/T3	T-S
174	HRV_Podo_007_T3r_2	MZ901631	132	BT	Slavonian Syrmian Podolian	HRV	T3r/T3	T-S
175	HRV_Podo_008_T3r_2	MZ901632	132	BT	Slavonian Syrmian Podolian	HRV	T3r/T3	T-S
176	HRV_Podo_011_T3r_2	MZ901634	132	BT	Slavonian Syrmian Podolian	HRV	T3r/T3	T-S
177	HRV_Podo_012_T3r_2	MZ901635	132	BT	Slavonian Syrmian Podolian	HRV	T3r/T3	T-S
178	HRV_Podo_016_T3r_2	MZ901624	132	BT	Slavonian Syrmian Podolian	HRV	T3r/T3	T-S
179	IRN_Taur_006_T3r_3_2900*	SAMEA5577365	133	BT	-	IRN	T3r/-	Verdugo et al. 2019
180	RUS_Jaku_002_T3r_2	MZ901733	134	BT	Yakutian	RUS	T3r/T3	T-S
181	SRB_BuSr_002_T3r_2	MZ901745	135	BT	Serbian Buša	SRB	T3r/T3	T-S
182	SVN_Cika_001_T3r_2	MZ901657	136	BT	Cika	SVN	T3r/T3	T-S
183	SVN_Cika_003_T3r_2	MZ901659	137	BT	Cika	SVN	T3r/T3	T-S
184	UKR_UkrG_001_T3r_1	GQ129208	138	BT	Ukrainian Grey	UKR	T3r/T3	Zeyland et al.
185	ALB_BuLe_003_T3s_2	MZ901697	139	BT	Lekbibaj Buša	ALB	T3s/T3	T-S
186	AUT_Pinz_002_T3s_2	MZ901413	140	BT	Original Pinzgauer	AUT	T3s/T3	T-S
187	ALB_BuDb_004_T3x_2	MZ901668	141	BT	Dibra Buša	ALB	T3/T3	T-S
188	AUT_VieB_002_T3x_2	MZ901399	142	BT	Karntner Blondvieh	AUT	T3/T3	T-S
189	AUT_WalB_002_T3x_2	MZ901428	143	BT	Waldviertler Blondvieh	AUT	T3/T3	T-S
190	BGR_ShoR_008_T3x_2	MZ901729	144	BT	Rhodopean Shorthorn	BGR	T3/T3	T-S
191	DEU_VieG_004_T3x_2	MZ901676	145	BT	Gelbvieh (German Yellow)	DEU	T3/T3	T-S
192	GRC_ShoP_003_T3x_2	MZ901649	146	BT	Greece Shorthorn Pindos	GRC	T3/T3	T-S
193	HRV_Bosk_002_T3x_2	MZ901609	147	BT	Boskarin (Istrian Cattle)	HRV	T3/T3	T-S
194	HRV_Bosk_009_T3x_2	MZ901616	147	BT	Boskarin (Istrian Cattle)	HRV	T3/T3	T-S

No.	Fasta name	Acc. no.	Haplotype	SP	Breed	ORG	MTP/BEAST	Author
195	HRV_Bosk_011_T3x_2	MZ901618	147	BT	Boskarin (Istrian Cattle)	HRV	T3/T3	T-S
196	HRV_Bosk_004_T3x_2	MZ901611	148	BT	Boskarin (Istrian Cattle)	HRV	T3/T3	T-S
197	HRV_Bosk_035_T3x_2	MZ901602	148	BT	Boskarin (Istrian Cattle)	HRV	T3/T3	T-S
198	HRV_Bosk_005_T3x_2	MZ901612	149	BT	Boskarin (Istrian Cattle)	HRV	T3/T3	T-S
199	HRV_Bosk_006_T3x_2	MZ901613	150	BT	Boskarin (Istrian Cattle)	HRV	T3/T3	T-S
200	HRV_Bosk_030_T3x_2	MZ901597	150	BT	Boskarin (Istrian Cattle)	HRV	T3/T3	T-S
201	HRV_Bosk_039_T3x_2	MZ901606	150	BT	Boskarin (Istrian Cattle)	HRV	T3/T3	T-S
202	HRV_Bosk_007_T3x_2	MZ901614	151	BT	Boskarin (Istrian Cattle)	HRV	T3/T3	T-S
203	HRV_Bosk_013_T3x_2	MZ901580	151	BT	Boskarin (Istrian Cattle)	HRV	T3/T3	T-S
204	HRV_Bosk_016_T3x_2	MZ901583	151	BT	Boskarin (Istrian Cattle)	HRV	T3/T3	T-S
205	HRV_Bosk_012_T3x_2	MZ901619	152	BT	Boskarin (Istrian Cattle)	HRV	T3/T3	T-S
206	HRV_Bosk_015_T3x_2	MZ901582	153	BT	Boskarin (Istrian Cattle)	HRV	T3/T3	T-S
207	HRV_Bosk_017_T3x_2	MZ901584	154	BT	Boskarin (Istrian Cattle)	HRV	T3/T3	T-S
208	HRV_Bosk_018_T3x_2	MZ901585	155	BT	Boskarin (Istrian Cattle)	HRV	T3/T3	T-S
209	HRV_Podo_002_T3x_2	MZ901625	156	BT	Slavonian Syrmian Podolian	HRV	T3/T3	T-S
210	HRV_Podo_003_T3x_2	MZ901626	156	BT	Slavonian Syrmian Podolian	HRV	T3/T3	T-S
211	HRV_Podo_005_T3x_2	MZ901628	157	BT	Slavonian Syrmian Podolian	HRV	T3/T3	T-S
212	HRV_Podo_006_T3x_2	MZ901629	157	BT	Slavonian Syrmian Podolian	HRV	T3/T3	T-S
213	HRV_Podo_014_T3x_2	MZ901622	157	BT	Slavonian Syrmian Podolian	HRV	T3/T3	T-S
214	MNE_BuCG_002_T3x_2	MZ901709	158	BT	Montenegro Buša	MNE	T3/T3	T-S
215	CHN_Yung_002_T4_1*	MN200841	159	BT	Yunling	CHN	T4/-	Xia et. al 2019
216	CHN_Yung_003_T4_1*	MN200842	159	BT	Yunling	CHN	T4/-	Xia et. al 2019
217	CHN_Yung_005_T4_1*	MN200848	159	BT	Yunling	CHN	T4/-	Xia et. al 2019
218	CHN_Yung_008_T4_1*	MN200937	159	BT	Yunling	CHN	T4/-	Xia et. al 2019
219	JPN_JaBl_002_T4a_1	AB074963	160	BT	Japanese Black	JPN	T4a/T4	Mannen et al. 2003
220	KOR_Kore_003_T4a_1	DQ124372	161	BT	Korean cattle	KOR	T4a/T4	Shin et al.
221	KOR_Kore_006_T4a_1	DQ124375	162	BT	Korean cattle	KOR	T4a/T4	Shin et al.
222	CHN_Yung_001_T4a_1*	MN200795	163	BT	Yunling	CHN	T4a/-	Xia et. al 2019

No.	Fasta name	Acc. no.	Haplotype	SP	Breed	ORG	MTP/BEAST	Author
223	CHN_Yung_006_T4a_1*	MN200860	163	BT	Yunling	CHN	T4a/-	Xia et. al 2019
224	ALB_Tief_002_T4x_2	MZ901380	164	BT	Middle-Albanian Buša	ALB	T4/T4	T-S
225	JPN_JaBl_001_T4x_1	AB074962	165	BT	Japanese Black	JPN	T4/T4	Mannen et al. 2003
226	JPN_JaBl_003_T4x_1	AB074964	166	BT	Japanese Black	JPN	T4/T4	Mannen et al. 2003
227	KOR_Beef_005_T4x_1	DQ124392	167	BT	Beef cattle	KOR	T4/T4	Shin et al.
228	KOR_Beef_013_T4x_1	DQ124400	168	BT	Beef cattle	KOR	T4/T4	Shin et al.
229	KOR_Beef_014_T4x_1	DQ124401	169	BT	Beef cattle	KOR	T4/T4	Shin et al.
230	KOR_Kore_008_T4x_1	DQ124377	170	BT	Korean cattle	KOR	T4/T4	Shin et al.
231	NLD_HoFr_010_T4x_1	DQ124412	171	BT	Holstein	NLD	T4/T4	Shin et al.
232	ITA_Pied_002_T5a_1	EU177863	172	BT	Piedmontese	ITA	T5a/T5	Achilli et al. 2008
233	ITA_Vald_002_T5a_1	EU177862	173	BT	Valdostana	ITA	T5a/T5	Achilli et al. 2008
234	HRV_Podo_010_T5b_2	MZ901633	174	BT	Slavonian Syrmian Podolian	HRV	T5b/T5	T-S
235	HRV_Podo_013_T5b_2	MZ901621	174	BT	Slavonian Syrmian Podolian	HRV	T5b/T5	T-S
236	HRV_Podo_015_T5b_2	MZ901623	174	BT	Slavonian Syrmian Podolian	HRV	T5b/T5	T-S
237	IRQ_Iraq_006_T5b_1	EU177864	175	BT	Iraqi	IRQ	T5b/T5	Achilli et al. 2008
238	IRQ_Iraq_007_T5b_1	EU177865	176	BT	Iraqi	IRQ	T5b/T5	Achilli et al. 2008
239	ALB_BuSk_001_T5x_2	MZ901740	177	BT	Skodra Buša	ALB	T5/T5	T-S
240	ALB_BuSk_004_T5x_2	MZ901743	177	BT	Skodra Buša	ALB	T5/T5	T-S
241	NLD_HoFr_120_T5x_2	MZ901530	178	BT	Holstein	HRV	T5/T5	T-S
242	GBR_AngX_006_T6x_1	AY676867	179	BT	Angus X	GBR	T6/T3	Shahid et al.
243	GBR_AngX_009_T6x_1	AY676870	179	BT	Angus X	GBR	T6/T3	Shahid et al.
244	GBR_Angu_022_T6x_3	SRS629688	179	BT	Angus	CAN	T6/T3	Stothard et al. 2015
245	CHN_Yung_004_T6_1*	MN200846	179	BT	Yunling	CHN	T6/-	Xia et. al 2019
246	CHN_Yung_007_T6_1*	MN200896	179	BT	Yunling	CHN	T6/-	Xia et. al 2019
247	ITA_Caba_001_T7x_1	EU177840	180	BT	Cabannina	ITA	T7/T3	Achilli et al. 2008

The meaning of the abbreviations of the fasta names is the same as in the description in Table 1. For the Median joining network, 47 new sequences above 200 from Table 1 were added and marked with "*". Mitogenome sequences from the Verdugo et al. 2019 were downloaded in the form of fasta fila and retrieved from the genome following their protocols.

Table S3. Primers, multiplication positions, and the size of multiplied fragments.

	Primers	(bp)	(bp)	Fragment size (bp)
MitoBt1F	ccgtcaccctcctcaaatag	1272	7904	6631
MitoBt1R	ctgggattgcgtctgttttt	7904	1272	
MitoBt2F	gatgccctccaccatatcac	7197	13676	6498
MitoBt2R	gaacttgaaggcgtttgagg	13676	7197	
MitoBt3F	tacacgggaaaatgcactca	11819	2322	6803
MitoBt3R	ggaatgctggaggtgatgtt	2322	11819	

Table S4. Reagents and Conditions of PCR Reaction.

Reagents	µl	PCR	º C	min'/ sec"
GoTaqLong PCR Master Mix	12.5	Initial denaturation	94	2'
Primer upstream (10 µM)	1.8	35 cycles:		
Primer downstream (10 µM)	1.8	Denaturation	94	30"
H_2O	7.9	Annealing	58	30"
DNA	1.0	Elongation	72	6'
		Elongation	72	10'
Total	25.0			

Table S5. Quality control statistics of the mitogenome sequences derived in this study and from rWGS (mitogenomes retrieved from whole genome sequences). Fasta name abbreviation is the same as defined in Table S1.

No.	Fasta name	Acc. no.	Source	Avg. fold	Covered percent	Length
1	ALB_BuAl_001_T3r_2	MZ901685	T-S	3372.14	100.00	16340
2	ALB_BuAl_002_T3d_2	MZ901686	T-S	3000.53	100.00	16340
3	ALB_BuDb_001_T3h_2	MZ901665	T-S	1189.78	100.00	16339
4	ALB_BuDb_002_T3r_2	MZ901666	T-S	3732.91	100.00	16340
5	ALB_BuDb_003_T3r_2	MZ901667	T-S	2485.51	100.00	16340
6	ALB_BuDb_004_T3x_2	MZ901668	T-S	2452.22	100.00	16338
7	ALB_BuLe_001_T3r_2	MZ901695	T-S	2362.55	100.00	16340
8	ALB_BuLe_002_T3r_2	MZ901696	T-S	3657.53	100.00	16340
9	ALB_BuLe_003_T3s_2	MZ901697	T-S	2118.68	100.00	16338
10	ALB_BuLe_004_T3n_2	MZ901698	T-S	3199.37	100.00	16338
11	ALB_BuSk_001_T5x_2	MZ901740	T-S	8556.69	100.00	16338
12	ALB_BuSk_002_T3x_2	MZ901741	T-S	7080.03	100.00	16340
13	ALB_BuSk_003_T3x_2	MZ901742	T-S	9079.02	100.00	16339
14	ALB_BuSk_004_T5x_2	MZ901743	T-S	9180.14	100.00	16338
15	ALB_Pres_001_T1b_2	MZ901716	T-S	10121.05	100.00	16340
16	ALB_Pres_002_T3r_2	MZ901717	T-S	8723.28	100.00	16340
17	ALB_Pres_003_T3g_2	MZ901718	T-S	9852.50	100.00	16339
18	ALB_Pres_004_T3r_2	MZ901719	T-S	6609.57	100.00	16340
19	ALB_Tief_001_T3r_2	MZ901379	T-S	2987.31	100.00	16340
20	ALB_Tief_002_T4x_2	MZ901380	T-S	3072.51	100.00	16339
21	AUT_AtBr_001_T3x_2	MZ901410	T-S	6855.03	100.00	16340
22	AUT_AtBr_002_T3r_2	MZ901411	T-S	6643.01	100.00	16340
23	AUT_Enns_001_T3r_2	MZ901394	T-S	4007.53	100.00	16341
24	AUT_Enns_002_T3o_2	MZ901395	T-S	4484.74	100.00	16340
25	AUT_Enns_003_T3b_2	MZ901396	T-S	4294.91	100.00	16338
26	AUT_Enns_004_T3b_2	MZ901397	T-S	4192.49	100.00	16339
27	AUT_Murb_001_T3q_2	MZ901402	T-S	3057.12	100.00	16339
28	AUT_Murb_002_T3b_2	MZ901403	T-S	3192.34	100.00	16338

No.	Fasta name	Acc. no.	Source	Avg. fold	Covered_percent	Length
29	AUT_Murb_003_P1x_2	MZ901408	T-S	3802.40	100.00	16340
30	AUT_Murb_004_T3r_2	MZ901409	T-S	4275.65	100.00	16340
31	AUT_Murb_005_P1x_2	MZ901404	T-S	2292.79	100.00	16338
32	AUT_Murb_006_P1x_2	MZ901405	T-S	870.40	100.00	16338
33	AUT_Murb_007_P1x_2	MZ901406	T-S	2002.66	100.00	16338
34	AUT_Murb_008_P1x_2	MZ901407	T-S	2063.20	100.00	16338
35	AUT_Pinz_001_T3b_2	MZ901412	T-S	3966.98	100.00	16340
36	AUT_Pinz_002_T3s_2	MZ901413	T-S	4216.47	100.00	16339
37	AUT_Pinz_003_T3b_2	MZ901414	T-S	3888.42	100.00	16340
38	AUT_Pinz_004_T3k_2	MZ901415	T-S	3598.33	100.00	16339
39	AUT_Pust_001_T3x_2	MZ901416	T-S	3791.52	100.00	16341
40	AUT_Pust_002_T3r_2	MZ901417	T-S	4171.81	100.00	16340
41	AUT_Pust_003_T3p_2	MZ901418	T-S	4402.31	100.00	16339
42	AUT_Pust_004_T3r_2	MZ901419	T-S	4533.98	100.00	16340
43	AUT_Simm_001_T3j_2	MZ901420	T-S	4541.99	100.00	16339
44	AUT_Simm_002_T3x_2	MZ901421	T-S	2633.06	100.00	16339
45	AUT_Simm_003_T3x_2	MZ901422	T-S	3849.09	100.00	16338
46	AUT_TuxZ_001_T3r_2	MZ901423	T-S	7213.22	100.00	16339
47	AUT_TuxZ_002_T3r_2	MZ901424	T-S	6358.17	100.00	16339
48	AUT_TuxZ_003_T3r_2	MZ901425	T-S	7144.53	100.00	16342
49	AUT_TuxZ_004_T3q_2	MZ901426	T-S	8797.83	100.00	16339
50	AUT_VieB_001_T3x_2	MZ901398	T-S	4831.58	100.00	16338
51	AUT_VieB_002_T3x_2	MZ901399	T-S	7901.99	100.00	16338
52	AUT_VieB_003_T3r_2	MZ901400	T-S	5036.22	100.00	16339
53	AUT_VieB_004_T3x_2	MZ901401	T-S	5943.96	100.00	16339
54	AUT_WalB_001_T3q_2	MZ901427	T-S	3402.86	100.00	16340
55	AUT_WalB_002_T3x_2	MZ901428	T-S	3225.43	100.00	16340
56	AUT_WalB_003_T3x_2	MZ901429	T-S	4144.64	100.00	16338
57	AUT_WalB_004_T3x_2	MZ901430	T-S	1771.74	100.00	16339
58	BEL_BlBg_001_T3x_2	MZ901387	T-S	8652.34	100.00	16339

No.	Fasta name	Acc. no.	Source	Avg. fold	Covered percent	Length
59	BEL_BlBg_002_T3r_2	MZ901388	T-S	9029.42	100.00	16340
60	BGR_ShoR_001_T3o_2	MZ901722	T-S	5118.94	100.00	16339
61	BGR_ShoR_002_T3x_2	MZ901723	T-S	5995.63	100.00	16339
62	BGR_ShoR_003_T3r_2	MZ901724	T-S	5639.13	100.00	16340
63	BGR_ShoR_004_T3r_2	MZ901725	T-S	9019.13	100.00	16340
64	BGR_ShoR_005_T3x_2	MZ901726	T-S	8819.17	100.00	16340
65	BGR_ShoR_006_T3x_2	MZ901727	T-S	9684.67	100.00	16339
66	BGR_ShoR_007_T3x_2	MZ901728	T-S	8695.26	100.00	16338
67	BGR_ShoR_008_T3x_2	MZ901729	T-S	6454.49	100.00	16340
68	BIH_BuBo_001_T3x_2	MZ901389	T-S	4348.17	100.00	16338
69	BIH_BuBo_002_T3x_2	MZ901390	T-S	4864.58	100.00	16339
70	BIH_BuBo_003_T3p_2	MZ901391	T-S	3910.42	100.00	16341
71	BIH_BuBo_004_T3x_2	MZ901392	T-S	9260.90	100.00	16338
72	DEU_Brau_001_T3r_2	MZ901393	T-S	3908.76	100.00	16340
73	DEU_MuWe_001_T3p_2	MZ901711	T-S	2156.28	100.00	16338
74	DEU_MuWe_002_T3r_2	MZ901712	T-S	1393.08	100.00	16338
75	DEU_MuWe_003_T3q_2	MZ901713	T-S	550.52	100.00	16338
76	DEU_VieB_005_T3x_2	MZ901714	T-S	2640.95	100.00	16339
77	DEU_VieB_006_T3x_2	MZ901715	T-S	5133.50	100.00	16339
78	DEU_VieG_001_T3x_2	MZ901673	T-S	3936.79	100.00	16340
79	DEU_VieG_002_T3x_2	MZ901674	T-S	3890.51	100.00	16340
80	DEU_VieG_003_T3r_2	MZ901675	T-S	1546.50	100.00	16338
81	DEU_VieG_004_T3x_2	MZ901676	T-S	1932.71	100.00	16338
82	DEU_VieG_005_T3r_2	MZ901677	T-S	1985.71	100.00	16338
83	ESP_Saya_001_T3x_2	MZ901748	T-S	9385.01	100.00	16339
84	ESP_Saya_002_T1x_2	MZ901749	T-S	8897.22	100.00	16340
85	ESP_Saya_003_T3d_2	MZ901750	T-S	7765.74	100.00	16339
86	ESP_Saya_004_T3d_2	MZ901751	T-S	8443.13	100.00	16339
87	FRA_BlAq _001_T3x_2	MZ901381	T-S	4050.71	100.00	16340
88	FRA_BlAq_002_T3d_2	MZ901382	T-S	9610.14	100.00	16339

No.	Fasta name	Acc. no.	Source	Avg. fold	Covered percent	Length
89	FRA_BlAq_003_T3r_2	MZ901383	T-S	3558.39	100.00	16338
90	FRA_Tare_001_T3r_2	MZ901752	T-S	8000.83	100.00	16339
91	FRA_Tare_002_T3r_2	MZ901753	T-S	7983.01	100.00	16340
92	FRA_Tare_003_T3r_2	MZ901754	T-S	6780.42	100.00	16339
93	GBR_Gall_001_T3d_2	MZ901678	T-S	3908.50	100.00	16340
94	GBR_Gall_002_T3q_2	MZ901679	T-S	3344.76	100.00	16339
95	GRC_Cret_001_T3x_2	MZ901655	T-S	8468.92	100.00	16341
96	GRC_Cret_002_T3x_2	MZ901656	T-S	8278.95	100.00	16341
97	GRC_Kate_001_T3o_2	MZ901636	T-S	7332.38	100.00	16339
98	GRC_Kate_002_T3r_2	MZ901637	T-S	6424.47	100.00	16340
99	GRC_Kate_003_T3r_2	MZ901638	T-S	7675.93	100.00	16341
100	GRC_Kate_004_Q1x_2	MZ901639	T-S	8053.80	100.00	16339
101	GRC_Kate_005_T2x_2	MZ901640	T-S	3882.23	100.00	16339
102	GRC_Kate_006_T3x_2	MZ901641	T-S	3409.17	100.00	16340
103	GRC_Kate_007_T3x_2	MZ901642	T-S	4757.54	100.00	16340
104	GRC_KeaR_001_T2x_2	MZ901691	T-S	6621.74	100.00	16339
105	GRC_KeaR_002_T2x_2	MZ901692	T-S	7949.85	100.00	16339
106	GRC_KeaR_003_T2x_2	MZ901693	T-S	8183.09	100.00	16339
107	GRC_KeaR_004_T3x_2	MZ901694	T-S	8045.73	100.00	16340
108	GRC_Pres_005_T3x_2	MZ901643	T-S	5088.87	100.00	16339
109	GRC_Pres_006_T3o_2	MZ901644	T-S	6059.25	100.00	16338
110	GRC_Pres_007_T2x_2	MZ901645	T-S	3274.36	100.00	16339
111	GRC_Pres_008_T3h_2	MZ901646	T-S	3933.68	100.00	16339
112	GRC_ShoP_001_T3x_2	MZ901647	T-S	3639.03	100.00	16339
113	GRC_ShoP_002_Q1x_2	MZ901648	T-S	2994.75	100.00	16339
114	GRC_ShoP_003_T3x_2	MZ901649	T-S	3020.86	100.00	16339
115	GRC_ShoP_004_T3x_2	MZ901650	T-S	4408.80	100.00	16339
116	GRC_ShoR_001_T3q_2	MZ901651	T-S	4023.31	100.00	16340
117	GRC_ShoR_002_Q1x_2	MZ901652	T-S	4016.37	100.00	16339
118	GRC_ShoR_003_T3r_2	MZ901653	T-S	4280.99	100.00	16338

No.	Fasta name	Acc. no.	Source	Avg. fold	Covered percent	Length
119	GRC_ShoR_004_T1x_2	MZ901654	T-S	4462.54	100.00	16339
120	HRV_Bosk_001_T3c_2	MZ901608	T-S	8113.10	100.00	16339
121	HRV_Bosk_002_T3x_2	MZ901609	T-S	7743.85	100.00	16339
122	HRV_Bosk_003_T3r_2	MZ901610	T-S	7682.81	100.00	16340
123	HRV_Bosk_004_T3x_2	MZ901611	T-S	8218.18	100.00	16339
124	HRV_Bosk_005_T3x_2	MZ901612	T-S	1887.51	100.00	16338
125	HRV_Bosk_006_T3x_2	MZ901613	T-S	1729.85	100.00	16338
126	HRV_Bosk_007_T3x_2	MZ901614	T-S	1976.48	100.00	16338
127	HRV_Bosk_008_T3r_2	MZ901615	T-S	4.48	95.00	16338
128	HRV_Bosk_009_T3x_2	MZ901616	T-S	1364.92	100.00	16338
129	HRV_Bosk_010_T3r_2	MZ901617	T-S	394.78	100.00	16338
130	HRV_Bosk_011_T3x_2	MZ901618	T-S	1712.86	100.00	16338
131	HRV_Bosk_012_T3x_2	MZ901619	T-S	2264.83	100.00	16338
132	HRV_Bosk_013_T3x_2	MZ901580	T-S	2212.87	100.00	16338
133	HRV_Bosk_014_T3r_2	MZ901581	T-S	2058.05	100.00	16338
134	HRV_Bosk_015_T3x_2	MZ901582	T-S	2127.50	100.00	16338
135	HRV_Bosk_016_T3x_2	MZ901583	T-S	1894.14	100.00	16338
136	HRV_Bosk_017_T3x_2	MZ901584	T-S	1930.68	100.00	16338
137	HRV_Bosk_018_T3x_2	MZ901585	T-S	1443.99	100.00	16338
138	HRV_Bosk_019_T3c_2	MZ901586	T-S	1766.45	100.00	16338
139	HRV_Bosk_020_T3c_2	MZ901587	T-S	2221.79	100.00	16338
140	HRV_Bosk_021_T3r_2	MZ901588	T-S	1994.77	100.00	16338
141	HRV_Bosk_022_T3c_2	MZ901589	T-S	1926.35	100.00	16338
142	HRV_Bosk_023_T3o_2	MZ901590	T-S	2218.09	100.00	16338
143	HRV_Bosk_024_T3r_2	MZ901591	T-S	1934.88	100.00	16338
144	HRV_Bosk_025_T3q_2	MZ901592	T-S	1757.24	100.00	16338
145	HRV_Bosk_026_T3q_2	MZ901593	T-S	2034.28	100.00	16338
146	HRV_Bosk_027_T3j_2	MZ901594	T-S	1783.00	100.00	16338
147	HRV_Bosk_028_T1x_2	MZ901595	T-S	2223.01	100.00	16338
148	HRV_Bosk_029_T1x_2	MZ901596	T-S	2178.24	100.00	16338

No.	Fasta name	Acc. no.	Source	Avg. fold	Covered percent	Length
149	HRV_Bosk_030_T3x_2	MZ901597	T-S	2021.95	100.00	16338
150	HRV_Bosk_031_T3r_2	MZ901598	T-S	0.15	11.00	16338
151	HRV_Bosk_032_T3j_2	MZ901599	T-S	2111.22	100.00	16338
152	HRV_Bosk_033_T3p_2	MZ901600	T-S	1890.99	100.00	16338
153	HRV_Bosk_034_T3r_2	MZ901601	T-S	436.22	100.00	16338
154	HRV_Bosk_035_T3x_2	MZ901602	T-S	1981.40	100.00	16338
155	HRV_Bosk_036_T3q_2	MZ901603	T-S	2089.25	100.00	16338
156	HRV_Bosk_037_T3q_2	MZ901604	T-S	1973.20	100.00	16338
157	HRV_Bosk_038_T3x_2	MZ901605	T-S	2002.66	100.00	16338
158	HRV_Bosk_039_T3x_2	MZ901606	T-S	2214.43	100.00	16338
159	HRV_Bosk_040_T3c_2	MZ901607	T-S	2097.53	100.00	16338
160	HRV_BuHr_001_T1x_2	MZ901433	T-S	3855.46	100.00	16339
161	HRV_BuHr_002_T3r_2	MZ901434	T-S	4472.35	100.00	16339
162	HRV_BuHr_003_T3x_2	MZ901435	T-S	5628.22	100.00	16339
163	HRV_BuHr_004_T3r_2	MZ901436	T-S	5354.60	100.00	16340
164	HRV_BuHr_005_T3x_2	MZ901437	T-S	5018.45	100.00	16339
165	HRV_BuHr_006_T3x_2	MZ901438	T-S	4513.73	100.00	16339
166	HRV_BuHr_007_T3x_2	MZ901439	T-S	5903.81	100.00	16340
167	HRV_BuHr_008_T1a_2	MZ901461	T-S	2871.43	100.00	16341
168	HRV_BuHr_009_T1f_2	MZ901462	T-S	4256.32	100.00	16339
169	HRV_BuHr_010_T2x_2	MZ901463	T-S	3646.33	100.00	16339
170	HRV_BuHr_011_T3x_2	MZ901467	T-S	3570.79	100.00	16339
171	HRV_BuHr_012_T3r_2	MZ901468	T-S	3463.82	100.00	16339
172	HRV_BuHr_013_T3n_2	MZ901469	T-S	1135.95	100.00	16339
173	HRV_BuHr_014_T3x_2	MZ901431	T-S	1284.15	100.00	16338
174	HRV_BuHr_015_T2d_2	MZ901464	T-S	1823.66	100.00	16338
175	HRV_BuHr_016_T3x_2	MZ901432	T-S	2113.12	100.00	16338
176	HRV_BuHr_017_T3d_2	MZ901460	T-S	1883.53	100.00	16338
177	HRV_BuHr_018_T3r_2	MZ901465	T-S	1669.78	100.00	16338
178	HRV_BuHr_019_T3x_2	MZ901466	T-S	1890.69	100.00	16338

No.	Fasta name	Acc. no.	Source	Avg. fold	Covered percent	Length
179	HRV_BuHr_020_T3x_2	MZ901470	T-S	1781.58	100.00	16338
180	HRV_BuHr_021_T3x_2	MZ901440	T-S	1798.86	100.00	16338
181	HRV_BuHr_022_T2d_2	MZ901441	T-S	846.19	100.00	16338
182	HRV_BuHr_023_T3r_2	MZ901442	T-S	1973.82	100.00	16338
183	HRV_BuHr_024_T2a_2	MZ901443	T-S	1982.85	100.00	16338
184	HRV_BuHr_025_T3q_2	MZ901444	T-S	1838.98	100.00	16338
185	HRV_BuHr_026_T3p_2	MZ901445	T-S	1988.07	100.00	16338
186	HRV_BuHr_027_T3p_2	MZ901446	T-S	1783.32	100.00	16338
187	HRV_BuHr_028_T1a_2	MZ901447	T-S	1764.40	100.00	16338
188	HRV_BuHr_029_T3x_2	MZ901448	T-S	2034.39	100.00	16338
189	HRV_BuHr_030_T3q_2	MZ901449	T-S	1495.94	100.00	16338
190	HRV_BuHr_031_T3x_2	MZ901450	T-S	2069.20	100.00	16338
191	HRV_BuHr_032_T3r_2	MZ901451	T-S	2015.92	100.00	16338
192	HRV_BuHr_033_T3r_2	MZ901452	T-S	2306.98	100.00	16338
193	HRV_BuHr_034_T3r_2	MZ901453	T-S	2101.28	100.00	16338
194	HRV_BuHr_035_T3o_2	MZ901454	T-S	2105.83	100.00	16338
195	HRV_BuHr_036_T3r_2	MZ901455	T-S	1911.55	100.00	16338
196	HRV_BuHr_037_T3r_2	MZ901456	T-S	483.75	100.00	16338
197	HRV_BuHr_038_T3r_2	MZ901457	T-S	1251.61	100.00	16338
198	HRV_BuHr_039_T3x_2	MZ901458	T-S	1748.14	100.00	16338
199	HRV_BuHr_040_T3x_2	MZ901459	T-S	2087.92	100.00	16338
200	HRV_Podo_001_T3r_2	MZ901620	T-S	5741.53	100.00	16340
201	HRV_Podo_002_T3x_2	MZ901625	T-S	6103.71	100.00	16340
202	HRV_Podo_003_T3x_2	MZ901626	T-S	9500.66	100.00	16340
203	HRV_Podo_004_T3r_2	MZ901627	T-S	7481.67	100.00	16339
204	HRV_Podo_005_T3x_2	MZ901628	T-S	5246.69	100.00	16338
205	HRV_Podo_006_T3x_2	MZ901629	T-S	5993.97	100.00	16338
206	HRV_Podo_007_T3r_2	MZ901631	T-S	6207.61	100.00	16339
207	HRV_Podo_008_T3r_2	MZ901632	T-S	5469.06	100.00	16339
208	HRV_Podo_009_T3r_2	MZ901630	T-S	1742.03	100.00	16338

No.	Fasta name	Acc. no.	Source	Avg. fold	Covered percent	Length
209	HRV_Podo_010_T5b_2	MZ901633	T-S	1779.88	100.00	16338
210	HRV_Podo_011_T3r_2	MZ901634	T-S	2005.50	100.00	16338
211	HRV_Podo_012_T3r_2	MZ901635	T-S	1909.94	100.00	16338
212	HRV_Podo_013_T5b_2	MZ901621	T-S	1786.64	100.00	16338
213	HRV_Podo_014_T3x_2	MZ901622	T-S	1910.30	100.00	16338
214	HRV_Podo_015_T5b_2	MZ901623	T-S	1671.03	100.00	16338
215	HRV_Podo_016_T3r_2	MZ901624	T-S	1772.50	100.00	16338
216	KOS_BuDi_001_T2x_2	MZ901669	T-S	4516.09	100.00	16340
217	KOS_BuDi_002_T2x_2	MZ901670	T-S	3628.94	100.00	16340
218	KOS_BuDi_003_T2x_2	MZ901671	T-S	2824.57	100.00	16340
219	KOS_BuDi_004_T2x_2	MZ901672	T-S	3568.79	100.00	16340
220	KOS_BuRM_001_T3r_2	MZ901687	T-S	8513.20	100.00	16340
221	KOS_BuRM_002_T3x_2	MZ901688	T-S	5801.68	100.00	16339
222	KOS_BuRM_003_T3h_2	MZ901689	T-S	5733.54	100.00	16339
223	KOS_BuRM_004_T3b_2	MZ901690	T-S	6048.25	100.00	16339
224	KOS_BuSh_001_T3r_2	MZ901736	T-S	5100.51	100.00	16340
225	KOS_BuSh_002_T3x_2	MZ901737	T-S	6202.21	100.00	16340
226	KOS_BuSh_003_T3q_2	MZ901738	T-S	9653.39	100.00	16341
227	KOS_BuSh_004_T3s_2	MZ901739	T-S	3790.18	100.00	16339
228	MKD_BuMd_001_T1x_2	MZ901703	T-S	3014.81	100.00	16340
229	MKD_BuMd_002_T1x_2	MZ901704	T-S	3237.13	100.00	16340
230	MKD_BuMd_003_T3b_2	MZ901705	T-S	2074.53	100.00	16338
231	MKD_BuMd_004_T3x_2	MZ901706	T-S	1929.86	100.00	16340
232	MKD_BuMd_005_T3r_2	MZ901707	T-S	2161.11	100.00	16340
233	MNE_BuCG_001_T3x_2	MZ901708	T-S	2701.60	100.00	16340
234	MNE_BuCG_002_T3x_2	MZ901709	T-S	1718.44	100.00	16336
235	MNE_BuCG_003_T3x_2	MZ901710	T-S	1996.40	100.00	16339
236	MNG_Tura_001_T3n_2	MZ901756	T-S	8129.44	100.00	16338
237	MNG_Tura_002_T3b_2	MZ901757	T-S	8069.44	100.00	16338
238	MNG_Tura_003_T3d_2	MZ901759	T-S	7277.46	100.00	16340

No.	Fasta name	Acc. no.	Source	Avg. fold	Covered percent	Length
239	MNG_Tura_004_T2x_2	MZ901758	T-S	69955.01	100.00	16338
240	MNG_Tura_005_T3d_2	MZ901755	T-S	93492.24	100.00	16338
241	NLD_HoFr_017_T3x_2	MZ901471	T-S	13864.66	100.00	16338
242	NLD_HoFr_018_T3x_2	MZ901472	T-S	12953.90	100.00	16338
243	NLD_HoFr_019_T3r_2	MZ901473	T-S	12846.04	100.00	16338
244	NLD_HoFr_020_T3x_2	MZ901474	T-S	10442.13	100.00	16338
245	NLD_HoFr_021_T3x_2	MZ901475	T-S	12411.67	100.00	16338
246	NLD_HoFr_022_T3r_2	MZ901476	T-S	10103.29	100.00	16338
247	NLD_HoFr_023_T3x_2	MZ901481	T-S	11071.83	100.00	16338
248	NLD_HoFr_024_T3g_2	MZ901486	T-S	10678.61	100.00	16338
249	NLD_HoFr_025_T3r_2	MZ901492	T-S	9936.38	100.00	16338
250	NLD_HoFr_026_T3r_2	MZ901497	T-S	11678.28	100.00	16338
251	NLD_HoFr_027_T3x_2	MZ901504	T-S	10245.21	100.00	16338
252	NLD_HoFr_028_T3x_2	MZ901511	T-S	10227.32	100.00	16338
253	NLD_HoFr_029_T3h_2	MZ901516	T-S	10323.21	100.00	16338
254	NLD_HoFr_030_T3x_2	MZ901536	T-S	11500.39	100.00	16338
255	NLD_HoFr_031_T3x_2	MZ901537	T-S	9973.92	100.00	16338
256	NLD_HoFr_032_T3x_2	MZ901538	T-S	13730.10	100.00	16338
257	NLD_HoFr_033_T3x_2	MZ901539	T-S	10032.40	100.00	16338
258	NLD_HoFr_034_T3x_2	MZ901540	T-S	10804.91	100.00	16338
259	NLD_HoFr_035_T3x_2	MZ901541	T-S	10821.99	100.00	16338
260	NLD_HoFr_036_T3b_2	MZ901542	T-S	12298.56	100.00	16338
261	NLD_HoFr_037_T3b_2	MZ901543	T-S	10975.01	100.00	16338
262	NLD_HoFr_038_T3g_2	MZ901544	T-S	12741.20	100.00	16338
263	NLD_HoFr_039_T3r_2	MZ901545	T-S	11371.10	100.00	16338
264	NLD_HoFr_040_T3r_2	MZ901546	T-S	9317.97	100.00	16338
265	NLD_HoFr_041_T3x_2	MZ901547	T-S	10734.24	100.00	16338
266	NLD_HoFr_042_T3x_2	MZ901548	T-S	11366.49	100.00	16338
267	NLD_HoFr_043_T3x_2	MZ901549	T-S	10032.40	100.00	16338
268	NLD_HoFr_044_T3r_2	MZ901550	T-S	12834.61	100.00	16338

No.	Fasta name	Acc. no.	Source	Avg. fold	Covered percent	Length
269	NLD_HoFr_045_T3r_2	MZ901551	T-S	11680.85	100.00	16338
270	NLD_HoFr_046_T3x_2	MZ901552	T-S	7959.48	100.00	16338
271	NLD_HoFr_047_T3x_2	MZ901553	T-S	12900.13	100.00	16338
272	NLD_HoFr_048_T3x_2	MZ901554	T-S	13389.49	100.00	16338
273	NLD_HoFr_049_T3x_2	MZ901555	T-S	10320.40	100.00	16338
274	NLD_HoFr_050_T2x_2	MZ901556	T-S	9759.25	100.00	16338
275	NLD_HoFr_051_T3b_2	MZ901557	T-S	10881.08	100.00	16338
276	NLD_HoFr_052_T3h_2	MZ901558	T-S	10604.27	100.00	16338
277	NLD_HoFr_053_T3x_2	MZ901559	T-S	11300.99	100.00	16338
278	NLD_HoFr_054_T3o_2	MZ901560	T-S	11550.97	100.00	16338
279	NLD_HoFr_055_T3r_2	MZ901561	T-S	10658.86	100.00	16338
280	NLD_HoFr_056_T3r_2	MZ901562	T-S	10544.05	100.00	16338
281	NLD_HoFr_057_T3x_2	MZ901563	T-S	11010.80	100.00	16338
282	NLD_HoFr_058_T3d_2	MZ901564	T-S	11055.22	100.00	16338
283	NLD_HoFr_059_T3x_2	MZ901565	T-S	9638.94	100.00	16338
284	NLD_HoFr_060_T3x_2	MZ901566	T-S	10886.71	100.00	16338
285	NLD_HoFr_061_T3x_2	MZ901567	T-S	11321.02	100.00	16338
286	NLD_HoFr_062_T3x_2	MZ901568	T-S	11719.89	100.00	16338
287	NLD_HoFr_063_T1b_2	MZ901569	T-S	2279.21	100.00	16338
288	NLD_HoFr_064_T3r_2	MZ901570	T-S	2364.32	100.00	16338
289	NLD_HoFr_065_T3x_2	MZ901571	T-S	2666.28	100.00	16338
290	NLD_HoFr_066_T3n_2	MZ901572	T-S	2571.20	100.00	16338
291	NLD_HoFr_067_T3n_2	MZ901573	T-S	2540.62	100.00	16338
292	NLD_HoFr_068_T3r_2	MZ901574	T-S	2443.68	100.00	16338
293	NLD_HoFr_069_T3r_2	MZ901575	T-S	2664.05	100.00	16338
294	NLD_HoFr_070_T3p_2	MZ901576	T-S	2915.08	100.00	16338
295	NLD_HoFr_071_T3x_2	MZ901577	T-S	2572.84	100.00	16338
296	NLD_HoFr_072_T3x_2	MZ901578	T-S	2604.68	100.00	16338
297	NLD_HoFr_073_T3x_2	MZ901579	T-S	2956.36	100.00	16338
298	NLD_HoFr_074_T3r_2	MZ901477	T-S	3077.36	100.00	16338

No.	Fasta name	Acc. no.	Source	Avg. fold	Covered percent	Length
299	NLD_HoFr_075_T3x_2	MZ901478	T-S	2650.97	100.00	16338
300	NLD_HoFr_076_T3x_2	MZ901479	T-S	2915.49	100.00	16338
301	NLD_HoFr_077_T3x_2	MZ901480	T-S	2844.13	100.00	16338
302	NLD_HoFr_078_T3x_2	MZ901482	T-S	3338.42	100.00	16338
303	NLD_HoFr_079_T3b_2	MZ901483	T-S	2409.74	100.00	16338
304	NLD_HoFr_080_T3x_2	MZ901484	T-S	2774.81	100.00	16338
305	NLD_HoFr_081_T3x_2	MZ901485	T-S	2652.63	100.00	16338
306	NLD_HoFr_082_T3x_2	MZ901487	T-S	2838.93	100.00	16338
307	NLD_HoFr_083_T3r_2	MZ901488	T-S	2482.81	100.00	16338
308	NLD_HoFr_084_T3r_2	MZ901489	T-S	2679.33	100.00	16338
309	NLD_HoFr_085_T3r_2	MZ901490	T-S	1960.85	100.00	16338
310	NLD_HoFr_086_T3r_2	MZ901491	T-S	2209.09	100.00	16338
311	NLD_HoFr_087_T3g_2	MZ901493	T-S	2738.53	100.00	16338
312	NLD_HoFr_088_T3x_2	MZ901494	T-S	3006.14	100.00	16338
313	NLD_HoFr_089_T3r_2	MZ901495	T-S	2590.66	100.00	16338
314	NLD_HoFr_090_T3r_2	MZ901496	T-S	2942.28	100.00	16338
315	NLD_HoFr_091_T3b_2	MZ901498	T-S	2680.35	100.00	16338
316	NLD_HoFr_092_T3x_2	MZ901499	T-S	2785.24	100.00	16338
317	NLD_HoFr_093_T3x_2	MZ901500	T-S	2330.73	100.00	16338
318	NLD_HoFr_094_T3b_2	MZ901501	T-S	2140.34	100.00	16338
319	NLD_HoFr_095_T3r_2	MZ901502	T-S	3167.59	100.00	16338
320	NLD_HoFr_096_T3r_2	MZ901503	T-S	2735.09	100.00	16338
321	NLD_HoFr_097_T3x_2	MZ901505	T-S	2890.68	100.00	16338
322	NLD_HoFr_098_T3r_2	MZ901506	T-S	3290.60	100.00	16338
323	NLD_HoFr_099_T3g_2	MZ901507	T-S	3070.21	100.00	16338
324	NLD_HoFr_100_T3r_2	MZ901508	T-S	3345.46	100.00	16338
325	NLD_HoFr_101_T3r_2	MZ901509	T-S	2791.59	100.00	16338
326	NLD_HoFr_102_T3g_2	MZ901510	T-S	3029.04	100.00	16338
327	NLD_HoFr_103_T3r_2	MZ901512	T-S	2808.53	100.00	16338
328	NLD_HoFr_104_T3x_2	MZ901513	T-S	2836.41	100.00	16338

No.	Fasta name	Acc. no.	Source	Avg. fold	Covered percent	Length
329	NLD_HoFr_105_T3p_2	MZ901514	T-S	3224.85	100.00	16338
330	NLD_HoFr_106_T3x_2	MZ901515	T-S	2997.62	100.00	16338
331	NLD_HoFr_107_T3r_2	MZ901517	T-S	3642.57	100.00	16338
332	NLD_HoFr_108_T3x_2	MZ901518	T-S	2692.76	100.00	16338
333	NLD_HoFr_109_T2x_2	MZ901519	T-S	2856.25	100.00	16338
334	NLD_HoFr_110_T3r_2	MZ901520	T-S	2848.42	100.00	16338
335	NLD_HoFr_111_T3r_2	MZ901521	T-S	1592.40	100.00	16338
336	NLD_HoFr_112_T3x_2	MZ901522	T-S	1888.96	100.00	16338
337	NLD_HoFr_113_T3n_2	MZ901523	T-S	2154.79	100.00	16338
338	NLD_HoFr_114_T3r_2	MZ901524	T-S	1927.02	100.00	16338
339	NLD_HoFr_115_T3r_2	MZ901525	T-S	2035.24	100.00	16338
340	NLD_HoFr_116_T3r_2	MZ901526	T-S	1816.84	100.00	16338
341	NLD_HoFr_117_T3r_2	MZ901527	T-S	1542.62	100.00	16338
342	NLD_HoFr_118_T3r_2	MZ901528	T-S	1913.12	100.00	16338
343	NLD_HoFr_119_T3r_2	MZ901529	T-S	1771.41	100.00	16338
344	NLD_HoFr_120_T5x_2	MZ901530	T-S	1818.83	100.00	16338
345	NLD_HoFr_121_T3x_2	MZ901531	T-S	1679.47	100.00	16338
346	NLD_HoFr_122_T3x_2	MZ901532	T-S	1860.69	100.00	16338
347	NLD_HoFr_123_T3n_2	MZ901533	T-S	1698.03	100.00	16338
348	NLD_HoFr_124_T3x_2	MZ901534	T-S	1830.81	100.00	16338
349	NLD_HoFr_125_T3x_2	MZ901535	T-S	1659.20	100.00	16338
350	NLD_HoFr_126_T3b_2	MZ901720	T-S	95345.20	100.00	16338
351	NLD_HoFr_127_T3h_2	MZ901721	T-S	74335.76	100.00	16338
352	PRT_Barr_001_T3x_2	MZ901384	T-S	9278.25	100.00	16340
353	PRT_Barr_002_T1a_2	MZ901385	T-S	6353.02	100.00	16341
354	PRT_Barr_003_T1a_2	MZ901386	T-S	3404.01	100.00	16340
355	PRT_Maro_001_T3r_2	MZ901699	T-S	2821.17	100.00	16340
356	PRT_Maro_002_T3r_2	MZ901700	T-S	1951.65	100.00	16339
357	PRT_Maro_003_T3r_2	MZ901701	T-S	1198.87	100.00	16339
358	PRT_Maro_004_T3r_2	MZ901702	T-S	8031.92	100.00	16340

No.	Fasta name	Acc. no.	Source	Avg. fold	Covered percent	Length
359	RUS_Jaku_001_T3n_2	MZ901730	T-S	5715.34	100.00	16338
360	RUS_Jaku_002_T3r_2	MZ901733	T-S	9502.08	100.00	16339
361	RUS_Jaku_003_T2x_2	MZ901734	T-S	4645.14	100.00	16339
362	RUS_Jaku_004_T3k_2	MZ901735	T-S	5618.16	100.00	16339
363	RUS_Jaku_005_T3n_2	MZ901731	T-S	83889.22	100.00	16338
364	RUS_Jaku_006_T3r_2	MZ901732	T-S	94071.87	100.00	16338
365	SCO_Here_001_T3x_2	MZ901680	T-S	2851.69	100.00	16339
366	SCO_Here_002_T3r_2	MZ901681	T-S	3269.88	100.00	16340
367	SCO_Here_003_T3x_2	MZ901682	T-S	3804.07	100.00	16339
368	SCO_ScHi_001_T3g_2	MZ901683	T-S	3033.04	100.00	16339
369	SCO_ScHi_002_T3r_2	MZ901684	T-S	2918.01	100.00	16340
370	SRB_BuSr_001_T3x_2	MZ901744	T-S	8390.06	100.00	16339
371	SRB_BuSr_002_T3r_2	MZ901745	T-S	8793.01	100.00	16339
372	SRB_BuSr_003_T3x_2	MZ901746	T-S	9249.52	100.00	16340
373	SRB_BuSr_004_T3x_2	MZ901747	T-S	9442.71	100.00	16339
374	SVN_Cika_001_T3r_2	MZ901657	T-S	200.45	100.00	16339
375	SVN_Cika_002_T3x_2	MZ901658	T-S	2890.63	100.00	16339
376	SVN_Cika_003_T3r_2	MZ901659	T-S	2265.00	100.00	16340
377	SVN_Cika_004_T3x_2	MZ901660	T-S	3598.30	100.00	16340
378	SVN_Cika_005_T3r_2	MZ901661	T-S	3060.29	100.00	16340
379	SVN_Cika_006_T3r_2	MZ901662	T-S	4332.14	100.00	16340
380	SVN_Cika_007_T3x_2	MZ901663	T-S	3828.97	100.00	16339
381	SVN_Cika_008_T3x_2	MZ901664	T-S	4719.01	100.00	16339
382	TUR_AnaB_001_T3r_2	MZ901378	T-S	3882.00	100.00	16340
383	TUR_AnaB_002_T3r_2	MZ901375	T-S	3057.46	100.00	16338
384	TUR_AnaB_003_T3x_2	MZ901376	T-S	3536.82	100.00	16339
385	TUR_AnaB_004_T2x_2	MZ901377	T-S	3224.66	100.00	16339
695	ETH_Bora_001_T1b_3	SRS1346805	rWGS	463.40	100.00	16338
696	ETH_Bora_002_T1b_3	SRS1429491	rWGS	463.40	100.00	16338
697	ETH_Bora_003_T1x_3	SRS1727978	rWGS	565.51	100.00	16338

No.	Fasta name	Acc. no.	Source	Avg. fold	Covered percent	Length
698	ETH_Bora_004_T1x_3	SRS1429391	rWGS	422.16	100.00	16338
699	ETH_Bora_005_T1b_3	SRS1437860	rWGS	269.28	100.00	16338
700	ETH_Ogad_001_T1b_3	SRS1512520	rWGS	470.41	100.00	16338
701	ETH_Ogad_002_T1b_3	SRS1512522	rWGS	427.98	100.00	16338
702	ETH_Ogad_003_T1x_3	SRS1512525	rWGS	674.94	100.00	16338
703	ETH_Ogad_004_T1b_3	SRS1512526	rWGS	645.29	100.00	16338
704	SDN_Kena_001_T1x_3	SRS1439369	rWGS	37.34	99.99	16338
705	SDN_Kena_002_T1x_3	SRS1446916	rWGS	118.22	99.99	16338
706	SDN_Kena_003_T1b_3	SRS1437861	rWGS	63.51	99.99	16338
707	SDN_Kena_004_T1b_3	SRS1439366	rWGS	53.72	99.99	16338
708	SDN_Kena_005_T1b_3	SRS1439367	rWGS	187.00	99.99	16338
709	SDN_Kena_006_T1x_3	SRS1439368	rWGS	162.08	100.00	16338
710	SDN_Kena_007_T1b_3	SRS1439001	rWGS	111.36	99.99	16338
711	SDN_Kena_008_T1c_3	SRS1439005	rWGS	111.44	100.00	16338
712	SEN_Ndam_001_T1x_3	SRS1512498	rWGS	498.11	100.00	16338
713	SEN_Ndam_002_T1x_3	SRS1512514	rWGS	382.13	100.00	16338
714	UGA_Anko_001_T1b_3	SRS1338704	rWGS	311.65	100.00	16338
715	UGA_Anko_002_T1b_3	SRS1496254	rWGS	357.68	100.00	16338
716	UGA_Anko_003_T1c_3	SRS1496257	rWGS	406.40	100.00	16338
717	UGA_Anko_004_T1b_3	SRS1496259	rWGS	435.53	100.00	16338
718	UGA_Anko_005_T1b_3	SRS1496260	rWGS	327.02	100.00	16338
719	UGA_Anko_006_T1b_3	SRS1496261	rWGS	429.07	100.00	16338
720	UGA_Anko_007_T1x_3	SRS1496262	rWGS	422.83	100.00	16338
721	UGA_Anko_008_T1x_3	SRS1496265	rWGS	381.03	100.00	16338
722	DEU_VieG_001_T3x_3	SRS628363	rWGS	1451.46	100.00	16338
723	DEU_VieG_002_T3x_3	SRS629675	rWGS	1542.75	100.00	16338
724	DEU_VieG_003_T3r_3	SRS631222	rWGS	1541.70	100.00	16338
725	DEU_VieG_004_T3r_3	SRS629679	rWGS	1780.09	100.00	16338
726	DEU_VieG_005_T3x_3	SRS629674	rWGS	1569.40	100.00	16338
727	DEU_VieG_006_T3x_3	SRS629676	rWGS	1912.28	100.00	16338

No.	Fasta name	Acc. no.	Source	Avg. fold	Covered percent	Length
728	DEU_VieG_007_T3p_3	SRS631221	rWGS	1983.74	100.00	16338
729	FRA_Limo_002_T3r_3	SRS378721	rWGS	32.99	99.99	16338
730	GBR_AngR_001_T3r_3	SRS629341	rWGS	430.79	100.00	16338
731	GBR_Angu_008_T3x_3	SRS629677	rWGS	872.69	100.00	16338
732	GBR_Angu_009_T3s_3	SRS631217	rWGS	932.91	100.00	16338
733	GBR_Angu_010_T3r_3	SRS428688	rWGS	293.08	100.00	16338
734	GBR_Angu_011_T3r_3	SRS641738	rWGS	1180.65	100.00	16338
735	GBR_Angu_012_T3r_3	SRS641740	rWGS	1059.89	100.00	16338
736	GBR_Angu_013_T3x_3	SRS629088	rWGS	79.17	99.99	16338
737	GBR_Angu_014_T1x_3	SRS629091	rWGS	324.88	99.99	16338
738	GBR_Angu_018_T3n_3	SRS631214	rWGS	947.28	100.00	16338
739	GBR_Angu_019_T3d_3	SRS428686	rWGS	350.53	100.00	16338
740	GBR_Angu_020_T3r_3	SRS641739	rWGS	1864.83	100.00	16338
741	GBR_Angu_021_T3r_3	SRS629330	rWGS	681.55	100.00	16338
742	GBR_Angu_022_T6x_3	SRS629688	rWGS	598.58	100.00	16338
743	GBR_Angu_023_T3n_3	SRS629690	rWGS	687.02	100.00	16338
744	GBR_Here_003_T3x_3	SRS631191	rWGS	583.29	100.00	16338
745	GBR_Here_004_T3x_3	SRS631218	rWGS	69.46	99.99	16338
746	NLD_HoFr_004_T3x_3	SRS629342	rWGS	1270.45	100.00	16338
747	CHE_Simm_008_T3r_4	T-S	rWGS	1.18	60.11	16338
748	CHE_Simm_006_T3x_3	T-S	rWGS	35.71	100.00	16338
749	CHE_Simm_007_T3i_3	T-S	rWGS	27.68	100.00	16338
750	CHE_Simm_001_T3r_4	SRS666732	rWGS	6.16	98.21	16338
751	CHE_Simm_002_T3r_4	SRS666781	rWGS	11.31	99.93	16338
752	CHE_Simm_003_T3x_4	SRS666789	rWGS	7.29	98.55	16338
753	CHE_Simm_004_T3n_4	SRS666784	rWGS	7.14	98.38	16338
754	CHE_Simm_005_T3x_4	SRS666776	rWGS	4.08	84.80	16338
755	DEU_VieG_008_T3x_4	SRS628368	rWGS	8.25	99.89	16338
756	DEU_VieG_009_T3r_4	SRS629684	rWGS	11.10	99.53	16338
757	DEU_VieG_010_T3x_4	SRS629693	rWGS	11.62	99.81	16338

No.	Fasta name	Acc. no.	Source	Avg. fold	Covered percent	Length
758	DEU_VieG_011_T3r_4	SRS628364	rWGS	7.11	99.80	16338
759	DEU_VieG_012_T3r_4	SRS628367	rWGS	7.46	99.55	16338
760	FRA_BlAq_001_T3s_4	SRS428663	rWGS	11.24	90.98	16338
761	FRA_Char_001_T3r_4	SRS629324	rWGS	2.88	91.61	16338
762	FRA_Char_002_T3r_4	SRS629327	rWGS	15.59	99.36	16338
763	FRA_Char_003_T3x_4	SRS428678	rWGS	5.79	98.43	16338
764	FRA_Char_004_T3n_4	SRS628370	rWGS	1025.93	100.00	16338
765	FRA_Char_005_T3r_4	SRS628376	rWGS	6.52	98.07	16338
766	FRA_Char_006_T3r_4	SRS631207	rWGS	7.60	99.38	16338
767	FRA_Char_007_T3r_4	SRS666720	rWGS	7.49	97.63	16338
768	FRA_Char_008_T3r_4	SRS666710	rWGS	3.44	92.76	16338
769	FRA_Char_009_T3r_4	SRS628372	rWGS	11.14	99.56	16338
770	FRA_Limo_001_T3r_4	SRS631220	rWGS	1.92	83.26	16338
771	FRA_Limo_003_T3r_4	SRS631219	rWGS	7.21	98.87	16338
772	FRA_Limo_004_T3r_4	SRS428694	rWGS	3.65	90.05	16338
773	FRA_MAnj_001_T3p_4	SRS631210	rWGS	5.62	89.96	16338
774	FRA_MAnj_002_T3r_4	SRS629692	rWGS	3.86	94.41	16338
775	FRA_MAnj_003_T3r_4	SRS631215	rWGS	11.93	99.83	16338
776	FRA_MAnj_004_T3p_4	SRS629671	rWGS	2.55	87.26	16338
777	GBR_AngR_002_T3x_4	SRS641741	rWGS	7.44	98.99	16338
778	GBR_AngR_003_T3r_4	SRS666722	rWGS	1.65	76.75	16338
779	GBR_AngR_004_T3r_4	SRS666690	rWGS	2.44	90.77	16338
780	GBR_Angu_001_T3r_4	SRS428689	rWGS	2.44	84.09	16338
781	GBR_Angu_002_T3r_4	SRS428684	rWGS	11.96	97.71	16338
782	GBR_Angu_003_T3r_4	SRS428690	rWGS	10.79	97.96	16338
783	GBR_Angu_004_T3r_4	SRS629322	rWGS	4.65	95.12	16338
784	GBR_Angu_005_T3r_4	SRS641715	rWGS	8.02	99.19	16338
785	GBR_Angu_006_T3n_4	SRS428675	rWGS	2.44	81.48	16338
786	GBR_Angu_007_T3r_4	SRS641713	rWGS	5.31	94.22	16338
787	GBR_Angu_015_T3r_4	SRS428687	rWGS	1.87	76.33	16338

No.	Fasta name	Acc. no.	Source	Avg. fold	Covered percent	Length
788	GBR_Angu_016_T3r_4	SRS428672	rWGS	1.34	56.13	16338
789	GBR_Angu_017_T3c_4	SRS628375	rWGS	551.39	100.00	16338
790	GBR_Angu_024_T3r_4	SRS428691	rWGS	2.10	82.79	16338
791	GBR_Angu_025_T3q_4	SRS666693	rWGS	4.30	96.40	16338
792	GBR_Angu_026_T3r_4	SRS666773	rWGS	4.51	97.72	16338
793	GBR_Angu_027_T3r_4	SRS666774	rWGS	4.67	85.54	16338
794	GBR_Angu_028_T3r_4	SRS666771	rWGS	2.44	89.29	16338
795	GBR_Angu_029_T3r_4	SRS666769	rWGS	1.25	65.17	16338
796	GBR_Here_001_T3x_4	SRS631195	rWGS	7.64	99.90	16338
797	GBR_Here_002_T3n_4	SRS629694	rWGS	9.17	99.82	16338
798	ITA_Pied_001_T3r_4	SRS428665	rWGS	13.82	99.98	16338
799	ITA_Pied_002_T3x_4	SRS666704	rWGS	8.80	99.92	16338
800	ITA_Pied_003_T1c_4	SRS666712	rWGS	2.68	91.20	16338
801	ITA_Pied_004_T3x_4	SRS666707	rWGS	1.69	72.42	16338
802	ITA_Pied_005_T3r_4	SRS666711	rWGS	8.40	99.29	16338
803	ITA_Pied_006_T3x_4	SRS666714	rWGS	2.80	91.30	16338
804	NLD_HoFr_001_T3r_4	SRS641735	rWGS	8.20	99.05	16338
805	NLD_HoFr_002_T3x_4	SRS629104	rWGS	8.30	99.84	16338
806	NLD_HoFr_003_T3x_4	SRS641722	rWGS	5.86	94.49	16338
807	NLD_HoFr_005_T3r_4	SRS641729	rWGS	9.69	96.88	16338
808	NLD_HoFr_006_T3r_4	SRS631189	rWGS	3.05	64.49	16338
809	NLD_HoFr_007_T3r_4	SRS631202	rWGS	4.06	72.79	16338
810	NLD_HoFr_008_T3r_4	SRS641744	rWGS	15.82	99.98	16338

Table S6. Nucleotide substitution rate matrix.

	**A**	**C**	**G**	**T**
**A**	*	0.04404	1	0.04404
**C**	-	*	0.04404	1.14344
**G**	-	-	*	0.01892
**T**	-	-	-	*

Table S7. Table presents results of model test for the selected dataset.

Substitution model	Time clock	Tree Prior	AIC	Likelihood				
				mean	stderr	stdev	variance	ESS
GTR	Strict[1]	Skyline[4]	61933.16	-30933	0.05	5.72	32.81	13069
GTR	Strict[1]	Exponential[5]	61943.04	-30935	0.04	6.02	36.28	18355
GTR	Strict[1]	Constant[6]	61946.32	-30935	0.16	6.15	37.88	1480
TN83	Strict[1]	Skyline[4]	61948.16	-30943	0.05	5.56	30.94	10286
GTR	Log[2]	Skyline[4]	61955.49	-30924	0.07	7.27	52.98	10234
HKY	Strict[1]	Skyline[4]	61957.29	-30948	0.04	5.49	30.16	13493
GTR	Log[2]	Exponential[5]	61963.74	-30927	0.07	7.34	54.02	9035
GTR	Log[2]	Constant[6]	62006.70	-30928	0.26	8.67	75.19	1092
JC69	Strict[1]	Skyline[4]	66089.23	-33019	0.04	4.96	24.67	13961
GTR	Exp[3]	Exponential[5]	134911.30	-67384	0.07	8.41	70.76	11486
TN83	Exp[3]	Skyline[4]	134925.19	-67421	0.04	6.40	40.96	24185
GTR	Exp[3]	Skyline[4]	135116.76	-67497	0.07	7.80	60.92	9758
GTR	Exp[3]	Constant[6]	135163.72	-67508	0.07	8.58	73.73	13831
HKY	Exp[3]	Skyline[4]	188066.50	-93947	0.80	9.26	85.76	131
JC69	Exp[3]	Skyline[4]	332552.20	-166238	0.07	6.10	37.26	7384

Abbreviation:[1] Strict molecular clock;[2] Uncorrelated relaxed lognormal molecular clock;[3] Uncorrelated relaxed exponential molecular clock;[4] Coalescent bayesian skyline;[5] Coalescent exponential size;[6] Coalescent constant size.

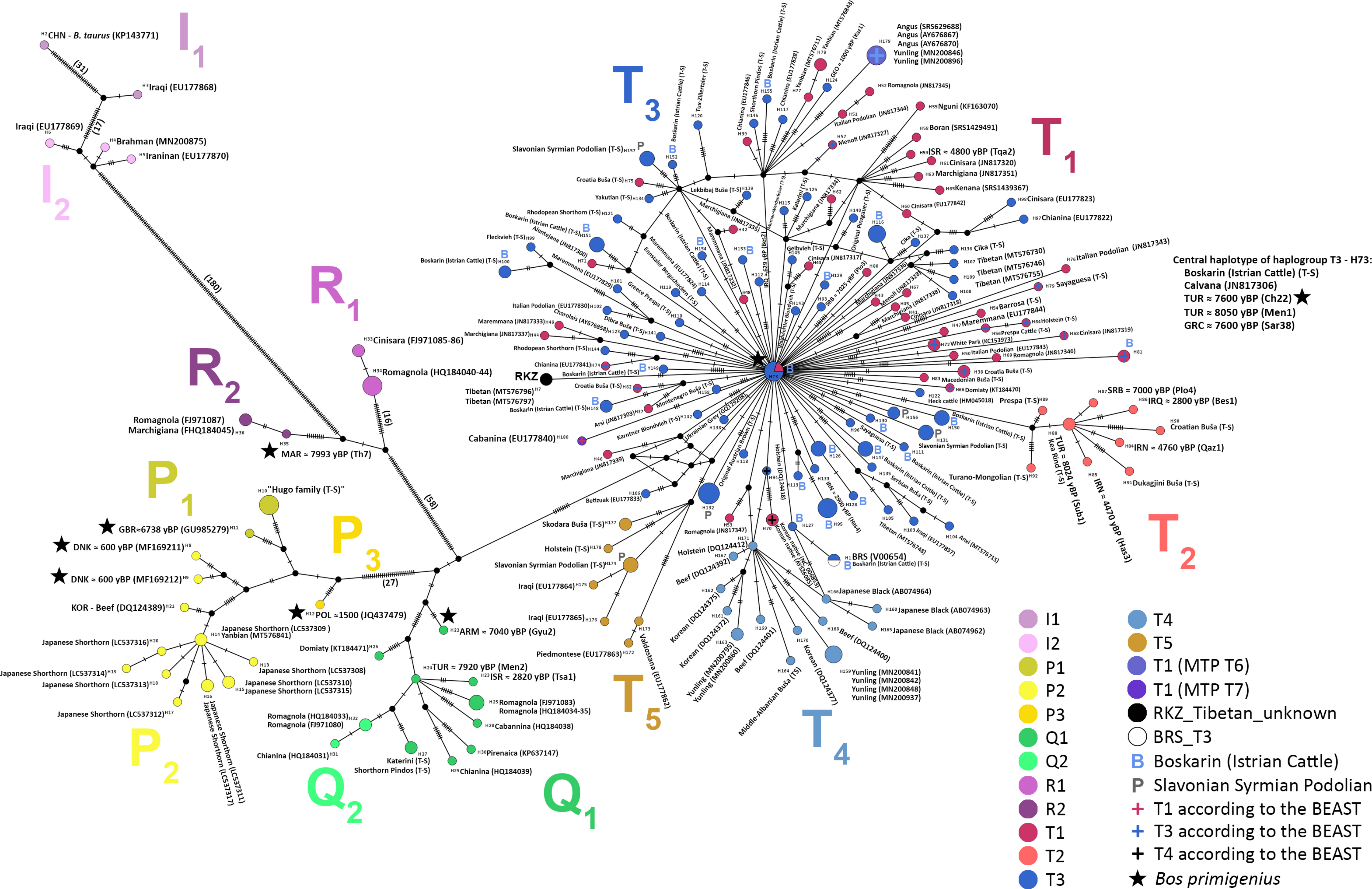

I1
I2
R1
R2
P1
P2
P3
Q1
Q2
T1
T2
T3
T4
T5
H2 CHN - B. taurus (KP143771)
(31)
H3 Iraqi (EU177868)
Iraqi (EU177869) H6
(17)
H4 Brahman (MN200875)
H5 Iraninan (EU177870)
(180)
H33 Cinisara (FJ971085-86)
H34 Romagnola (HQ184040-44)
(16)
Romagnola (FJ971087)
Marchigiana (FHQ184045) H36
H35
MAR ≈ 7993 yBP (Th7)
(58)
H10 "Hugo family (T-S)"
GBR≈6738 yBP (GU985279) H11
DNK ≈ 600 yBP (MF169211) H8
DNK ≈ 600 yBP (MF169212) H9
KOR - Beef (DQ124389) H21
(27)
H12 POL ≈1500 (JQ437479)
H22 ARM ≈ 7040 yBP (Gyu2)
Japanese Shorthorn (LC537309)
H14 Yanbian (MT576841)
Japanese Shorthorn (LC537316) H20
Japanese Shorthorn (LC537314) H19
Japanese Shorthorn (LC537313) H18
Japanese Shorthorn (LC537312) H17
H13 Japanese Shorthorn (LC537308)
Japanese Shorthorn (LC537310)
H15 Japanese Shorthorn (LC537315)
H16
Japanese Shorthorn (LC537311)
Japanese Shorthorn (LC537317)
Domiaty (KT184471) H26
H24 TUR ≈ 7920 yBP (Men2)
H23 ISR ≈ 2820 yBP (Tsa1)
H25 Romagnola (FJ971083)
Romagnola (HQ184034-35)
Romagnola (HQ184033) H32
Romagnola (FJ971080)
H28 Cabannina (HQ184038)
Chianina (HQ184031) H31
H27
Katerini (T-S)
Shorthorn Pindos (T-S)
H30 Pirenaica (KP637147)
H29 Chianina (HQ184039)
RKZ
Tibetan (MT576796) H7
Tibetan (MT576797)
Cabanina (EU177840) H180
Skodara Buša (T-S) H177
Holstein (T-S) H178
Slavonian Syrmian Podolian (T-S) H174
Iraqi (EU177864) H175
Iraqi (EU177865) H176
Piedmontese (EU177863) H172
H173
Valdostana (EU177862)
Slavonian Syrmian Podolian (T-S) H157
Croatia Buša (T-S) H75
Yakutian (T-S) H134
Rhodopean Shorthorn (T-S) H121
Fleckvieh (T-S) H99
Boskarin (Istrian Cattle) (T-S) H100
Italian Podolian (EU177830) H102
Maremmana (JN817333) H49
Marchigiana (JN817337) H44
Rhodopean Shorthorn (T-S) H144
Chianina (EU177841) H74
Boskarin (Istrian Cattle) (T-S)
Croatia Buša (T-S) H82
Boskarin (Istrian Cattle) (T-S) H148
Arsi (JN817303) H37
Karntner Blondvieh (T-S) H142
Ukrainian Grey (GQ129208)
Marchigiana (JN817339)
Betizuak (EU177833)
Romagnola (JN817347)
Holstein (DQ124412)
Beef (DQ124392)
Korean (DQ124375)
Korean (DQ124372)
Yunling (MN200795)
Yunling (MN200860)
Beef (DQ124401)
Middle-Albanian Buša (T-S)
Korean (DQ124377)
H159 Yunling (MN200841)
Yunling (MN200842)
Yunling (MN200848)
Yunling (MN200937)
H168 Beef (DQ124400)
H165 Japanese Black (AB074962)
H160 Japanese Black (AB074963)
H166 Japanese Black (AB074964)
H1 BRS (V00654)
Boskarin (Istrian Cattle) (T-S)
Angus (SRS629688)
Angus (AY676867)
Angus (AY676870)
Yunling (MN200846)
Yunling (MN200896)
H52 Romagnola (JN817345)
H55 Nguni (KF163070)
H58 Boran (SRS1429491)
H59 ISR ≈ 4800 yBP (Tqa2)
H61 Cinisara (JN817320)
H63 Marchigiana (JN817351)
H65 Kenana (SRS1439367)
H60 Cinisara (EU177842)
H98 Cinisara (EU177823)
H97 Chianina (EU177822)
H136 Cika (T-S)
H107 Tibetan (MT576730)
H109 Tibetan (MT576746)
Tibetan (MT576755)
H76 Italian Podolian (JN817343)
H79 Sayaguesa (T-S)
H54 Barrosa (T-S)
H47 Maremmana (EU177844)
H64 Holstein (T-S)
H56 Prespa Cattle (T-S)
H68 Cinisara (JN817319)
H72 White Park (KC153973)
H50 Italian Podolian (EU177843)
H69 Romagnola (JN817346)
H81
H38 Croatia Buša (T-S)
H83 Macedonian Buša (T-S)
H66 Domiaty (KT184470)
H122 Heck cattle (HM045018)
Prespa (T-S) H89
H87 SRB ≈ 7000 yBP (Plo4)
H86 IRQ ≈ 2800 yBP (Bes1)
H90 Croatian Buša (T-S)
H84 IRN ≈ 4760 yBP (Qaz1)
H91 Dukagjini Buša (T-S)
H88 TUR ≈ 8024 yBP (Sub1)
Kea Rind (T-S)
H85 IRN ≈ 4470 yBP (Has3)
Slavonian Syrmian Podolian (T-S)
Turano-Mongolian (T-S) H92
Central haplotype of haplogroup T3 - H73:
Boskarin (Istrian Cattle) (T-S)
Calvana (JN817306)
TUR ≈ 7600 yBP (Ch22)
TUR ≈ 8050 yBP (Men1)
GRC ≈ 7600 yBP (Sar38)
I1
I2
P1
P2
P3
Q1
Q2
R1
R2
T1
T2
T3
T4
T5
T1 (MTP T6)
T1 (MTP T7)
RKZ_Tibetan_unknown
BRS_T3
B Boskarin (Istrian Cattle)
P Slavonian Syrmian Podolian
+ T1 according to the BEAST
+ T3 according to the BEAST
+ T4 according to the BEAST
★ Bos primigenius

Figure S1. The Median-Joining network for the 16210-bp dataset showing the phylogenetic positions of 180 haplotypes found in 247 mitogenome sequences of the species *Bos taurus*, *Bos primigenius*, and *Bos indicus*.

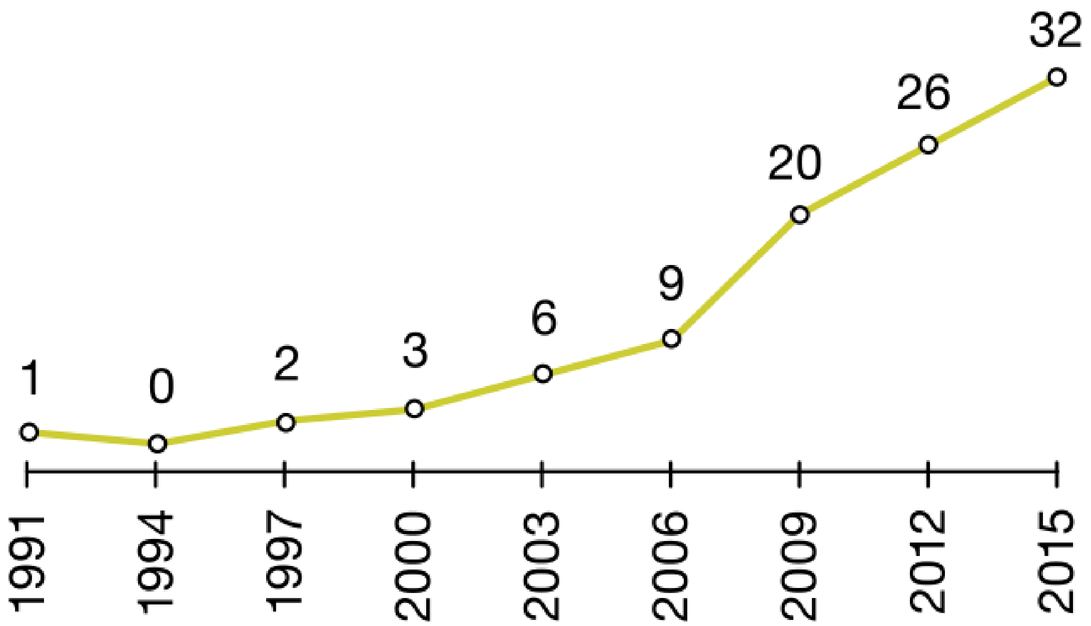

Figure S2. Trend presenting number of animals born in maternal lineage with P haplotype mitochondria.

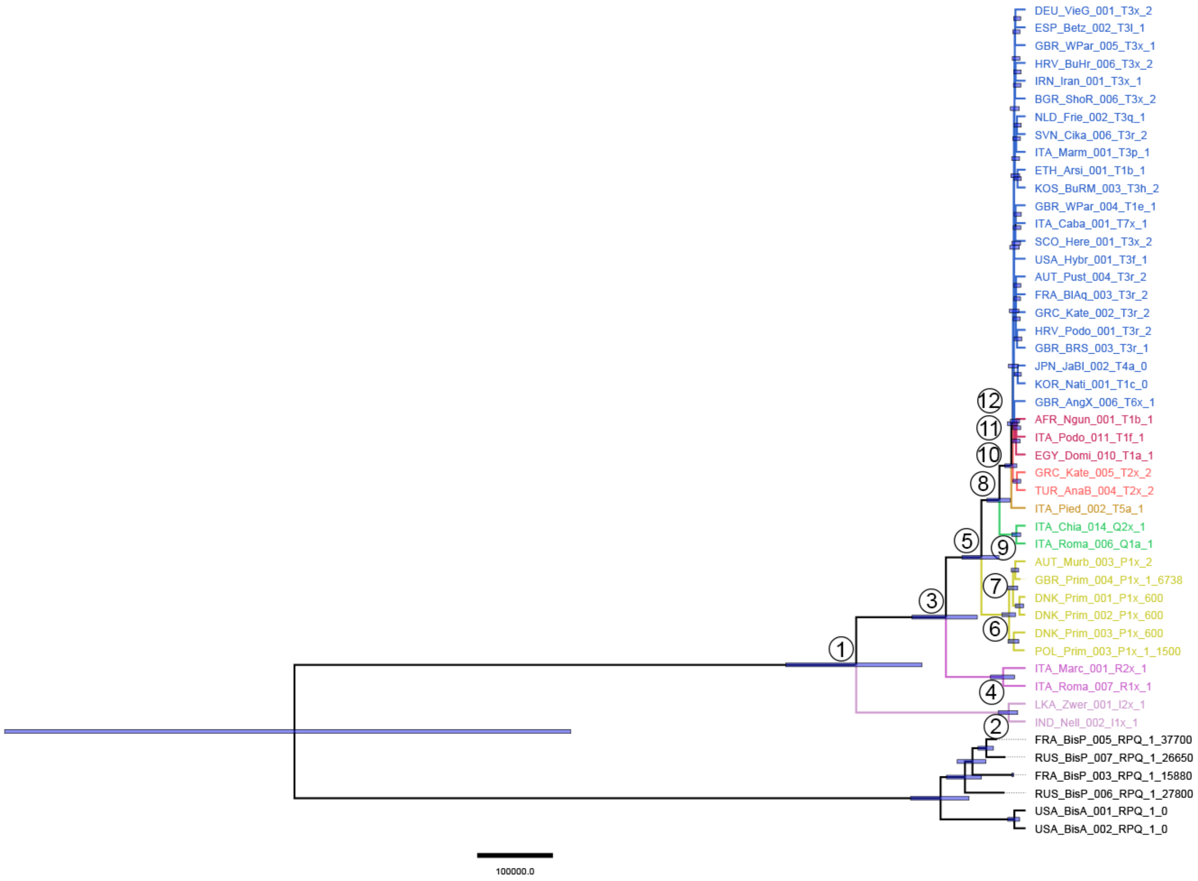

Figure S3. The calibration MCA tree as result of model testing for *Bos* clades using tip dates. The color of the lines represents haplotypes according to the results and the classification of MitoToolPy, color coordinates are given in RGB, black is outlawyer group *Bison americanus* and *Bison Priscus*. Haplogroup time divergence given in Table 1.

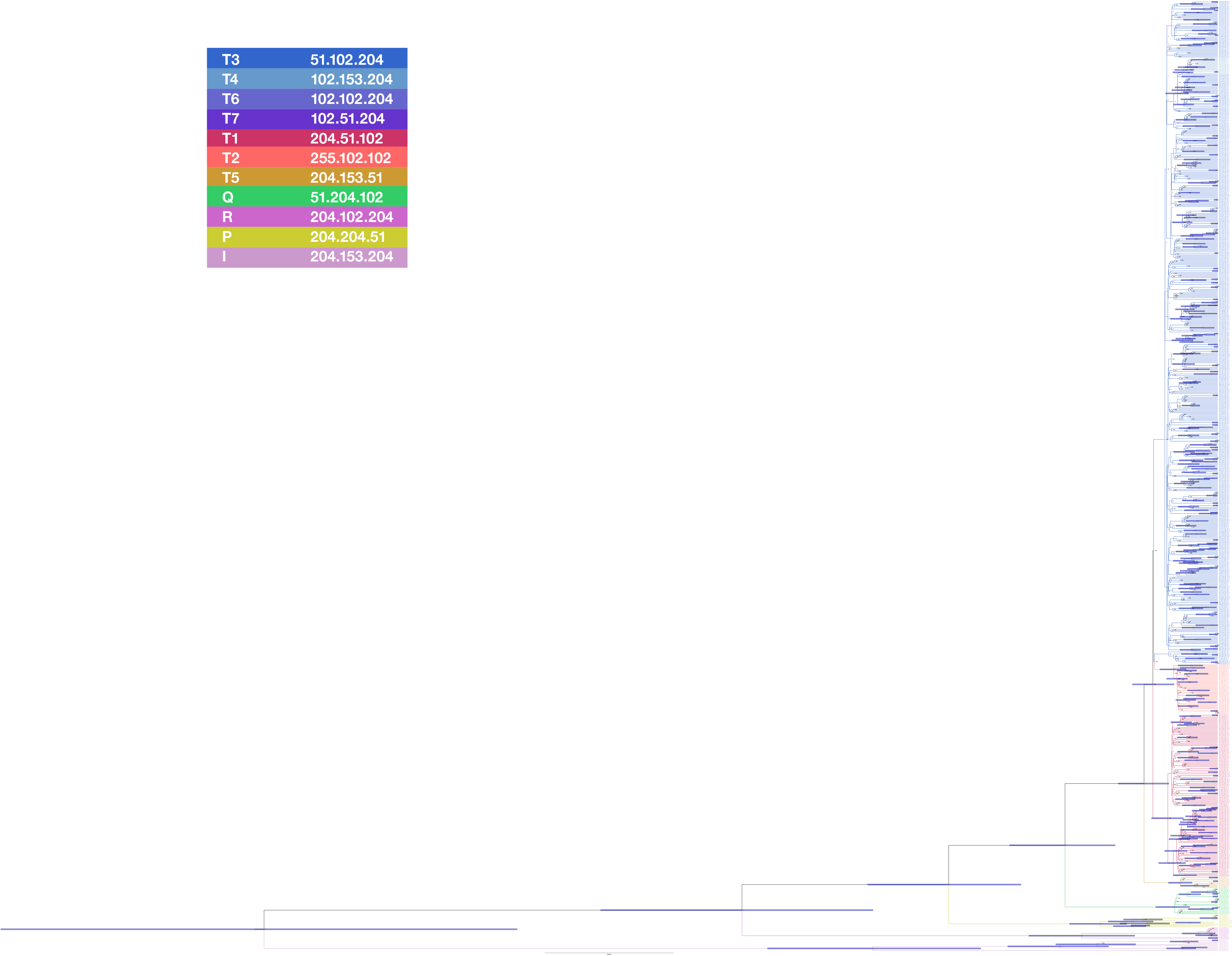

T3 51.102.204
T4 102.153.204
T6 102.102.204
T7 102.51.204
T1 204.51.102
T2 255.102.102
T5 204.153.51
Q 51.204.102
R 204.102.204
P 204.204.51
I 204.153.204

Figure S4. The complete genome MCA tree. Node numbers present Posterior probability for each node. Blue bars' position on nodes presents 95%HPD value. The color of lines presents haplotypes according to the results and the classification of MitoToolPy, color coordinates are given in RGB.

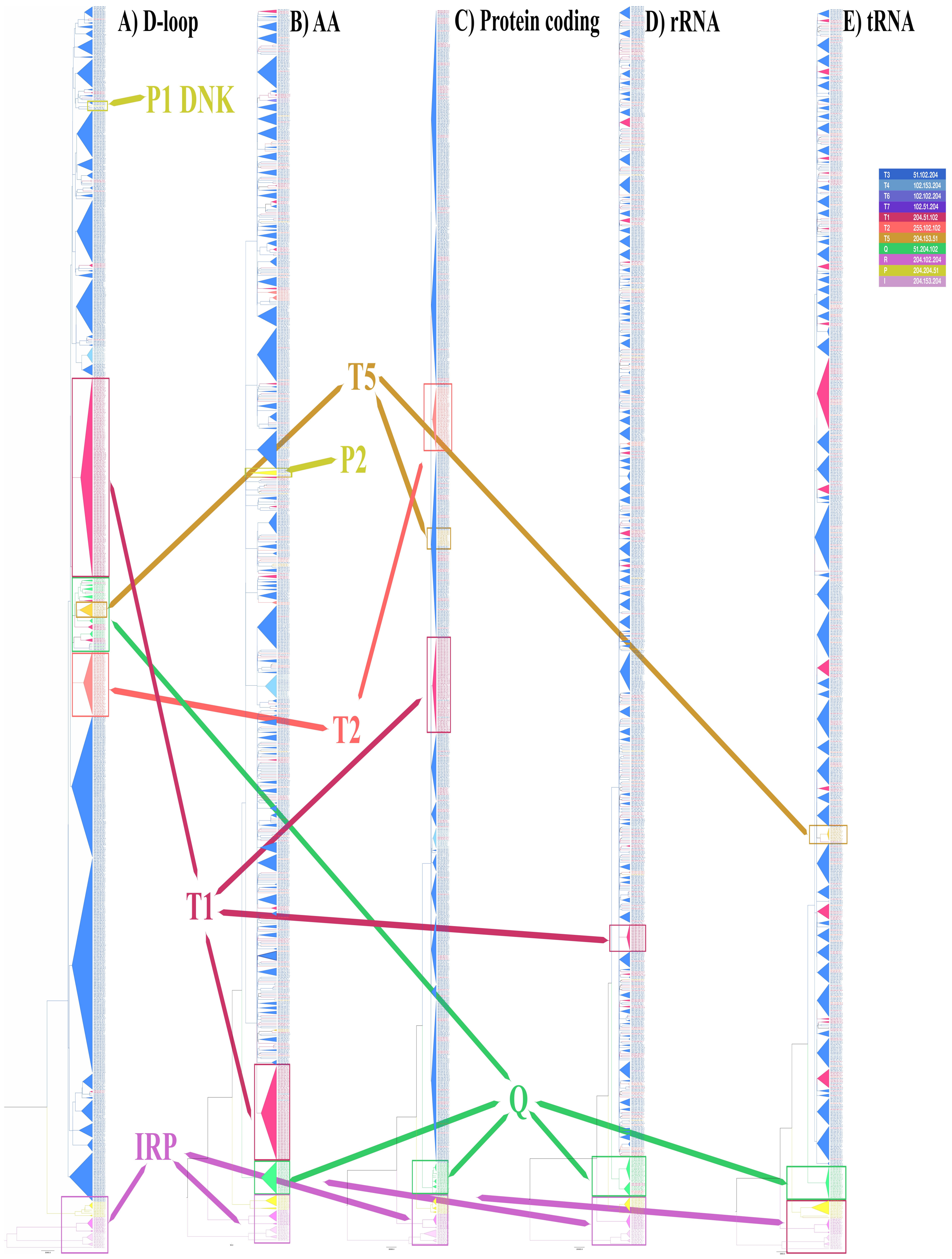

A) D-loop
B) AA
C) Protein coding
D) rRNA
E) tRNA
P1 DNK
P2
T5
T2
T1
Q
IRP
T3 51.102.204
T4 102.153.204
T6 102.102.204
T7 102.51.204
T1 204.51.102
T2 255.102.102
T5 204.153.51
Q 51.204.102
R 204.102.204
P 204.204.51
I 204.153.204

Figure S5. MCA trees calculated using 4 functionally different regions of mtDNA. A. tree calculated using just the D-loop region; B. tree calculated using amino acid alignment of the merged 13 regions that encodes protein; C. tree calculated using nucleotide alignment of the merged 13 regions that encodes protein; D. tree calculated using just two genes 12S and 16S that encode ribosomal RNA; E. tree calculated using genes that encode transport RNAs.

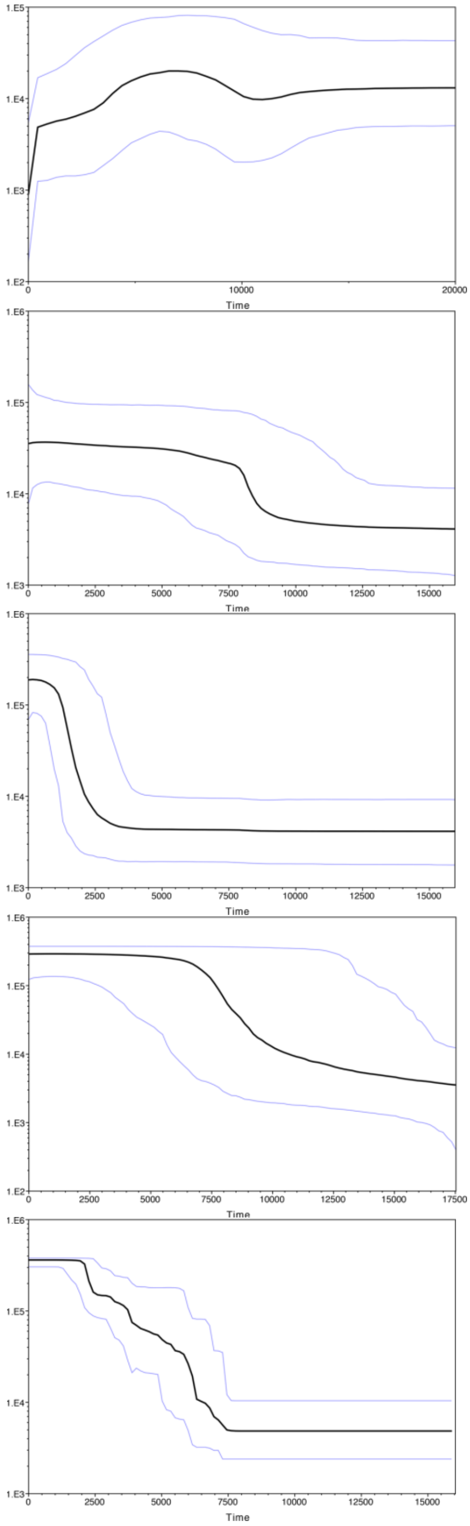

A) Haplogroup P

B) Haplogroup Q

C) Haplogroup T1

D) Haplogroup T2

E) Haplogroup T3

Figure S6. Skyplots. Analysis reveals demographic trends of observed haplogroups calculated using the highly variable part of the D-loop region including all available ancient carbon dated sequences for: A) haplogroup P; B) haplogroup Q; C) haplogroup T1; D) haplogroup T2; E) haplogroup T3.

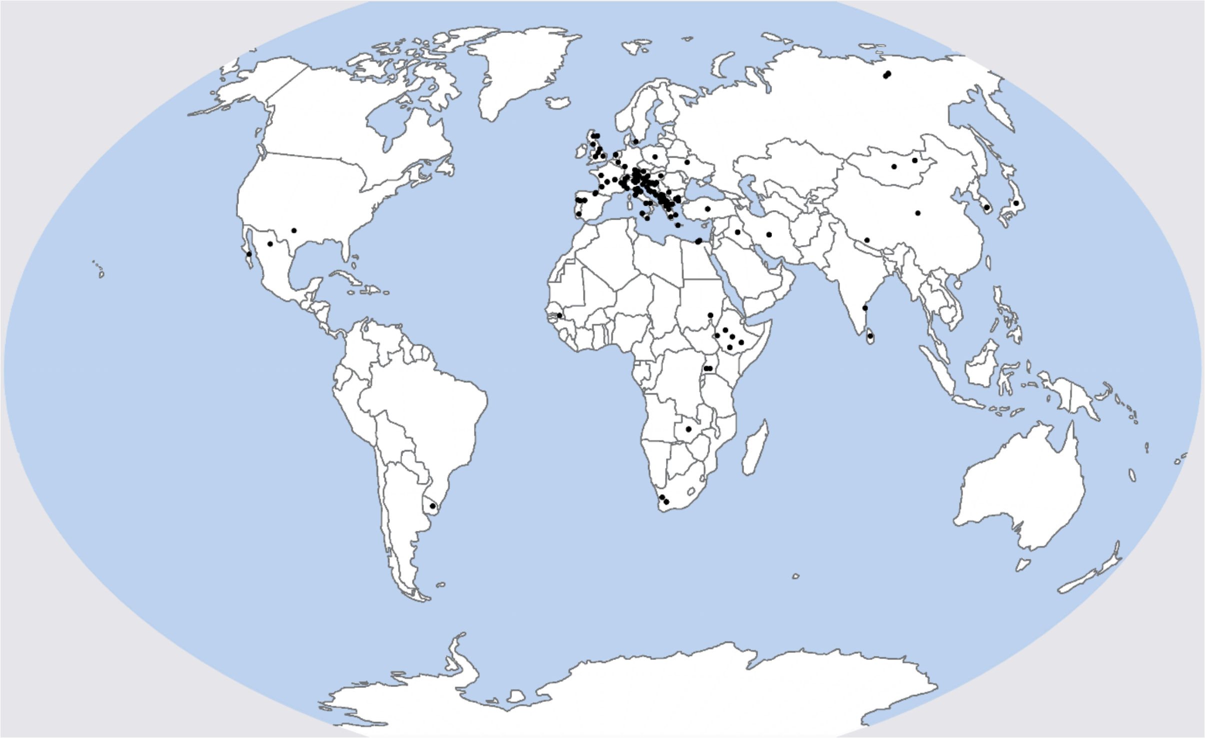

Figure S7. The geographical origin of modern cattle breeds. The geographical distribution of world modern cattle breeds' mitogenome sequences used in the analyses.

Supplementary References

1. Achilli, A., Olivieri, A., Pellecchia, M., Uboldi, C., Colli, L., Al-Zahery, N., Accetturo, M., Pala, M., Kashani, B. H., Perego, U. A., Battaglia, V., Fornarino, S., Kalamati, J., Houshmand, M., Negrini, R., Semino, O., Richards, M., Macaulay, V., Ferretti, L., Bandelt, H. J., Ajmone-Marsan, P., Torroni, A. (2008). Mitochondrial genomes of extinct aurochs survive in domestic cattle. *Current Biology*, **18**(4), R157-R158.

2. Achilli, A., Bonfiglio, S., Olivieri, A., Malusà, A., Pala, M., Kashani, B. H., Perego, U. A., Ajmone-Marsan, P., Liotta, L., Semino, O., Bandelt, H. J., Ferretti, L., Torroni, A. (2009). The multifaceted origin of taurine cattle reflected by the mitochondrial genome. *PloS One*, **4**(6), e5753.

3. Anderson, S. M. H. L., De Bruijn, M. H. L., Coulson, A. R., Eperon, I. C., Sanger, F., & Young, I. G. (1982). Complete sequence of bovine mitochondrial DNA conserved features of the mammalian mitochondrial genome. *Journal of Molecular Biology*, **156**(4), 683-717.

4. Bonfiglio, S., Achilli, A., Olivieri, A., Negrini, R., Colli, L., Liotta, L., Ajmone-Marsan, P., Torroni, A., Ferretti, L. (2010). The enigmatic origin of bovine mtDNA haplogroup R: sporadic interbreeding or an independent event of Bos primigenius domestication in Italy? *PloS One*, **5**(12), e15760.

5. Bonfiglio, S., Ginja, C., De Gaetano, A., Achilli, A., Olivieri, A., Colli, L., Tesfaye, K., Hassan Agha, S., Gama, L. T., Cattonaro, F., Penedo, C. M. T., Ajmone-Marsan, P., Torroni, A., Ferretti, L. (2012). Origin and spread of Bos taurus: new clues from mitochondrial genomes belonging to haplogroup T1. *PloS One*, **7**(6), e38601.

6. Bro-Jørgensen, M. H., Carøe, C., Vieira, F. G., Nestor, S., Hallström, A., Gregersen, K. M., Etting, V., Gilbert, T. M. P., Sinding, M. H. S. (2018). Ancient DNA analysis of Scandinavian medieval drinking horns and the horn of the last aurochs bull. *Journal of Archaeological Science*, **99**, 47-54.

7. Chung, H. Y., & Ha, J. M. Haplotype analysis of mitochondrial DNA in Korean native cattle. *Unpublished, https://www.ncbi.nlm.nih.gov/*

8. De Gaetano, A., Bonfiglio, S., Colli, L., Ajmone-Marsan, P., Ferretti, L. A bovine mitochondrial DNA with a 44 bp insertion in the control region. *Unpublished,* https://www.ncbi.nlm.nih.gov/

9. Douglas, K. C., Halbert, N. D., Kolenda, C., Childers, C., Hunter, D. L., Derr, J. N. (2011). Complete mitochondrial DNA sequence analysis of Bison bison and bison–cattle hybrids: Function and phylogeny. *Mitochondrion*, **11**(1), 166-175.

10. Edwards, C. J., Magee, D. A., Park, S. D., McGettigan, P. A., Lohan, A. J., Murphy, A., Finlay, E. K., Shapiro, B., Chamberlain, A. T., Richards, M. B., Bradley, D. G., Loftus, B. J., MacHugh, D. E. (2010). A complete mitochondrial genome sequence from a mesolithic wild aurochs (Bos primigenius). *PloS One*, **5**(2), e9255.

11. Hiendleder, S., Lewalski, H., & Janke, A. (2008). Complete mitochondrial genomes of Bos taurus and Bos indicus provide new insights into intra-species variation, taxonomy and domestication. *Cytogenetic and Genome Research*, **120**(1-2), 150-156.

12. Horsburgh, K. A., Prost, S., Gosling, A., Stanton, J. A., Rand, C., Matisoo-Smith, E. A. (2013). The genetic diversity of the Nguni breed of African Cattle (Bos spp.): complete mitochondrial genomes of haplogroup T1. *PloS One*, **8**(8), e71956.

13. Lee, K. T., Ahn, S.M., Lee, S.Y., Lee, J., Kim, T. H. A complete Hanwoo mitochondrial genome sequence. *Unpublished.* https://www.ncbi.nlm.nih.gov/

14. Zeyland, J., Wolko, L., Bocianowski, J., Szalata, M., Slomski, R., Dzieduszycki, A. M., Ryba, M., Przystalowska, H., Lipinski, D. (2013). Complete mitochondrial genome of wild aurochs (Bos primigenius) reconstructed from ancient DNA. *Polish Journal of Veterinary Sciences*, **16**(2).

15. Liu, D., Wei, Y., & Guo, Y. (2016). Complete mitochondrial genome of the hybrid of Simmental and Qinghai local cattle breed (Bos taurus). *Mitochondrial DNA Part A*, **27**(6), 4073-4074.

16. Lopez-Oceja, A., Muro-Verde, A., Gamarra, D., Cardoso, S., & de Pancorbo, M. M. (2016). New Q lineage found in bovine (Bos taurus) of Iberian Peninsula. *Mitochondrial DNA Part A*, **27**(5), 3597-3601.

17. Ludwig, A., Lieckfeldt, D., Hesse, U. G., Froelich, K. (2016). Tracing the maternal roots of the domestic Red Mountain Cattle. *Mitochondrial DNA Part A*, **27**(2), 1080-1083.

18. Mannen, H., Morimoto, M., Oyama, K., Mukai, F., & Tsuji, S. (2003). Identification of mitochondrial DNA substitutions related to meat quality in Japanese Black cattle. *Journal of Animal Science*, **81**(1), 68-73.

19. Marsolier-Kergoat, M. C., Palacio, P., Berthonaud, V., Maksud, F., Stafford, T., Bégouën, R., & Elalouf, J. M. (2015). Hunting the extinct steppe bison (Bison priscus) mitochondrial genome in the Trois-Freres Paleolithic Painted Cave. *PloS One*, **10**(6), e0128267.

20. Massilani, D., Guimaraes, S., Brugal, J. P., Bennett, E. A., Tokarska, M., Arbogast, R. M., Baryshnikov, G., Boeskorov, G., Castel, J. C., Davydov, S., Madelaine, S., Putelat, O., Spasskaya, N. N., Uerpmann, H. P., Grange, T., Geigl, E. M. (2016). Past climate changes, population dynamics and the origin of Bison in Europe. *BMC Biology*, **14**(1), 1-17.

21. Miretti, M. M., Ferro, M. I., Meirelles, F., Garcia, J. M., Smith, L. C. The complete mitochondrial genome nucleotide sequence of Bos indicus. *Unpublished.* https://www.ncbi.nlm.nih.gov/

22. Olivieri, A., Gandini, F., Achilli, A., Fichera, A., Rizzi, E., Bonfiglio, S., Battaglia, V., Brandini, S., De Gaetano, A., El-Beltagi, A., Lancioni, H., Agha, S., Semino, O., Ferretti, L., Torroni, A. (2015). Mitogenomes from Egyptian cattle breeds: new clues on the origin of haplogroup Q and the early spread of Bos taurus from the Near East. *PloS One*, **10**(10), e0141170.

23. Shahid, S. A:, Khan, S., Feng, D., Johanson, G. S. Sequence Diversity in the Bos taurus Mitochondrial Genome. *Unpublished.* https://www.ncbi.nlm.nih.gov/

24. Shin, H. D:, Kim, L. H. Bos taurus mitochondrion, complete genome sequence of Korean cattle, beef cattle and Holstein-Friesian. *Unpublished.* https://www.ncbi.nlm.nih.gov/

25. Wang, Z., Shen, X., Liu, B., Su, J., Yonezawa, T., Yu, Y., Guo, S., Ho, S. Y. W., Vila, C., Hasegawa, M., Liu, J. Deep genetic divergences within domesticated yak are the result of quaternary climatic oscillations and orogeny on the Qinghai-Tibetan Plateau. *Unpublished.* https://www.ncbi.nlm.nih.gov/

26. Wang, X., Zhang, Y. Q., He, D. C., Yang, X. M., Li, B., Wang, D. C., Guang, J., Xu, F., Li, J. Y., Gao, X., Gao, H. J., Zhang, L. P., Zhang, X. Z. (2016). The complete mitochondrial genome of Bos taurus coreanae (Korean native cattle). *Mitochondrial DNA Part A*, **27**(1), 120-121.

27. Zeyland, J., Lipniski, D., Przystalowska, H., Szalata, M. Direct Submission. *Unpublished.* https://www.ncbi.nlm.nih.gov/

28. Zhang, H., Paijmans, J. L., Chang, F., Wu, X., Chen, G., Lei, C., Yang, X., Wei, Z., Bradley, D. G., Orlando, L., O'Connor, T., Hofreiter, M. (2013). Morphological and genetic evidence for early Holocene cattle management in northeastern China. *Nature Communications*, **4**(1), 1-7.

29. Kim, J., Hanotte, O., Mwai, O. A., Dessie, T., Bashir, S., Diallo, B., Agaba, M., Kim, K., Kwak, W., Sung, S., Seo, M., Jeong, H., Kwon, T., Taye, M., Song, K. D., Lim, D., Cho, S., Lee, H. J., Yoon, D., Oh, S. J., Kemp, S., Lee, H. K., Kim, H. (2017). The genome landscape of indigenous African cattle. *Genome Biology*, **18**(1), 1-14.

30. Stothard, P., Liao, X., Arantes, A. S., De Pauw, M., Coros, C., Plastow, G. S., Sargolzaei, M., Crowley, J. J., Basarab, J. A., Schenkel, F., Moore, S., Miller, S. P. (2015). A large and diverse collection of bovine genome sequences from the Canadian Cattle Genome Project. *Gigascience*, **4**(1), s13742-015.

31. Anderung, C., Bouwman, A., Persson, P., Carretero, J. M., Ortega, A. I., Elburg, R., Smith, C., Arsuaga, J. L., Ellegren., H., Götherström, A. (2005). Prehistoric contacts over the Straits of Gibraltar indicated by genetic analysis of Iberian Bronze Age cattle. *Proceedings of the National Academy of Sciences*, **102**(24), 8431-8435.

32. Bollongino, R., Burger, J., Powell, A., Mashkour, M., Vigne, J. D., Thomas, M. G. (2012). Modern taurine cattle descended from small number of Near-Eastern founders. *Molecular Biology and Evolution*, **29**(9), 2101-2104.

33. Campana, M. G., Bower, M. A., Bailey, M. J., Stock, F., O'Connell, T. C., Edwards, C. J., Checkley-Scott, C., Knight, B., Spencer, M., Howe, C. J. (2010). A flock of sheep, goats and cattle: ancient DNA analysis reveals complexities of historical parchment manufacture. *Journal of Archaeological Science*, **37**(6), 1317-1325.

34. Edwards, C. J., Bollongino, R., Scheu, A., Chamberlain, A., Tresset, A., Vigne, J. D., Baird, J. F., Larson, G., Ho, S. Y. W., Heupink, T. H., Shapiro, B., Freeman, A. R., Thomas, M. G., Arbogast, R. M., Arndt, B., Bartosiewicz, L., Benecke, N., Budja, M., Chaix, L., Choyke, A. M., Coqueugniot, E., Dohle, H. J., Goldner, H., Hartz, S., Helmer, D., Herzig, B., Hongo, H., Mashkour, M., Ozdogan, M., Pucher, E., Roth, G., Schade-Lindig, S., Schmolcke, U., Schulting, R. J., Stephan, E., Uerpmann, H. P., Voros, I., Voytek, B., Bradley, D. G.

Burger, J. (2007). Mitochondrial DNA analysis shows a Near Eastern Neolithic origin for domestic cattle and no indication of domestication of European aurochs. *Proceedings of the Royal Society B: Biological Sciences*, **274**(1616), 1377-1385.

35. Gravlund, P., Aaris-Sørensen, K., Hofreiter, M., Meyer, M., Bollback, J. P., & Noe-Nygaard, N. (2012). Ancient DNA extracted from Danish aurochs (Bos primigenius): genetic diversity and preservation. *Annals of Anatomy-Anatomischer Anzeiger*, **194**(1), 103-111.

36. Kim, S. J., Suh, J. Y., Kim, K. C., Suh, D. S. The Genetic Characterization of Korean Cattle, Hanwoo, determined by using sequencing of Mitochondrial DNA D-loop region. unpublished. *Unpublished.* https://www.ncbi.nlm.nih.gov/

37. Kühn, R., Ludt, C., Manhart, H., Peters, J., Neumair, E., Rottmann, O. (2005). Close genetic relationship of early Neolithic cattle from Ziegelberg (Freising, Germany) with modern breeds. *Journal of Animal Breeding and Genetics*, **122**, 36-44.

38. Niemi, M., Bläuer, A., Iso-Touru, T., Harjula, J., Edmark, V. N., Rannamäe, E., Lõugas, L., Sajantila, A., Lidén, K., Taavitsainen, J. P. (2015). Temporal fluctuation in North East Baltic Sea region cattle population revealed by mitochondrial and Y-chromosomal DNA analyses. *PloS One*, **10**(5), e0123821.

39. Noda, A., Yonesaka, R., Sasazaki, S., & Mannen, H. (2018). The mtDNA haplogroup P of modern Asian cattle: A genetic legacy of Asian aurochs?. *Plos One*, **13**(1), e0190937.

40. Priskin, K., Tömöry, G., Bogácsi-Szabó, E., Csányi, B., & Raskó, I. (2007). Mitochondrial DNA control region analysis of a late Neolithic aurochs (Bos primigenius Boj. 1827) from the Carpathian Basin. *Acta Biologica Hungarica*, **58**(Supplement-1), 131-137.

41. Pruvost, M., Schwarz, R., Correia, V. B., Champlot, S., Braguier, S., Morel, N., Fernandez-Jalvo, Y., Grange, T., Geigl, E. M. (2007). Freshly excavated fossil bones are best for amplification of ancient DNA. *Proceedings of the National Academy of Sciences*, **104**(3), 739-744.

42. Salamon, M., Tuross, N., Arensburg, B., Weiner, S. (2005). Relatively well preserved DNA is present in the crystal aggregates of fossil bones. *Proceedings of the National Academy of Sciences*, **102**(39), 13783-13788.

43. Scheu, A., Powell, A., Bollongino, R., Vigne, J. D., Tresset, A., Çakırlar, C., Benecke, R., Burger, J. (2015). The genetic prehistory of domesticated cattle from their origin to the spread across Europe. *BMC Genetics*, **16**(1), 1-11.

44. Schibler, J., Elsner, J., Schlumbaum, A. (2014). Incorporation of aurochs into a cattle herd in Neolithic Europe: single event or breeding? *Scientific Reports*, **4**(1), 1-6.

45. Shi, J., Qiao, H., Hosoi, E., Ozawa, S. (2004). Phylogenetic relationship between the Yellow cattle in Qinghai province, China, and Japanese Black cattle based on mitochondrial DNA D-loop sequence polymorphism. *Animal Science Journal (Japan)*.

46. Stock, F., Edwards, C. J., Bollongino, R., Finlay, E. K., Burger, J., Bradley, D. G. (2009). Cytochrome b sequences of ancient cattle and wild ox support phylogenetic complexity in the ancient and modern bovine populations. *Animal Genetics*, **40**(5), 694-700.

47. Mannen, H., Yonezawa, T., Murata, K., Noda, A., Kawaguchi, F., Sasazaki, S., Olivieri, A., Achilli, A., Torroni, A. (2020). Cattle mitogenome variation reveals a post-glacial expansion of haplogroup P and an early incorporation into northeast Asian domestic herds. *Scientific Reports*, **10**(1), 1-7.

48. Verdugo, M. P., Mullin, V. E., Scheu, A., Mattiangeli, V., Daly, K. G., Delser, P. M., Hare, A. J., Burger, J., Collins, M. J., Kehati, R., Hesse, P., Fulton, D., Sauer, E. W., Mohaseb, F. A., Davoudi, H., Khazaeli, R., Lhuillier, J., Rapin, C., Ebrahimi, S., Khasanov, M., Vahidi, F. S. M., MacHugh D. E., Ertuğrul, O., Koukouli-Chrysanthaki, C., Sampson, A., Kazantzis, G., Kontopoulos, I., Bulatovic, J., Stojanović, I., Mikdad, A., Benecke, N., Linstädter, J., Sablin, M., Bendrey, R., Gourichon, L., Arbuckle, B. S., Mashkour, M., Orton, D., Horwitz, L. K., Teasdale, M. D., Bradley, D. G. (2019). Ancient cattle genomics, origins, and rapid turnover in the Fertile Crescent. *Science*, **365**(6449), 173-176.

49. Xia, X., Qu, K., Li, F., Jia, P., Chen, Q., Chen, N., Zhang, J., Chen, H., Huang, B., Lei, C. (2019). Abundant genetic diversity of Yunling Cattle based on mitochondrial genome. *Animals*, **9**(9), 641.

50. Xia, X. T., Achilli, A., Lenstra, J. A., Tong, B., Ma, Y., Huang, Y. Z., Han, J. L., Sun, Z. Y., Chen, H., Lei, C. Z., Hu, S. M., Chen, N. B. (2021). Mitochondrial genomes from modern

and ancient Turano-Mongolian cattle reveal an ancient diversity of taurine maternal lineages in East Asia. *Heredity*, **126**(6), 1000-1008.
